# Hyaluronic acid and tissue mechanics orchestrate mammalian digit tip regeneration

**DOI:** 10.1126/science.ady3136

**Published:** 2026-04-09

**Authors:** Byron W.H. Mui, Joseph J.Y. Wong, Camille E. Dumas, Jia Hua Wang, Toni Bray, Kentaro Hirose, Lauren Connolly, Alexander Winkel, Sebastian Timmler, Nicholas A. Bright, Evelina Sliauteryte, Ragnhildur Thóra Káradóttir, Pamela G. Robey, Kristian Franze, Kevin J. Chalut, Mekayla A. Storer

**Affiliations:** 1 Cambridge Stem Cell Institute, University of Cambridge, Cambridge, UK; 2 Skeletal Biology Section, National Institute of Dental and Craniofacial Research, National Institutes of Health, Department of Health and Human Services, Bethesda, MD, USA; 3 NIH Oxford-Cambridge Scholars Program, National Institutes of Health, Department of Health and Human Services, Bethesda, MD, USA; 4 Stanford Cardiovascular Institute, Stanford University School of Medicine, Stanford, CA, USA; 5 Department of Physiology, Development and Neuroscience, University of Cambridge, Cambridge, UK; 6 Max-Planck-Zentrum für Physik und Medizin, Erlangen, Germany; 7 Medical Institute of Biophysics, Friedrich-Alexander-Universität Erlangen-Nürnberg, Erlangen, Germany; 8 Cambridge Institute for Medical Research, University of Cambridge, Cambridge, UK; 9 Cyclana Bio, Babraham Institute, Cambridge, UK; 10 Department of Physiology, BioMedical Center, Faculty of Medicine, University of Iceland, 101 Reykjavik, Iceland

## Abstract

Tissue regeneration is rare in mammals, but the mouse digit tip can regrow after amputation, while injuries beyond the nail do not. How the microenvironment drives divergent outcomes remains unclear. Here, we found that the extracellular matrix (ECM) and tissue mechanics govern the amputation response in mouse digits. Non-regenerative regions were stiffer and contained dense, organized collagen, whereas regenerative regions were soft and enriched in hyaluronic acid (HA). Depleting HA inhibited regeneration and promoted fibrosis, demonstrating that the HA-collagen balance shaped tissue mechanics and repair signaling. Stabilization of HA with hyaluronan and proteoglycan link protein 1 (HAPLN1) after non-regenerative amputations tuned ECM mechanics, reduced scarring, and enhanced bone repair. Thus, ECM composition and mechanics influence cell behavior and ECM-targeted strategies could help unlock mammalian regeneration.

Although normal wound healing relies upon the synthesis of extracellular matrix (ECM) as provisional scaffolding, excessive accumulation of ECM, known as fibrosis, disrupts tissue architecture and leads to substantial disease burden (1). Developing effective treatments to halt or reverse fibrosis in favor of regeneration hinges on our understanding of wound healing (2). To that end, amputation of mouse digits presents an opportunity to examine mammalian regeneration and fibrosis in parallel (3). Amputation of the tip of a digit’s third phalanx (P3) results in complete, multi-tissue restoration in rodents and humans (3). Central to this process is the blastema (4), a transient structure harboring heterogeneous progenitors (5, 6) that restores lost tissues (7). More proximal amputations, such as those that sever the second phalanx (P2), fail to regenerate (3). Instead, they exhibit minimal regrowth, and scar tissue overlies the injured bone stump (3). Here, we investigated how the ECM may drive regeneration instead of fibrosis in the adult digit amputation model.

The ECM provides essential chemical and mechanical information within various biological contexts, including tissue morphogenesis (8), homeostasis (9) and aging (10). The ECM can impact regeneration (11) and fibrosis (12). Studies of scarless fetal cutaneous injuries implicated a protective role for the ECM component hyaluronic acid (HA), a linear, non-sulfated polymer of *N*-acetyl-glucosamine and glucuronic acid (13). Indeed, fetal wounds heal with minimal collagen deposition while sustaining high levels of HA, whereas adult wounds show extensive deposition of collagen concurrent with rapid degradation of HA (14–16). Following forelimb amputations in salamanders, HA-rich matrices that emerge during blastema formation instruct myotube migration and dedifferentiation (17), and in *Xenopus*, disruption of HA signaling impairs tail regeneration (18). How HA and ECM mechanics jointly govern regenerative versus fibrotic outcomes in adult mammals remains poorly understood.

In fibrotic wound repair, the ECM typically stiffens (19), which mechanically activates fibroblasts (20), the main cellular regulators of the ECM. Fibroblasts further remodel their extracellular environment (21) by way of changes to ECM composition (22) or organization (23), which alter overall tissue mechanics and establish an iterative feedback loop. The blastema’s matrix-derived mechanical cues regulate regenerative cell behavior, such as proliferation or differentiation (24, 25). Later, processes such as apoptosis (26) of fibroblasts restore tissue homeostasis. However, fibrosis progression bypasses these pathways, leading to cumulative disruption of tissue architecture. Given that fibroblasts are heterogeneous (27), some evidence suggests an intrinsic propensity for fibrosis by specific sub-types of fibroblasts (28), which may be mostly absent from regenerative tissues. It is also possible that fibrosing and regenerating ECM niches are distinct and enable opposing repair mechanisms.

## The niche diverges during digit non-regeneration and regeneration

**Fig. 1:**
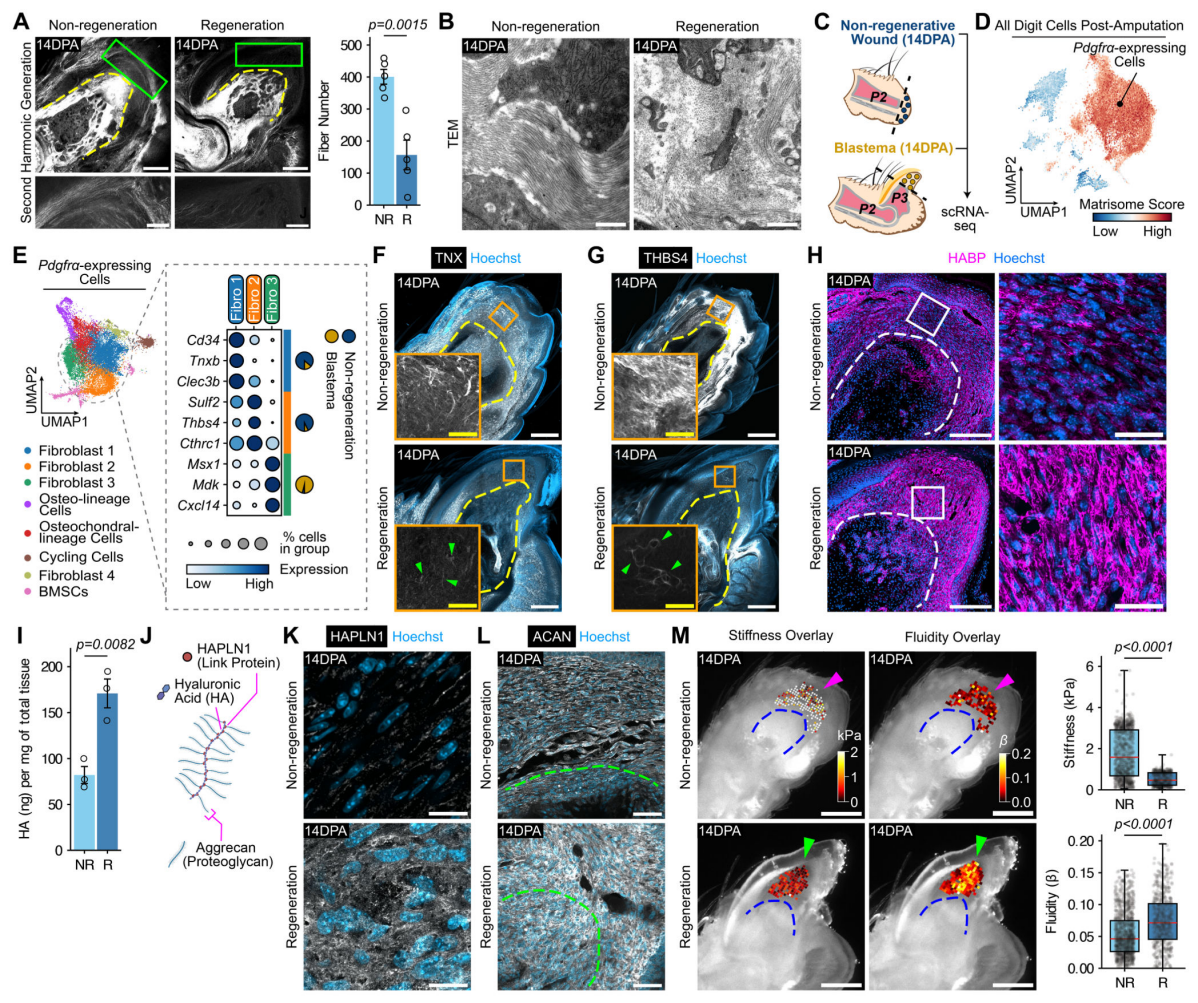
The niche discriminates regeneration from fibrosis after digit tip amputation. (**A**) Left, second harmonic generation microscopy showing collagen fibers (white) in non-regenerative and regenerative digits 14 days post-amputation (DPA). Dashed line, border of the phalanx bone. Right, quantification of collage fiber number in the non-regenerative wound (NR) versus regenerative blastema (R). n = 5 mice per condition. Scale bars, 250 μm and 100 μm in magnified views. (**B**) Transmission electron microscopy (TEM) of NR and R wounds. Scale bars, 1 μm. (**C**) Single-cell RNA sequencing (scRNA-seq) strategy for characterizing non-regenerative wound and blastema cells 14DPA. (**D**) UMAP of all digit cells after amputation scored by their expression of core matrisome genes. (**E**) Left, sub-type analysis of *Pdgfrα*-expressing cells. Right, dot plot of top differentially expressed genes, and pie charts showing their relative proportion in non-regenerative and blastema conditions. (**F-H**) Immunofluorescence showing TNX (white) in F, THBS4 (white) in G and HABP (magenta) in H 14DPA. Green arrowheads, TNX and THSB4 in the blastema. Scale bars, 250 μm and 50 μm in magnified views in F and G; 200 μm and 35 μm in magnified views in H. (**I**) Quantification of total HA per mg of NR versus R tissue. n = 3 replicates per condition (4 mice pooled per replicate). (**J**) Diagram of HA-HAPLN1-ACAN complex. (**K** and **L**) Immunofluorescence showing HAPLN1 (white) in K and ACAN (white) in L 14DPA. Scale bars, 10 μm (K); 50 μm (L). (**M**) Left, atomic force microscopy stiffness and fluidity maps of injured digits. Magenta arrowhead, the non-regenerative wound; green, the blastema. Scale bars, 500 μm. Right, quantification of the stiffness and fluidity in NR versus R. n = 4 mice per condition. Data are mean ± SEM or median and quartiles (AFM data). Data are representative of at least three independent experiments. Statistical significance was determined by two-tailed unpaired student’s *t*-test (A and I) or Mann-Whitney test (M). Additional details on statistics and reproducibility are in the Materials and Methods. See figs. S1-3 for additional supporting experiments. Schematic in J was created using BioRender (https://biorender.com).

Fibrous tissue accumulates after non-regenerative amputations (3), We used second harmonic generation microscopy to investigate the collagen matrix after digit amputations. Non-regenerative wounds contained dense, fibrosis-like collagen, which was largely absent from the blastema (Fig. 1A and fig. S1A and B). We confirmed the collagen’s architectural features by transmission electron microscopy, which similarly showed more organized fiber bundles in non-regeneration (Fig. 1B and fig. S1C). To investigate what ECM factors underlie the difference in the collagen responses, we performed single-cell transcriptomic analysis of non-regenerative wounds and integrated these datasets with previously published blastema and non-regenerative datasets (5, 6) (Fig. 1C). To identify the primary cell type responsible for the ECM among the seven distinct populations detected (fig. S1D and E), we assigned an ECM score to each cell based upon its expression of core matrisome genes (29) (Fig. 1D). The *Pdgfrα*-expressing cluster showed the highest ECM activity, outpacing all other clusters (Fig. 1D). Gene set enrichment analysis revealed that this cluster upregulated ECM-related pathways, including “Collagen Fibril Organization” and “Extracellular Matrix Organization” (fig. S1F). We deduced that fibroblasts constituted most of the *Pdgfrα*-expressing cluster and were the main cell type involved in establishing the extracellular milieu.

Because fibroblasts are heterogeneous, we hypothesized that non-regenerative and regenerative digit ECMs diverge owing to underlying differences in cellular composition. Sub-setting *Pdgfrα*-expressing cells revealed eight transcriptional signatures, of which Fibroblast 1 and 2 sub-types were preferentially enriched in non-regeneration (Fig. 1E and fig. S1G-I). With no notable transcriptional distinction in fibroblast sub-types based on wound conditions (fig. S1J), we hypothesized that the predominance and activity of Fibroblast 1 and 2 cells accounted for the fibrogenic response. All *Pdgfrα*-expressing clusters expressed fibrillar collagens to a similar degree (fig. S1K). However, we identified elevated collagen modeling factors in the non-regenerative wound, such as tenascin X (TNX) expressed by PDGFR*α*^*+*^CD34^+^CD31^-^ Fibroblast 1 cells (Fig. 1E and F, and fig. S2A and B) and thrombospondin-4 (THBS4) expressed by SULF2^+^ Fibroblast 2 cells (Fig. 1E and G, and fig. S2C). Of note, Fibroblast 3 cells, the major blastema sub-type, highly expressed markers associated with limb regeneration, such as *Msx1* and *Mdk*, but did not differentially express major collagen modeling genes (Fig. 1E). Taken together, Fibroblast 1 and 2 cells and their upregulation of TNX and THBS4, respectively, were specific to non-regeneration, suggesting that collagen maintenance and/or modeling drive the fibrotic response.

Given that fibrosis is the antithesis of regeneration, we hypothesized that the blastema is enriched with cell sub-types responsible for synthesizing regeneration-specific ECM. Our cell sub-type correlation and gene expression analysis demonstrated that Fibroblast 1 and 2 cells were most disparate from osteo-lineage cells (OLs) (figs. S1J and S2D), which were approximately three times more prevalent in the blastema (fig. S1H). Furthermore, OLs had the highest expression of collagens and proteoglycans (fig. S2E), which supports the role of OLs as the blastema’s main ECM modulator. ECM-related gene ontology terms describing OLs included “Glycosaminoglycan Binding”, “Proteoglycan Binding,” and “Hyaluronic Acid Binding,” suggesting an OL microenvironment in which non-collagenous matrix components, particularly hyaluronic acid (HA), appear prominently in the blastema (fig. S2F). OLs highly expressed cartilage-associated genes *Acan, Col2a1*, and *Hapln1*, as well as related transcription factors *Sox6* and *Sox9* (fig. S2F). Indeed, at the tissue level, that OLs were more prevalent in regeneration and co-localized with accumulations of hyaluronic acid (HA) (fig. S2G), reminiscent of pericellular coats or a glycocalyx. We confirmed higher total HA in the blastema compared to the non-regenerative wound (Fig. 1I). Because HA binds proteoglycans through link proteins (30) (Fig. 1J), we assayed the distribution of hyaluronan and proteoglycan link protein 1 (HAPLN1) and aggrecan (ACAN), finding a stronger presence of both in the blastema (Fig. 1K and L). Thus, the blastema’s OLs synthesize copious matrices composed of HA, HAPLN1, and ACAN in the absence of major collagen matrix, forming a distinct ECM niche in regeneration.

Given the importance of ECM composition, particularly collagen, in tissue mechanics (31), we used atomic force microscopy and force-clamp force mapping to investigate the physical properties of wounded digits. We detected mechanical differences between non-regenerative wounds and the blastema, including greater stiffness and lower fluidity in the former (Fig. 1M and fig. S2H). Digits undergoing non-regenerative and regenerative responses had very different ECM microenvironments. To explore whether the divergent niches are a product of pre-existing differences between P2 and P3 cells or arise during wound healing, we performed single-cell analysis of uninjured P2 and P3 digits and integrated these datasets with previously published uninjured and injured datasets (fig. S3A). At baseline, anatomical location strongly segregated *Pdgfrα*-expressing cells (fig. S3B and C). Upon injury, both non-regenerative and regenerative cells upregulated matrisome genes and were transcriptionally more similar to each other than to their uninjured counterparts (fig. S3D). However, only regenerative digits upregulated HA by 10 days post amputation (DPA), whereas HA remained scarce at earlier timepoints and in non-regenerative digits (fig. S3E). Thus, both intrinsic cellular properties and injury-induced ECM remodeling shape the distinct regenerative and non-regenerative niches.

## Digit regeneration requires hyaluronic acid

**Fig. 2:**
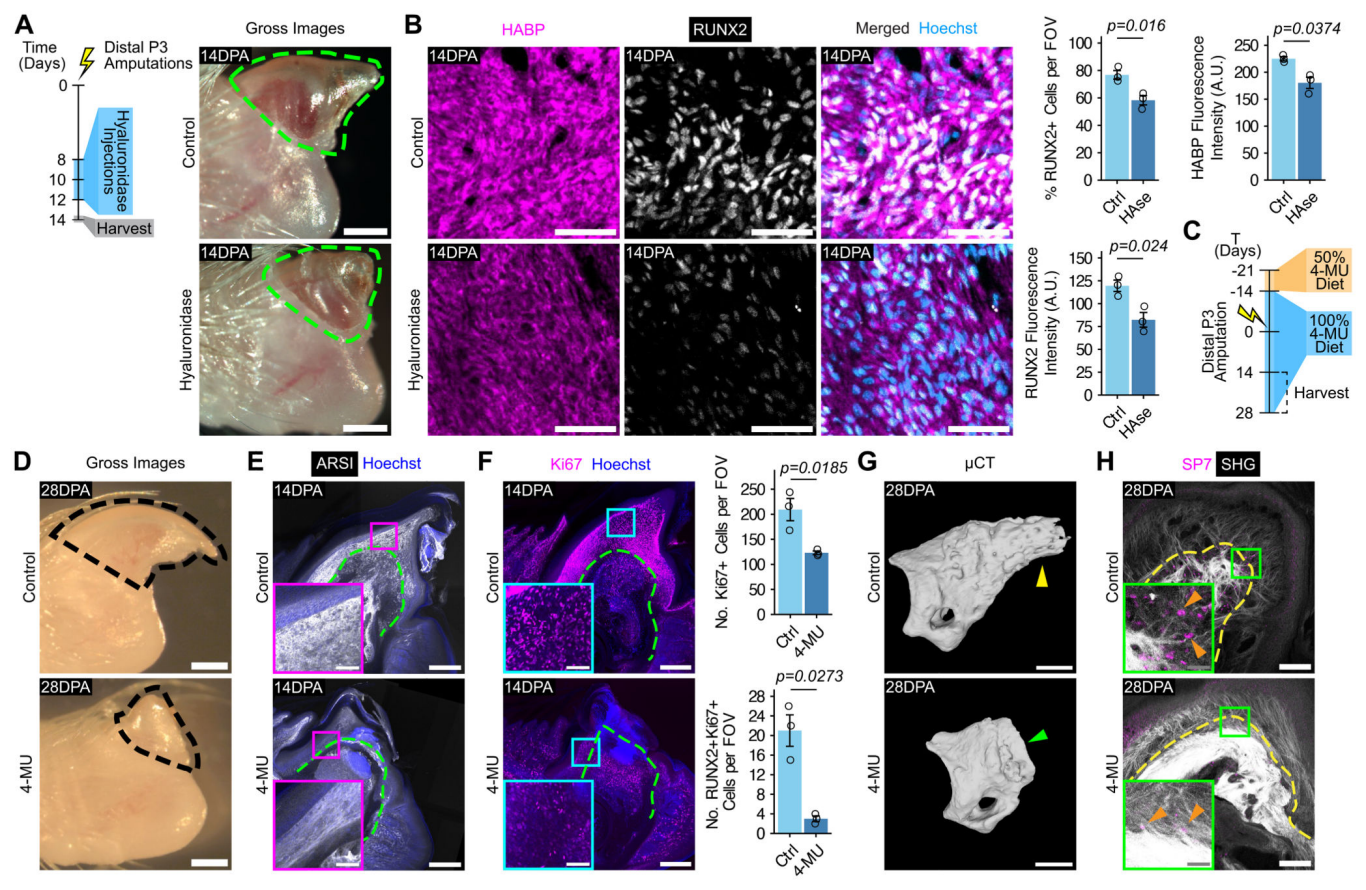
Hyaluronic acid is necessary for successful digit regeneration. **(A)** Left, strategy for degrading hyaluronic acid using hyaluronidase after distal tip (P3) amputations. Right, gross images of control or hyaluronidase-treated digits 14 days post-amputation (DPA), with the nail outlined in green. Scale bars, 100 μm. **(B)** Left, immunofluorescence showing HABP (magenta) and RUNX2 (white) at 14DPA in control and hyaluronidase-treated digits. Scale bar, 50 μm. Right, quantification of percentage RUNX2^+^ cells, HABP signal intensity, and RUNX2 signal intensity after hyaluronidase treatment (HAse) compared to controls. n = 3 mice per condition. **(C)** Strategy for continuously depleting hyaluronic acid using 4-methylumbelliferone (4-MU) after distal P3 amputations. **(D)** Gross images of a control and 4-MU digit 28DPA, with the nail outlined in black. Scale bars, 100 μm. **(E and F)** Immunofluorescence showing ARSI (white) in E and Ki67 (magenta) in F 14DPA. The dashed line, the border of the phalanx bone. Scale bars, 250 μm and 50 μm for magnified views. Right, quantification of the number of Ki67^+^ and RUNX2^+^Ki67^+^ cells with 4-MU treatment versus control. n = 3 mice per condition. **(G)** Microcomputed tomography (μCT) analysis of skeletal morphologies of control and 4-MU digits 28DPA. Yellow arrowhead, bone elongation; green arrowhead, bone stump. Scale bars, 500 μm. **(H)** Immunofluorescence showing SP7 (magenta) and second harmonic generation microscopy showing collagen fibers (white) in control and 4-MU digits 28DPA. Orange arrowheads, SP7^+^ nuclei. Data are mean ± SEM and are representative of at least three independent experiments. Statistical significance was determined by two-tailed unpaired student’s *t*-test (B and F). Additional details on statistics and reproducibility are in the Materials and Methods. See fig. S4 for additional supporting experiments. A.U. = arbitrary units; FOV = field of view.

Given the abundance of hyaluronic acid (HA) in the blastema, we asked whether digit regeneration depended upon HA. To answer this question, we degraded HA enzymatically by serial injections of hyaluronidase into the digit following amputations of the tip of the third phalanx (P3) (Fig. 2A). At 14 DPA, digits were markedly decreased in length, and the area was often smaller (Fig. 2A and fig. S4A). RUNX2^+^ osteo-lineage cells (OLs) and pericellular HA were also diminished after hyaluronidase treatment (Fig. 2B), corroborating the relationship between OLs and HA. For continuous HA depletion, we incorporated 4-methylumbelliferone (4-MU) into the mouse diet to reduce levels of UDP-glucuronic acid, one of two substrates required for HA synthesis (32) (Fig. 2C). With reduction of extracellular HA (fig. S4B and C), 4-MU digits at 14 DPA were reduced in length and area (fig. S4D and E). By 28DPA, control digits regenerated, but 4-MU digits remained significantly smaller (Fig. 2D and fig. S4E). We then examined the state of the blastema in 4-MU digits, hypothesizing that the digits’ defects were a result of compromised blastema formation. With 4-MU, digits globally downregulated their expression of the blastema marker arylsulfatase I (ARSI) (6) (Fig. 2E). Another feature of the blastema is the prevalence of proliferative cells (33). The blastema contained high numbers of Ki67^+^ proliferating cells, particularly osteoblasts, which was substantially reduced with 4-MU treatment (Fig. 2F and fig. S4F). The fate of the blastema involves differentiation into mature tissue (7). Using microcomputed tomography, we showed that 14 DPA regenerating digits exhibited histolysis—the expulsion of the distal bone fragment resulting in bone shortening—and new bone formation (fig. S4G and I). 4-MU delayed histolysis-induced bone shortening, and we detected no new bone (fig. S4G and I). By 28 DPA, when regeneration was expected to be largely complete, 4-MU digit bone volume, surface area, and length remained diminished compared to controls (Fig. 2G and fig. S4H and I). The regenerated digit contained an abundant population of SP7^+^ osteoblasts, while 4-MU reduced osteoblast numbers (Fig. 2H and fig. S4J). Additionally, fibrosis-like collagen was deposited in 4-MU digits, indicating a switch to a more non-regenerative ECM (Fig. 2H and fig. S4J). Thus, depletion of HA interfered with digit restoration after distal P3 amputation, likely owing to a failure of blastema formation and differentiation, which suggests an important role for HA in digit tip regeneration.

## HA-collagen balance determines digit repair trajectory

**Fig. 3:**
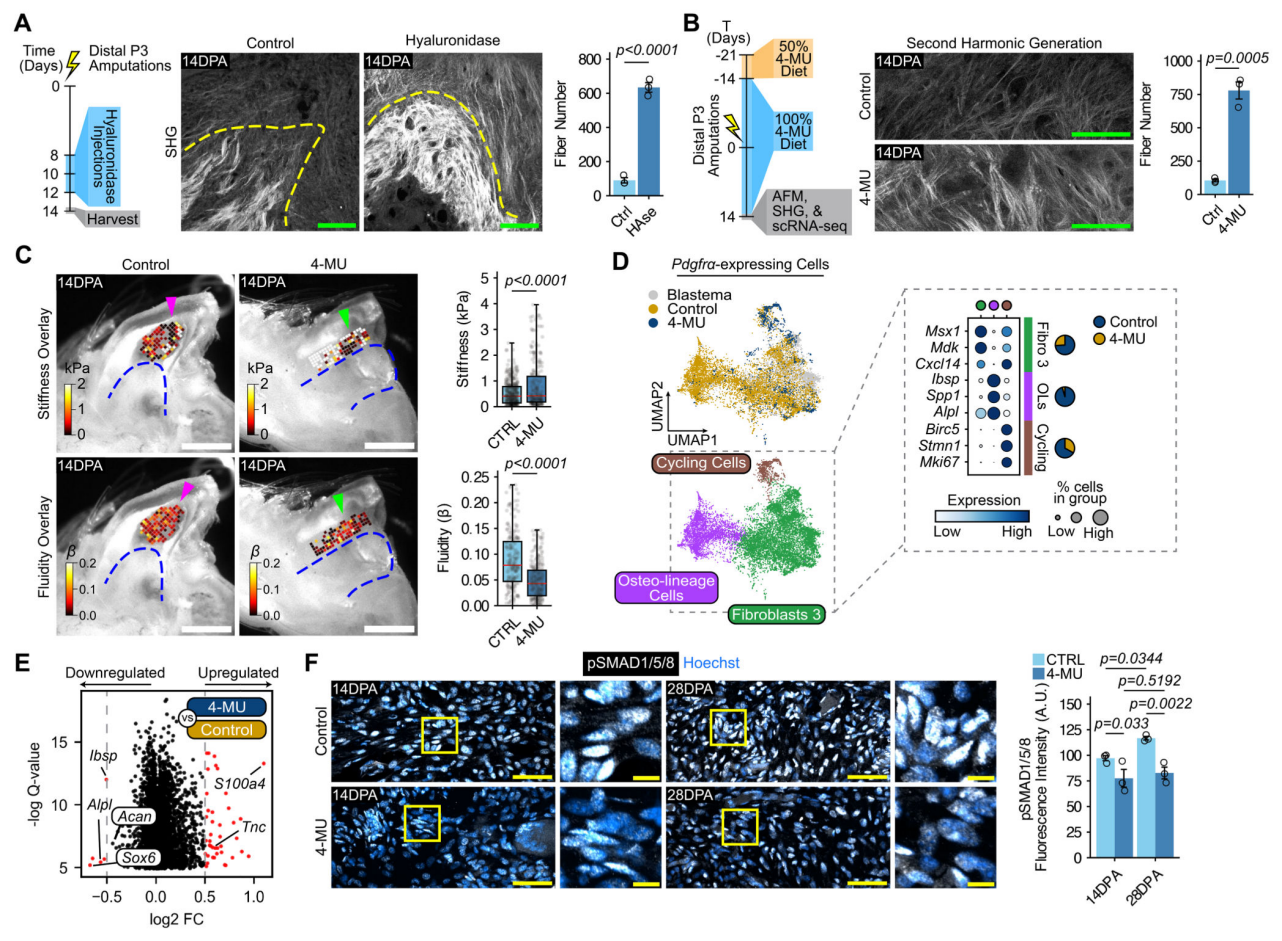
The collagen-hyaluronic acid balance determines the switch between fibrosis and regeneration. (**A** and **B**) Left, strategy to deplete hyaluronic acid using hyaluronidase (A) or 4-methylumbelliferone (4-MU) in B after distal tip (P3) amputations. Middle, second harmonic generation microscopy (SHG) of collagen fibers (white) 14 days post-amputation (DPA) with quantification on the right. Dashed line, border of the phalanx bone. AFM, atomic force microscopy; scRNA-seq, single-cell RNA sequencing. Scale bars, 100 μm. n = 3 mice per condition. (**C**) Left, atomic force microscopy stiffness and fluidity maps of 4-MU digits 14DPA with quantification on the right. Scale bars, 500 μm. n = 4 mice per condition. (**D**) Top, UMAP of *Pdgfrα*-expressing cells from the 4-MU and control datasets. Prior 14DPA blastema datasets were also integrated only to strengthen dimensionality reduction plotting; all downstream analyses were performed comparing 4-MU versus control samples only. Bottom, distinct sub-types detected among *Pdgfrα*-expressing cells. Right, top differentially expressed genes for each *Pdgfrα*-expressing sub-type, along with pie charts representing their relative proportion among all cells. Fibro 3, Fibroblast 3 cells; OLs, osteo-lineage cells. (**E**) Volcano plot of differentially expressed genes in *Pdgfrα*-expressing cells with 4-MU treatment compared to controls. (**F**) Left, immunofluorescence showing pSMAD1/5/8 in 4-MU digits compared to controls 14DPA and 28DPA with quantification on the right. Scale bars, 100 μm and 10 μm in magnified views. Ctrl, control. n = 3 mice per condition. Data are mean ± SEM or median and quartiles (AFM data). Data are representative of at least three independent experiments. Statistical significance was determined by two-tailed unpaired student’s *t*-test (A and B), Mann-Whitney test (C), the two-part generalized linear model MAST with a joint test summing likelihood ratio or Wald test statistics (E) or two-way ANOVA with Tukey’s multiple comparisons test (F). Additional details on statistics and reproducibility are in the Materials and Methods. See fig. S5 for additional supporting experiments.

Given that hyaluronic acid (HA) and collagen were inversely correlated in wounded digits, and HA depletion elicited a fibrosis-like ECM, we asked whether HA matrices abrogate fibrotic collagen assembly. We first compared the collagen matrix of hyaluronidase-treated versus control digits. Hyaluronidase treatment increased collagen content, as well as fibrotic architectural features (Fig. 3A and fig. S5A). We observed similar attributes after HA depletion using 4-MU (Fig. 3B and fig. S5B). Overall, disruption of HA produced fibrotic collagen matrix. Because collagen content impacts tissue mechanics, we hypothesized that the fibrotic collagen emerging with HA depletion would alter the tissue’s mechanical properties. To test this idea, we perturbed HA using 4-MU prior to distal third phalanx (P3) amputations and performed stiffness and fluidity measurements. 4-MU-treated digits were stiffer and less fluid compared to controls (Fig. 3C and fig. S5C). Thus, the viscoelasticity of 4-MU-treated digits closely resembled that of non-regenerating wounds (fig. S5C), highlighting how HA-collagen interactions impact the mechanical microenvironment.

To probe how tissue mechanics arising from HA depletion affected cell behavior, we performed single-cell transcriptomic analysis of distal P3-amputated digits after 4-MU treatment versus control. Unsupervised clustering and integration of the datasets resulted in nine major cell types (fig. S5D). Hyaluronic acid depletion demonstrably reduced the relative proportion of *Pdgfrα*-expressing cells (fig. S5D), indicating a suppression of their typical injury-induced expansion. Analyzing the *Pdgfrα*-expressing cells, we showed a diminished osteo-lineage (OL) proportion after 4-MU treatment (Fig. 3D and fig S5E). Comparing all *Pdgfrα*-expressing cells between conditions underscored the downregulation of the OL-related genes, *Ibsp* and *Alpl*, with HA depletion, as well as *Sox6* and its target gene *Acan* (Fig. 3E). These results highlighted a disturbance in osteogenic differentiation and the downregulation of cartilage-associated matrix elements. Notably, 4-MU-treated *Pdgfra*-expressing cells upregulated their expression of *S100a4* and *Tnc* (Fig. 3E), both of which are associated with increasing ECM stiffness (12, 34). These genes can also be expressed by tenocytes; however, the absence of a tenocyte-associated gene signature with 4-MU suggested that this transcriptional shift does not reflect tenogenic differentiation (fig S5F). Indeed, the few *Pdgfra*-expressing cells that remained also upregulated blastema-associated genes, but their limited abundance indicates a failure of expansion rather than loss of blastema transcriptional identity (fig. S5G).

Given that HA depletion resulted in skeletal defects and disruption of osteogenic differentiation following distal P3 amputations, we explored the relationship between HA, tissue mechanics, and the bone morphogenic protein (BMP) pathway. Unlike previous reports linking HA with Wnt signaling (18), we did not observe changes in Wnt activation in the blastema after HA depletion (fig. S5H). Consequently, we assayed the primary effector of BMP signaling, pSMAD1/5/8, within the tissue after HA depletion with 4-MU. pSMAD1/5/8 levels were elevated and increased as regeneration progressed in control digits (Fig. 3F). In contrast, 4-MU persistently suppressed pSMAD1/5/8 levels (Fig. 3F). Thus, OLs not only help establish the ECM environment in regeneration but are also acutely sensitive to HA and changes in tissue mechanics.

## Substrate stiffness mediates cell-ECM feedback

**Fig. 4:**
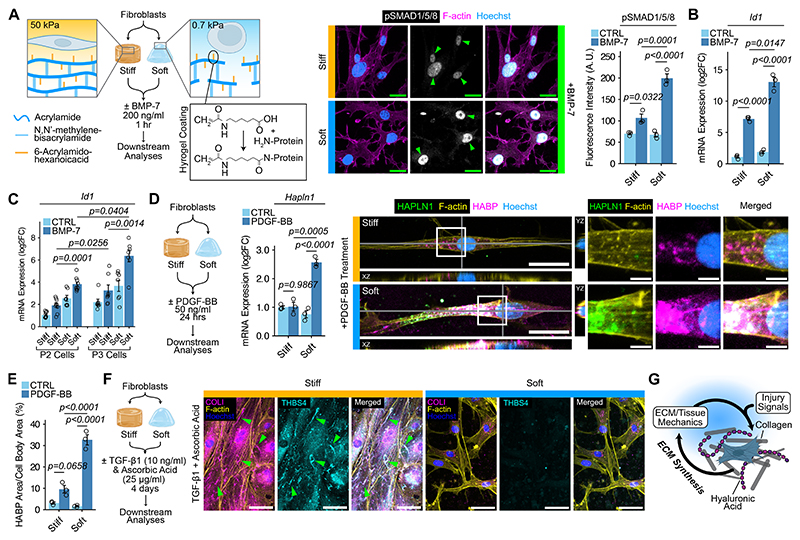
Substrate stiffness regulates feedback between ECM synthesis and response to injury signals. (**A**) Left, strategy to test the effects of substrate stiffness on BMP signaling using StemBond hydrogels. Back skin dermal fibroblasts were used in all hydrogel experiments unless indicated otherwise. Stiff, 50 kPa; soft, 0.7 kPa. Middle, immunofluorescence showing pSMAD1/5/8 (white) and F-actin (magenta) in fibroblasts cultured on stiff and soft hydrogels. Arrowheads, nuclear pSMAD1/5/8. Scale bars, 20 μm. Right, quantification of pSMAD1/5/8 fluorescence intensity. Ctrl, control. n = 3 independent experiments. (**B**) qPCR analysis of *Id1* gene expression. n = 3 independent experiments. (**C**) qPCR analysis of *Id1* gene expression in second phalanx (P2) or third phalanx (P3) cells. n = 9 independent experiments, P2 cells; n = 7 independent experiments, P3 cells. (**D**) Left, strategy to test the effects of substrate stiffness on PDGF-BB signaling. Middle, qPCR analysis of *Hapln1* gene expression. n = 3 independent experiments. Right, immunofluorescence showing HAPLN1 (green), F-actin (yellow) and HABP (magenta) in cultured fibroblasts. Scale bars, 25 μm and 10 μm in magnified views. (**E**) Quantification of HABP area. n = 3 independent experiments. (**F**) Left, strategy to test the effects of substrate stiffness on collagen fibrillogenesis using TGF-β1 and ascorbic acid. Right, immunofluorescence of COLI (magenta), F-actin (yellow) and THBS4 (magenta). Arrowheads, regions of COLI and THBS4. Scale bars, 50 μm. (**G**) Working model of the feedback between ECM, tissue mechanics, and *Pdgfrα*-expressing cells. Data are mean ± SEM and are representative of at least three independent experiments. For all gene expression data, plots are shown as log2FC, with statistical analyses performed on -ΔΔCT values. Statistical significance was determined by two-way (A, B, D, and E) or three-way (C) ANOVA with Tukey’s multiple comparisons test. Additional details on statistics and reproducibility are in the Materials and Methods. See fig. S6 for additional supporting experiments. Schematics in A, D, F, and G were created using BioRender (https://biorender.com).

To dissect the interplay of mechanical cues and soluble signaling, we created stiff and soft StemBond hydrogels (35) to model the mechanical microenvironment of the non-regenerative wound and blastema, respectively (Fig. 4A). Simultaneously, we administered blastema-associated growth factors to examine their combined effects on cellular responses (Fig. 4A). Back skin fibroblasts on soft substrates exhibited enhanced nuclear pSMAD1/5/8 signal after BMP-7 treatment (Fig 4A and fig. S6A). Immunoblotting further corroborated these stiffness-dependent patterns in pSMAD1/5/8 levels (fig. S6B). We also profiled changes in the gene expression of *Inhibitors of DNA Binding/Differentiation* (*Id*), to assess early downstream effectors of pSMADs. *Id1* expression responded to substrate stiffness, with fibroblasts on soft substrates upregulating the highest levels of *Id1* (Fig. 4B and fig. S6C). We found similar trends among freshly isolated cells from the second and third phalanx, which upregulated their expression of *Id1* most when stimulated under soft conditions (Fig. 4C). Given no baseline differences in the ECM in our hydrogel experiments (fig. S6D and E), our data indicated that substrate stiffness tunes responsiveness to BMP ligands.

Next, we used platelet-derived growth factor-BB (PDGF-BB)—a blastema injury signal (36) with potent HA-synthesizing activity (37)—to test how substrate stiffness influences HA (Fig. 4D). In addition to *Hapln1* and *Acan*, we considered the three isoforms of hyaluronan synthases. Substrate stiffness had little effect on expression levels on *Has1*-*3*, and, under these experimental conditions, *Acan* was not responsive to either PDGF-BB or substrate stiffness (fig. S6F-H). In contrast, fibroblasts cultured on soft hydrogels with PDGF-BB treatment significantly upregulated *Hapln1* (Fig. 4D). Reasoning that HAPLN1 confers structural integrity to the HA matrix, we asked whether fibroblasts assemble pericellular HA matrix more robustly under soft conditions. Following PDGF-BB treatment, fibroblasts produced more aggregates of HA and HAPLN1 at the cell-surface on soft in comparison to stiff substrates (Fig. 4D and E, and fig. S6I). Because soft mechanical cues enhance pro-regenerative ECM synthesis, we investigated whether a stiff environment augments non-regenerative ECM and used Transforming growth factor-beta 1 (TGF-β1) and ascorbic acid to induce collagen synthesis (38) (Fig. 4F). After treatment, fibroblasts on stiff substrates produced fibrillar collagen matrix that largely co-localized with thrombospondin-4 (THBS4) (Fig. 4F and fig. S6J), both of which typified non-regenerative healing (Fig. 1A, B, and G). Conversely, in soft conditions that mimic the blastema, collagen I-THBS4 network formation remained undetectable, irrespective of ascorbic acid and TGF-β1 addition (Fig. 4F and fig. S6J). Thus, the softness imbued by HA may initiate positive feedback mechanisms to enhance the production of HA matrix, in contrast to stiff substrate-induced collagen fibrillogenesis (Fig. 4G).

## HAPLN1 facilitates repair of non-regenerative amputations

**Fig. 5:**
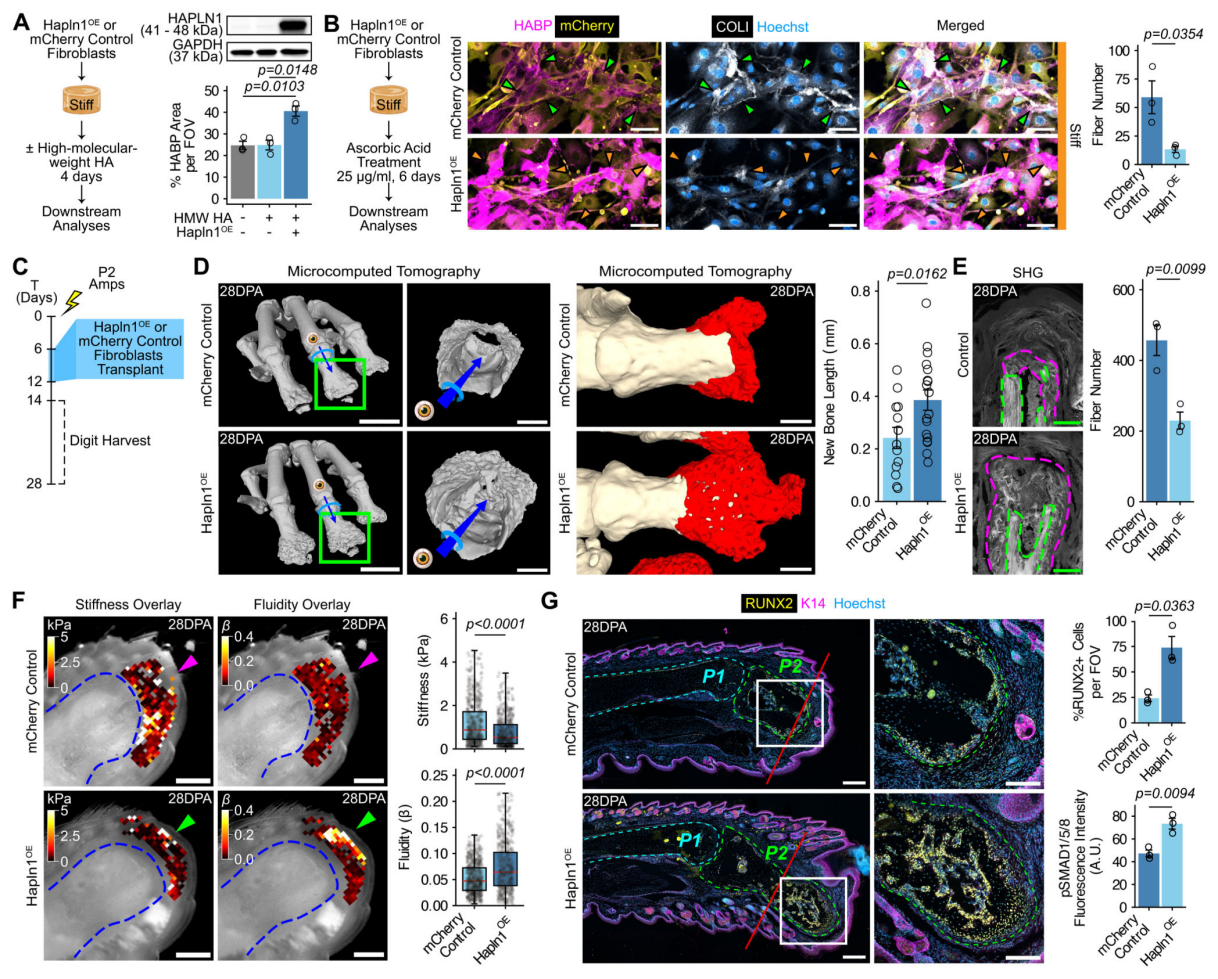
Hyaluronic acid accumulation facilitates digit repair after non-regenerative amputations. (**A**) Left, strategy to test *Hapln1* overexpression (Hapln1^OE^) in a stiff environment. Right, HAPLN1 immunoblot of mCherry Control and Hapln1^OE^ back skin dermal fibroblasts, with quantification of HABP coverage per field of view (FOV). n = 3 independent experiments. (**B**) Left, strategy to assess Hapln1^OE^ effects on collagen fibrillogenesis. Middle, immunofluorescence of HABP (magenta) and COLI (white) with collagen fiber quantification (right). Arrowheads, COLI and HABP staining. Scale bars, 50 μm. n = 3 independent experiments. (**C**) Top, strategy to induce restorative repair of non-regenerative digits. (**D**) Left, microcomputed tomography of digits 28DPA with blinded segmentation (red, middle) and quantification (right). Scale bars, 1 mm, 200 μm or 250 μm, respectively. n = 18 digits per condition. (**E**) Left, second harmonic generation microscopy (SHG) imaging of collagen fibers (white) in digits 28DPA with quantification (right). Dashed lines, pre-existing cortical bone (green) or new bone formation (magenta). Scale bars, 200 μm. n = 3 mice per condition. (**F**) Left, atomic force microscopy stiffness and fluidity maps of Hapln1^OE^ versus mCherry Control digits 14DPA with quantification (right). Dashed lines, P2 bone. Scale bars, 500 μm. n = 4 mice per condition. (**G**) Left, immunofluorescence of digits 28DPA stained for RUNX2 (yellow) and K14 (magenta). Dashed lines, P1 (cyan) and P2 (green). Red line, plane of amputation. Scale bars, 250 μm and 150 μm in magnified views. Right, quantification of RUNX2^+^ cells and pSMAD/1/5/8 fluorescence intensity. n = 3 mice per condition. Data are mean ± SEM or median and quartiles (AFM data) and representative of at least three independent experiments. Statistical significance was determined by one-way ANOVA with Tukey’s multiple comparisons test (A), two-tailed unpaired student’s *t*-test (B, D, E, and G), or Mann-Whitney test (F). Additional details on statistics and reproducibility are shown in the Materials and Methods. See fig. S7-10 for additional supporting experiments. Schematics in A and B were created using BioRender (https://biorender.com). A.U. = arbitrary units; FOV = field of view.

Next, we investigated whether HAPLN1 promotes pericellular HA and impacts the collagen matrix. Not only was HAPLN1 abundant alongside HA in the blastema (Fig. 1H and K), but blastema-like substrate softness also propagated the synthesis of both (Fig. 4D and E, and fig S6I). Analysis of uninjured digits showed that the third phalanx (P3) region contained wide swaths of HA- and HAPLN1-rich regions, which appeared only in specific areas of the second phalanx (P2), such as the periosteum (fig. S7A). We corroborated regional differences in HAPLN1 by qPCR analysis of P2 and P3 cells (fig. S7B). These findings showcased strong associations between HAPLN1 and HA in injury and homeostasis, leading us to hypothesize that HAPLN1 is a key factor mediating the presence of HA. To test this hypothesis, we overexpressed *Hapln1* (Hapln1^OE^) or a scrambled sequence as control (mCherry Control) in back skin fibroblasts (fig. S7C-E). We cultured transduced cells on stiff hydrogels to simulate the fibrotic mechanical environment, with or without the presence of high-molecular-weight HA to encourage pericellular HA accumulation (Fig. 5A and fig. S7F and G). Despite HA supplementation, mCherry Control fibroblasts exhibited sparse and limited distribution of HA across their cell surface (Fig. 5A and fig. S7F and G). Meanwhile, Hapln1^OE^ fibroblasts synthesized large quantities of HAPLN1 that corresponded with a significant increase in pericellular HA (Fig. 5A and fig. S7F and G). Next, we tested whether stabilizing HA matrix using HAPLN1 could inhibit stiffness-induced collagen fibrillogenesis. To mimic the fibrogenic milieu, we cultured transduced fibroblasts on stiff hydrogels and used ascorbic acid to induce collagen fibrillogenesis (Fig. 5B). Hapln1^OE^ fibroblasts accumulated robust pericellular HA that coincided with fewer and shorter collagen fibrils compared to mCherry Control fibroblasts (Fig. 5B and fig. S7H). Thus, HAPLN1 increased the deposition of HA and restrained collagen fibrillogenesis, signifying HAPLN1’s potential to promote a regenerative ECM in a non-regenerative context.

Because regeneration requires HA and HAPLN1 stabilizes the HA matrix, we hypothesized that overexpressing *Hapln1* in non-regenerating wounds would initiate restorative repair. To test this hypothesis, we performed non-regenerative amputations on adult immunocompromised mice and transplanted mCherry Control or Hapln1^OE^ fibroblasts into the digit tip (Fig. 5C and fig. S8A). Notably, we observed one incidence of nail regrowth at 28DPA with Hapln1^OE^ fibroblast transplantation (fig. S8A), which does not occur under non-regenerative conditions. Using microcomputed tomography, we showed enhanced bone repair in digits injected with Hapln1^OE^ fibroblasts, including greater bone elongation and growth (Fig. 5D and fig. S8B). Regenerating bone tissue in Hapln1^OE^ digits extended beyond the initial amputation plane, in contrast to minimal new tissue formation in controls (Fig. 5D and fig. S8B). To confirm that these regenerative effects were independent of cell transplantation, we injected *Hapln1*-overexpressing lentivirus into the digit, which similarly resulted in bone elongation and increased callus size (fig. S9A). Hapln1^OE^ fibroblasts decreased the presence of fibrosis-like collagen structures (Fig. 5E and fig. S8C-E) and promoted HA and HAPLN1 accumulation (fig. S8F and G). Because HA and collagen influence tissue mechanics, we hypothesized that HAPLN1-driven ECM remodeling promotes regeneration by altering the mechanical properties of the microenvironment. Using atomic force microscopy, we found a decrease in stiffness and increase in fluidity at the amputation site of Hapln1^OE^ digits compared to controls (Fig. 5F and fig. S8H).

Finally, we explored how ECM modulation by HAPLN1 affects cellular activity. Because *Sox9* was elevated in the blastema (fig. S2F), we assayed SOX9 distribution as a marker for early chondro-osteogenic commitment. Digits with mCherry Control fibroblasts exhibited low SOX9^+^ cell numbers, whereas clusters of SOX9^+^ cells localized distally from the plane of amputation in digits with Hapln1^OE^ fibroblasts (fig. S8I). We also assayed for more differentiated RUNX2^+^ osteo-lineage (OL) cells. In digits transplanted with mCherry Control fibroblasts, we observed RUNX2^+^ osteo-lineage cells (OLs) only immediately adjacent to the pre-existing cortical bone at 14- and 28DPA (Fig. 5G and fig. S8J and K). However, Hapln1^OE^ fibroblasts increased the presence and distal localization of RUNX2^+^ OLs at 28DPA, consistent with bone regrowth (Fig. 5G and fig. S8K). Furthermore, given that soft substrates enhanced responsiveness to BMP signaling in vitro, we assayed pSMAD/1/5/8 levels and found elevated signal with Hapln1^OE^ compared to controls (Fig. 5G and fig. S8L). Thus, HAPLN1 triggers restorative repair after non-regenerative amputations, likely by mediating the HA-collagen matrices and softening the tissue microenvironment.

To explore whether the HA-collagen dichotomy emerges in other regenerative settings, we examined transcriptomic datasets spanning three models of regeneration: ear pinna injury, myocardial infarction, and fracture healing (fig. S10A-D). Although the specific HA-associated enzymes, crosslinkers, and receptors varied between models, the regenerative transcriptional programs converged on upregulation of HA-related genes and downregulation of fibrillar collagen and crosslinking genes that typify fibrogenesis. Thus, different organs may synthesize and remodel the ECM through tissue-specific mechanisms, and an HA/collagen dichotomy is a shared feature of regenerative versus fibrotic healing.

## Discussion

Our understanding of fibrotic wound healing has grown rapidly in recent years (2). However, factors that orchestrate complex tissue regeneration in vertebrates remain elusive. Part of the challenge lies in the few instances of accessible, robust tissue regeneration models, particularly in adult mammals. By investigating wound healing after adult mouse digit amputations, we examined the relationship between the tissue’s physical environment and regenerative versus fibrotic outcomes. We identified the ECM and resultant tissue mechanics as key properties distinguishing non-regenerative from regenerative responses. Importantly, we found large amounts of hyaluronic acid (HA) in the blastema, which was necessary for regeneration; its absence triggered a switch towards fibrotic ECM and tissue mechanics in the amputated digit. Substrate mechanics, in turn, impacted cellular responses to injury signals, which we propose reinforce wound healing trajectories through feedback loops. Our experiments also demonstrated partial digit restoration after non-regenerative amputations by targeting the ECM, supporting these conclusions.

Fibroblasts are the principal architects of the extracellular matrix and wound healing (20). For example, a distinct fibroblast arises in infarcted hearts, and blocking their activation by immune cells reduces scar formation (39). Similarly, mechanical activation of *Engrailed-1* in specific fibroblasts drives scar formation in skin wounds, and inhibiting this activation promotes regeneration (28, 40). Our study builds on this body of work by examining both the cells and physical niche that distinguish non-regenerative wounds from the regenerative blastema. In the former condition, two distinct *Pdgfrα*-expressing fibroblasts predominated, promoting a stiff collagen matrix through the production of collagen-modeling factors. In contrast, osteo-lineage cells (OLs) contributed to the blastema’s soft, more fluid extracellular milieu by synthesizing HA and HA-associated components, such as ACAN and HAPLN1. These findings highlight the divergence of both cell and extracellular factors during regenerative and non-regenerative processes, as well as the strong interdependence between the two. Moreover, these observations underscore the importance of considering the role of the physical environment, as well as fibroblast diversity, in determining wound healing outcomes.

By targeting hyaluronic acid (HA) through perturbation and rescue experiments, our study provides evidence that HA is essential for regeneration. We posit that HA facilitates wound repair by modulating collagen fibrillogenesis and tissue mechanics. While this phenomenon has been documented in mammalian fetal wounds (14–16, 41) and in other species (25), our findings in adult mice show that HA-collagen interdependency is conserved in injuries across developmental stages and may extend to other organ systems. However, the exact mechanism by which HA mediates collagen assembly remains to be elucidated. One possibility is that bulky pericellular HA matrices regulate integrin accessibility to collagen (42). Although not directly examined in our study, integrin-mediated binding to collagen is a major way by which cells sense and transmit mechanical forces (43) and regulate the presentation of soluble ligands (44); together, these mechanisms govern collagen remodeling. Simultaneously, HA matrices may physically interfere with the self-assembly and organization of collagen polymers (45), possibly through steric hindrance or by limiting diffusion of pro-collagen.

Finally, we showed that the ECM and substrate mechanics regulate both fibrosis and regeneration. Prior studies have also related extrinsic physical forces to cellular rejuvenation and regeneration. For example, brain stiffening with age impairs oligodendrocyte precursor cell function (46). Inhibiting the mechanosensitive ion channel Piezo1 mitigated age-related changes in these cells, enhancing their regenerative capacity after demyelinating injuries (46). Similarly, here soft substrates enhanced regeneration-associated machinery. To modify the non-regenerative wound’s extracellular environment to resemble the blastema’s, we used HAPLN1 to promote HA deposition, reduce scarring, and enhance skeletal repair. Given that link proteins stabilize aggregates of HA and proteoglycans (30, 47), we propose that HAPLN1 protects HA against fragmentation, which is commonly associated with inflammation and fibrosis (48, 49). Thus, higher-order structural organization of HA matrix is an important player in tissue mechanics and biological function. Previous studies have emphasized biochemical cues or nutritional supplements (50–52) in tissue regeneration. Our data show that modulating the physical microenvironment, through HA stability and tissue mechanics, can also improve regenerative outcomes. Thus, combining physical and biochemical interventions may be an effective approach to promote tissue regeneration in mammals.

The materials and methods are available in the supplementary materials

## Supplementary Material

Figues 1-10

## Data Availability

All raw scRNA-seq expression matrices generated from this project are available in the NCBI Gene Expression Omnibus under the accession number GSE274858. Datasets include single-cell transcriptomic profiles of non-regenerative digits after amputation, with or without inhibition of hyaluronic acid. This study also analyzed publicly available datasets which are described in the supplementary materials section. Code is available at Github (https://github.com/JosephJYW/HA_TissueMechanics_Science2026) and has been deposited on Zenodo (https://doi.org/10.5281/zenodo.18458384) (71). All other data are in the main paper or supplementary materials. The lentiviral vector used to overexpress HAPLN1 in our study, pLV[Exp]-EF1A-mHapln1-mCherry and the scrambled control vector, pLV[Exp]-EF1A-Scramble-mCherry, was constructed and packaged by VectorBuilder. The vector ID is VB230522-1759vcu and VB010000-9390nka and are available upon request.
