# Supplementary material for: Hyaluronic acid and tissue mechanics orchestrate mammalian digit tip regeneration
Source: Science. Author manuscript; Available in PMC 2026 May 5. (PMC7619039; doi:10.1126/science.ady3136)
Supplement: Figues 1-10 [file EMS213339-supplement-Figues_1_10.pdf]

Science
AAAS

Supplementary Materials for

Title: Hyaluronic acid and tissue mechanics orchestrate mammalian regeneration

Authors: Byron W.H. Mui[1,2,3,4], Joseph J.Y. Wong[1]†, Camille E. Dumas[1]†, Jia Hua Wang[1], Toni Bray[1], Kentaro Hirose[1], Lauren Connolly[1], Alexander Winkel[5], Sebastian Timmler[1], Nicholas A. Bright[8], Evelina Sliauteryte[1], Ragnhildur Thóra Káradóttir[1,10], Pamela G. Robey[2], Kristian Franze[5,6,7], Kevin J. Chalut[1,9]†*, and Mekayla A. Storer[1,5]†*

*Corresponding authors. Emails: kc370@cam.ac.uk; ms2786@cam.ac.uk

The PDF file includes:

Materials and Methods
Figs. S1 to S10
Tables S1 to S7

Materials and Methods

Mice

C57BL/6, NOD SCID (6-8 weeks old) and CD1 (postnatal day 2) mice (strain codes: 027, 394, and 022, respectively) were obtained from Charles River Laboratories. The animals were maintained in a standard facility for rodents at the University of Cambridge. They were housed in groups of up to five per cage with controlled temperature, humidity, and 12 h light/dark cycles. Enrichment items provided in the cages included red tubes, shredded paper, and a loft. Mice were fed autoclaved maintenance diet (SAFE R105) and water *ad libitum*. Animals were randomly divided into control and treatment groups, and both male and female mice were used, and data from both sexes were pooled for final analyses. Strategies were implemented to minimize potential confounders including randomizing the order of treatments, cage location and researchers handling the animals. This research was regulated under the Animals (Scientific Procedures) Act 1986 Amendment Regulations 2012 following ethical review by the University of Cambridge Animal Welfare and Ethical Review Body (AWERB). All procedures were conducted strictly following the relevant protocols defined in Home Office Project License PP2274970 and reported according to the ARRIVE guidelines.

Digit amputation model

Digit amputations were performed on 6- to 8-week-old mice as described previously (6, 65). Briefly, mice were orally administered meloxicam (0.5 mg/ml) for analgesia and anesthetized with isoflurane. Then, using sterile scalpels, the distal one-third of terminal phalanges 2, 3, and 4 of the hindlimb were amputated to induce a regenerative response. For non-regenerative amputations, the distal one-third of second phalanges were amputated.

Second harmonic generation microscopy and collagen fiber analysis

Second harmonic generation (SHG) microscopy with the Zeiss LSM 880 NLO and excitation wavelength of 920 nm was used to visualize collagen fibers in post-amputated digits. The collagen fibers were segmented in batch using CT-FIRE (66). Briefly, curvelet transform (CT) denoises the SHG images and enhances the edges of collagen fibres. A fibre extraction (FIRE) algorithm segments collagen fibres by finding fibre centres through a distance transform and ridge detection, linking nearby fragments to construct full, branching fibres. Then, the width, length, number and angle of these segmented fibres were quantified. To quantify the number of fibers oriented around 30°, a custom R script was written. The most common fiber angle for a sample was designated as 0°, around which all other fiber angles were oriented. The total number of fibers oriented ± 30° were quantified and averaged for analysis between conditions. Two sections per digit were analyzed, and these counts were averaged and considered as a single biological replicate. A minimum of three separate animals were analyzed per condition, with each digit sourced from a separate animal. For experiments involving direct injections, each digit was considered a biological replicate. For collagen analysis in hydrogel experiments, a minimum of three independent experiments were performed, and each hydrogel was imaged at 5 different locations, which were averaged as a single biological replicate. For collagen analysis in Hapln1^{OE} experiments, three digits per condition were randomly selected.

Transmission Electron Microscopy

Digits were fixed in 2% Paraformaldehyde (PFA) and 2.5% glutaraldehyde in 0.1M Cacodylate buffer (CaCo), pH = 7.2 at 37°C for 1 hour and stored in fixative at 4°C. Vibratome sections (300

μm) of the fixed digits were washed twice in 0.1M CaCo for 30 minutes, post-fixed with 1% OsO_4 in 0.1M CaCo for 1h at 4°C and washed twice in 0.05 M sodium maleate buffer (SoMa), pH = 5.2 for 30 minutes. Samples were en bloc stained with 0.5% uranyl acetate in 0.05 M SoMa for 1 hour at 4°C and again washed twice with 0.05 M SoMa for 30 min. Samples were dehydrated in increasing ethanol concentrations (50%, 70%, 90%, 2x 100%, 10 minutes each) and infused with propylene oxide (2x 20 minutes), 50/50 mix of epoxy resin (TAAB, overnight) and 100% resin (2 days). Samples were positioned in flat bottom beem capsules and cured for 48 hours at 60°C. Ultrathin sections were cut at 60 nm (Leica EM UC7 ultramicrotome, DiATOME Ultra 35° knife), transferred to formvar coated copper grids and post-stained for 1 minutes with UA-Zero (Agar Scientific) and 30 seconds with lead citrate. Collagen rich regions were identified in 3x3 overview scans at 700x and then 5-7 images per digit were taken (Hitachi HT7800 TEM, 80 kV, 6000x magnification, EMSIS Xarosa camera, 5120x3840 pix, 2.4833 nm/pix). To quantify collagen distribution, images were opened in FIJI/ImageJ, collagen rich ROIs were manually labelled with a touch-screen marker pen (Wacom) and categorized by fiber orientation.

Single-cell library preparation, sequencing, and alignment

For single-cell RNA sequencing (scRNA-seq), tissues from 14 DPA non-regenerative (n = 2, 4 mice pooled per replicate), 4-methylumbelliferone (4-MU) control (n = 2, 5 mice pooled per replicate), and 4-MU treated (n = 2; 5 mice pooled per replicate) digits were freshly dissected, and cells were isolated from the wounded regions. The cells were resuspended at 372 cells/μl in 2% FBS in PBS. Libraries were prepared by using Chromium Next GEM Single Cell 3'mRNA v3.1 (10X Genomics, PN-120233) as per the company's protocol and sequenced on a NovaSeq 6000 at the Cancer Research UK Institute (CRUK, Cambridge, UK). Raw 10X FASTQ files were aligned and quantified using the Cell Ranger Single-Cell Software Suite (v8.0.0, 10X Genomics). The mouse reference used was the mm10 reference genome refdata-gex-mm10-2020A, available at:

https://cf.10xgenomics.com/supp/cell-exp/refdata-gex-mm10-2020-A.tar.gz.

All raw expression matrices generated are available in the Gene Expression Omnibus (GEO) under accession number GSE274858.

Single-cell transcriptomics data analysis

The 10X Genomics scRNA-seq datasets were processed in Python v3.10 using Scanpy v1.9.6 (67). Publicly available blastema (n = 3) and non-regenerative datasets (n = 2) were acquired from Gene Expression Omnibus (GEO) under the accession numbers GSE135985 (6) and GSE143888 (5). Analyses of other regenerative models were performed by acquiring datasets from GSE182141 (68), GSE130699 (69), and GSE234451 (70). Datasets that contained a high degree of cell-free (ambient) RNA molecules were cleaned with SoupX v1.6.2 (71) or CellBender v0.3.2 (72) before downstream pre-processing. Quality control for each dataset involved filtering based on fixed and batch-specific filtering thresholds. Fixed filtering thresholds included removing genes expressed in fewer than 3 cells and cells in which over 10% of the unique molecular identifiers arose from the mitochondrial genome. Batch-specific thresholds included removing cells beyond the upper and lower boundaries for number of genes and number of counts per cell. Doublets were identified using Scrublet v0.2.3 (73), with a threshold doublet score set at 0.2 to 0.25 or DoubletFinder (74). The details of the filtering paraments are listed in Table S1.

During batch pre-processing, size factors for each batch were calculated for data normalization using the computeSumFactors() in R v.4.3. The size factor normalized data were then subjected to

logarithmization (scanpy.pp.log1p), and highly variable genes were detected (scanpy.pp.highly_variable_genes). Principal component analysis was performed by scanpy.pp.pca and the first 30 principal components were used to calculate neighbors (scanpy.pp.neighbors) and perform UMAP (scanpy.tl.umap) constructions. Leiden graph-based clustering (scanpy.tl.leiden) at default settings was used for broad cell type classification with manual annotation. For integration, the top 2,000 highly variable genes were subsetted from the concatenated datasets. scVI v1.0.4 (75) was used to integrate the datasets, with parameters set to n_latent = 30, n_layers = 2, and gene likelihood = "nb". Training was conducted for a maximum of 800 epochs or stopped early based on elbo_validation. The latent representation obtained from the model was used for computing neighbors. The final clustering of cell populations was performed by selecting the most conservative resolution that 1) yielded distinct populations and appropriate heterogeneity based on the UMAPs and 2) retained high and differential expression of canonical cell type-specific markers. *Pdgfrα*-expressing clusters were subsetted from the global integrated datasets for further analyses, and PCAs and UMAPs were re-calculated using scVI modeling and Leiden clustering to reveal sub-populations. The clustering resolution was determined as described above. Cell types were defined by finding marker genes with Scanpy (scanpy.tl.rank_genes_groups) using the Wilcoxon method and corrected by the Benjamini-Hochberg method.

Cell type proportion analyses were conducted with Propellor (Speckle v1.2.0) (76), and $p < 0.05$ designated statistically significant differences between two groups. To account for technical variability across datasets, differential gene expression was performed using MAST v.1.8.2 (77). MAST analyses were used to compare the Fibroblast 1 and 2 clusters against all other cell types, as well as against OLs. For the 4-MU experiment, MAST analysis was performed on all *Pdgfrα*-expressing clusters between control and 4-MU datasets. Differentially expressed genes (DEGs) were determined as log2fold-change ≥ 0.5 and q-value < 0.05. The resulting DEGs were input for ClusterProfiler v4.10.1 (78) for gene ontology functional enrichment analysis. For complete Matrisome and collagen and proteoglycan scoring, ECM-related genes were taken from *mus musculus* complete matrisome list from the Matrisome Project (44), and gene list scores were calculated using sc.tl.score_genes. Table S2 lists the genes comprising each module.

<u>Tissue preparation, immunostaining, and microscopy</u>

Mouse tissues were harvested and fixed with 4% paraformaldehyde at 4 °C overnight and decalcified using 0.5 M pH 7.2 for 14 days. Tissues were cryo-protected overnight in 30% sucrose, embedded in OCT, and rapidly frozen. For deep imaging of digits, samples were processed as previously described (79). Briefly, tissues were sectioned to the mid-sagittal plane and blocked overnight in PBS with 5% bovine serum albumin (BSA), 10% dimethyl sulfoxide, and 0.5% Triton X-100 in PBS. Primary antibodies were diluted in blocking buffer and incubated with samples for 3 days. Samples were washed twice in PBS with 0.1% Triton X-100 (PBS-T) for 1 h per wash, and the third wash was performed overnight. Secondary antibodies were also diluted in blocking buffer, and samples were incubated for 3 days. Samples were washed as before, and Hoechst was used as a counterstain in the final 1 h wash. On the final day, samples were dehydrated and cleared using either benzyl alcohol:benzyl benzoate at a 1:2 ratio for 1 h or 50:50 CUBIC R+(M):PBS overnight at RT (TCI Chemicals, T3741), followed by 100% CUBIC R+M for 1 day. Lastly, samples were mounted on to glass bottom dishes for imaging. For thin-tissue IHC, samples were sectioned sagittally at 14 μm thickness. Prior to staining, sections were heated for 10 min at 37 °C. Subsequently, slides were soaked in PBS with 0.5% Triton X-100 for 30 min and blocked for 1 h

with 5% BSA in PBS-T. If primary antibodies were from goat or sheep hosts, then 10% donkey serum in PBS-T was used. Primary antibodies were diluted in blocking buffer, and slides were incubated with antibodies overnight at 4 °C. The next day, slides were washed 4 times in PBS-T, 5 min each. Appropriate fluorophore-conjugated secondary antibodies were diluted in blocking buffer and applied to samples for 2 h with Hoechst. Slides were washed 4 times in PBS-T and finally mounted in ProLong Gold Antifade Mountant (Thermo Fisher Scientific, P36930). The list of primary antibodies can be found in Table S3.

For visualization of hyaluronic acid (HA) using hyaluronic acid binding protein (HABP, Merck, 385911-50UG), sections were incubated with streptavidin-peroxidase from Streptomyces avidinii (Merck, S5512-.1MG), followed by chromogenic development using the Pierce DAB Substrate Kit, as per the manufacturer's instructions (Thermo Fisher Scientific, 34002). For fluorescence microscopy, streptavidin secondaries were used. For pSMAD1/5/8 immunostaining, antigen retrieval (10 mM sodium citrate dihydrate with 0.1% Triton X-100 at pH 6.0) was performed on cryo-sectioned, formalin-fixed tissues for 2 h at 70 °C in a water bath. All remaining steps were performed using staining procedures described above, except PBS was replaced with TBS. For immunocytochemistry, cells were fixed with 4% paraformaldehyde for 15 min and permeabilized in 0.5% Triton X-100 in PBS for 30 min. Except for pSMAD staining, which required TBS, staining procedures were the same as described above. For actin visualization, phalloidin (Proteintech, PF00001) staining was performed for 30 min. The hydrogels were inverted onto glass bottom dishes for imaging.

To visualize samples, the following confocal microscopes were used: Zeiss LSM 980 Airyscan2 and the Leica Stellaris 8. For image analysis of tissues, two sections per digit were analyzed, and these values were averaged and considered a single biological replicate. A minimum of three separate animals were analyzed per condition, with each digit sourced from a separate animal. For image analysis of cells, a minimum of three independent experiments were performed, and values for at least 10 separate cells at 5 different locations were averaged as a single biological replicate. The ImageJ (80) software was used for all image analysis.

Hyaluronic acid extraction and enzyme-linked immunosorbent assay

To isolate hyaluronic acid (HA) from tissues, non-regenerating wounds and blastemas were micro-dissected, weighed, and incubated with digestion buffer containing 0.1 M Tris, 0.15 M NaCl, 0.01 M $CaCl_2$, 5 mM deferoxamine mesylate, and 0.5 mg/ml Proteinase K (Merck, P6556-5MG) at pH 8.3 for 2 h with vortexing every 30 min. Samples were boiled at 100 °C for 20 min to heat-inactivate Proteinase K and chilled on ice. 1 µl of Benzonase (Merck, E1014-5KU) was added to each sample and incubated for 1 h to degrade DNA and RNA. Then, samples were centrifuged for 15 min at 21,000 g at 4 °C, and the supernatant was collected into a separate tube. An equal volume of phenol:chloroform:isoamyl alcohol (Merck, 77617-100ML) was added, and after vortexing, the samples were centrifuged for 15 min at 14,000 g and 4 °C to separate the aqueous components from the other organics. This step was repeated using pure chloroform to remove residual phenol from the aqueous phase. HA was precipitated using 100% ethanol and spun at 10,000 g for 5 min and washed with an additional volume of ethanol. The samples of extracted HA were resuspended in water. An enzyme-linked immunosorbent assay (ELISA) to quantify the amount of HA in digit tissues was performed according to the manufacturer's instructions (Biotechne, DHYAL0). Digits from four mice were pooled for a single biological replicate, with three replicates per condition.

Atomic force microscopy

Freshly isolated, post-amputated digits were cut mid-sagittally using a sharp scalpel and partially embedded in 4% agarose to stabilize the tissues on glass-bottom dishes. After the agarose had set, the samples were submerged in low-glucose DMEM without phenol red (Thermo Fisher Scientific, 11880036) and maintained at 34 °C. Atomic force microscopy (AFM) indentation measurements were performed using the setup previously described (81). Briefly, a petri dish containing the mounted digits was placed on the stage of an inverted microscope, and an AxioZoom V16 stereomicroscope (Zeiss) was mounted above the setup and used to acquire brightfield images through the AFM head. These images were used to designate a rectangular grid containing 2D spatial information upon which indentation measurements were performed. The exposed tissues were probed with 37.28 µm diameter polystyrene beads glued to Arrow-TL1 cantilevers (nominal spring constant = 0.01 N/m, NanoWorld, Arrow TL1). During measurements, the cantilever was approached at 20 µm/s until an indentation force of 10 nN was reached. This force was maintained for 3 seconds, and the resulting creep response (i.e. deformation over time) recorded.

The reduced apparent elastic modulus $K = \frac{E_0}{(1-v^2)}$ values was extracted by a custom-written MATLAB script fitting the Hertz model to the force-distance data of the initial indentation (81). A custom-written Python script implementing the Power Law Rheology (PLR) model (82) was used to obtain fluidity values. β can have values ranging from 0 (for an elastic solid) to 1 (for a viscous fluid). For AFM experiments, a minimum of 4 separate digits were measured per condition, with each digit sourced from a separate animal.

Depletion of HA matrix in mice

For intermittent degradation of HA, mice that underwent distal third phalanx (P3) amputations were administered 0.4-1 U per digit of hyaluronidase from bovine testes (Merck, H3506-100MG) reconstituted in 0.1% BSA. Control mice received 0.1% BSA alone. Administration of substances was performed at 8, 10, and 12 DPA using a 33G Hamilton syringes (Hamilton Company, 7635-01). Digits were harvested at 14DPA for processing and further analyses. Each digit was considered a biological replicate, with three replicates per condition.

For continuous knockdown of HA, 4-MU was incorporated into chocolate-flavored chow (ssniff Spezialdiäten) at 50 g/kg, resulting in a daily uptake of 250 mg of 4-MU per mouse. Control mice received the same chocolate-flavored chow without 4-MU. To acclimatize the mice to the modified diet and prevent weight loss, they were first fed maintenance chow:modified diet at a 50:50 ratio for the first seven days. Then, mice were maintained on the modified diet until the experimental endpoint. After acclimatization, mice underwent regenerative amputations, and tissues were collected at 14 and 28 DPA for further analyses. Each mouse was considered a biological replicate, with at least three replicates per condition.

Quantification of digit length and area

Fixed digits were imaged on a Leica stereomicroscope, and images were analyzed in ImageJ, where the region of the nail was manually outlined using the Polygon Selection tool, and the nail length was traced using the Straight Line tool. For the 4-MU experiment, each mouse was considered a biological replicate, and for experiments involving direct injections of substances, each digit was considered a biological replicate.

Microcomputed tomography

To assess the skeletal morphology of mouse digits, 4% paraformaldehyde-fixed samples were scanned at either 14 or 28 DPA using a Nikon XTEK 225 Micro CT Scanner. Scan settings were the following: 2.9-4.9 µm resolution, energy at 75-90 kV and 30-48 µA, no filtration, 708-1000 ms exposure, 1080 projections, and two frames per projection. Image processing was done using CT Pro 3D and CT Agent (Nikon). The Materialise Mimics software was used to model samples in 3D and calculate volume and dimension measurements. For the 4-MU experiment, each mouse was considered a biological replicate, and for experiments involving direct injections of substances, each digit was considered a biological replicate. All experiments contained at least three biological replicates per condition.

Digit cell and dermal fibroblast isolation and culture

To isolate uninjured or post-amputated P3 cells, the nails from 6-8 week old C57BL/6 mouse digits of both sexes were first removed to expose the underlying P3 bone and surrounding tissues. These tissues were digested in 0.25 mg/ml Liberase TH (Scientific Laboratory Supplies, 5401135001) in PBS for 1 h at 37 °C. The dissociated cells were treated with 20 U/ml DNase I (Merck, 4536282001) for 5 min at 37 °C. Isolation of P2 cells was performed similarly as above. Briefly, P2 tissues were separated from P3 at the distal interphalangeal joint, and the skin was peeled from the P2 bone and minced. Together, the skin and the bone were incubated in cell dissociation buffer for 1 h at 37 °C. Cells from both sexes were pooled since we did not observe differences in digit wound healing between males and females. They were maintained in standard fibroblast expansion medium, which contained 10% fetal bovine serum (FBS, Gibco, 10270106), 1% Penicillin/Streptomycin (Merck, P0781), and 10 ng/ml fibroblast growth factor 2 (FGF-2) (Peprotech, 100-18B) in low-glucose DMEM with pyruvate and HEPES (Thermo Fisher Scientific, 12320032). Cells were seeded at 25,000 cells/cm2 and expanded in 20% O2 and 5% CO2 in a 37 °C humidified incubator.

To isolate dermal fibroblasts, the back skin of CD1 mice at postnatal day 2 of both sexes were dissected, and the tissues were placed in 0.25% trypsin overnight at 4 °C. The next day, the dermis was separated from the epidermis using forceps and minced with scissors. The minced tissues were incubated in 1 mg/ml Collagenase P (Merck, 11213857001) and 2 mg/ml Dispase II (Merck, D4693-1G) and reconstituted in DMEM containing 2% FBS for 1 h at 37 °C. DNA was digested using 20 U/ml DNase I for 5 min at 37 °C. Fibroblast expansion medium was added to halt the digestion. Cells were spun for 10 min at 300 x g and resuspended in fibroblast expansion medium.

Fabrication of polyacrylamide gels of different stiffnesses

StemBond hydrogels were fabricated as previously described (50). Briefly, support coverslips were treated with 0.2 M sodium hydroxide for 35 min, cleaned, and functionalized with 3-(Trimethoxysilyl)propyl methacrylate (Merck, M6514-25ML) for 2 h. Top coverslips were treated with dichlorodimethylsilane (Merck, 440272-100ML) for 5 min. Hydrogel solutions were prepared according to Table S3 with the inclusion of 6-acrylamidohexanoic acid (Tokyo Chemical Industry, A1896) and degassed in a vacuum chamber for 20 min. To polymerize the hydrogels, TEMED (Merck, T22500-5ML) and APS (Merck, A3678-25G) were added, and the hydrogels were sandwiched between the support and top coverslips for 35 mins. The hydrogels were hydrated overnight in 1% Penicillin-Streptomycin in PBS. The next day, top coverslips were detached from the hydrogels under sterile conditions and equilibrated with MES buffer (0.1 M MES hydrate (Merck, M2933-25G), 0.1 M NaCl, pH 6.1). Hydrogels were activated by a 30 min treatment with

0.2 M EDAC (Scientific Laboratory Supplies, 03450-25G) and 0.5 M NHS (Thermo Fisher Scientific, 157272500) in MES buffer with constant rocking. After, hydrogels were coated with poly-d-lysine hydrobromide (Merck, P0899-10MG) at 100 μg/ml reconstituted in 0.05 M HEPES (Merck H3375-25G) pH 8.5. Coating was performed on a rocker for 2 h. Coating solution was aspirated, and the hydrogels were blocked with 0.5 M ethanolamine.

To test how substrate stiffness influences cell behavior, cells were cultured on stiff (50 kPa) or soft (0.7 kPa) hydrogels to mimic the mechanical microenvironment of the blastema and fibrotic tissue, respectively. Cells of no more than passage 4 were attached to the hydrogels for 24 h before treatment with PDGF-BB (50 ng/ml, Peprotech, 100-14B) or BMP-7 (200 ng/ml, Peprotech, 120-03P) in DMEM containing 1% FBS for experiments lasting less than or equal to 24 h. For longer experiments, DMEM with 10% FBS was used to maintain cell viability. To induce collagen synthesis and fibrillogenesis, 25 μg/ml 2-phospho-L-ascorbic acid trisodium salt (Merck, 49752-10G) and/or TGF-β1 was supplemented into the medium and replenished every other day until the experimental endpoint. For all hydrogel experiments, at least 3 independent experiments were performed.

Reverse transcription quantitative polymerase chain reaction

To measure gene expression levels, cells were first lysed by direct addition of TRIzol (Thermo Fisher Scientific, 15596026) for 5 min. Further RNA processing and purification was performed using Direct-zol RNA Microprep Kits (Zymo Research, R2060) according to the manufacturer's instructions. Briefly, an equal volume of 100% ethanol to TRIzol was added to samples and spun in columns for two rounds of washing before elution in DNase/RNase-free water. Next, samples were treated with RQ1 RNase-free DNase (Promega, M6101) for 30 min at 37 °C, followed by termination of the reaction using the supplied Stop Solution for 10 min at 65 °C. RNA quality and concentration was measured using a Nanodrop Spectrophotometer (Thermo Fisher Scientific). Complementary DNA (cDNA) synthesis was carried out by first heating the RNA-primer mix containing 2.5 μM random hexamers (Thermo Fisher Scientific, N8080127), 0.5 mM dNTP (Thermo Fisher Scientific, 18427013), and 400 ng total RNA for 5 min at 65 °C and chilling on ice for 1 min. Next, a reverse transcriptase mix containing 1x SuperScript IV, 5 mM DTT (Thermo Fisher Scientific, 18090010), and 2.0 U/μl RNaseOUT RNase Inhibitor (Thermo Fisher Scientific, 10777019) was added to each tube, which was incubated at 23 °C for 10 min, 55 °C for 10 min, and finally 80 °C for 10 min. cDNA was diluted 1:200, and each PCR reaction contained 2 ng of cDNA, PowerTrack SYBR Green Master Mix (Thermo Fisher Scientific, A46109), and 800 nM of primer pairs. Reactions were carried out using the QuantStudio Real-Time PCR machine and software (Thermo Fisher Scientific, Waltham, MA). For all qPCR experiments, at least three independent experiments were performed. Statistical analyses were performed on $-\Delta\Delta CT$ values, but expression graphs were depicted as fold change ($2^{-\Delta\Delta CT}$). A list of the primers can be found in Table S4.

Western blotting

For immunoblotting, adherent cells on hydrogels were inverted onto Parafilm with RIPA buffer (Thermo Fisher Scientific, 89900) containing Halt Protease and Phosphatase Inhibitor Cocktail (Thermo Fisher Scientific, 78440). After 5 min, the cell lysate was collected into a microcentrifuge tube and agitated for 30 min at 4 °C. Then, the lysate was centrifuged for 5 min at 14,000 x g and 4 °C. The supernatant was aspirated, and the total protein was quantified using the BCA method (Thermo Fisher Scientific, 23225). Samples were boiled in Laemmli sample buffer (5x, 250 mM

Tris base, 5% SDS), 50% glycerol, and 0.1% bromophenol blue with 2.5% 2-mercaptoethanol at 100 °C for 5 min, and 10 µg of total protein per lane, along with a Precision Plus Protein Kaleidoscope Prestained Protein Standards ladder (Bio-Rad Laboratories, 1610375), was separated by gel electrophoresis. Transfer of proteins to PVDF membranes was performed using the tank transfer method with Towbin buffer (10x, 0.25 M Tris base, 1.92 M glycine). The membranes were blocked with 5% BSA in TBS with 0.1% Tween-20 (TBS-T) for 1 h with constant agitation, followed by immunostaining with primary antibodies in blocking buffer overnight at 4 °C. Six 5-min washes with TBS-T were performed, followed by application of secondary antibodies for 1 h in blocking buffer. After another six 5-min TBS-T washes, protein was detected using SuperSignal West Pico PLUS Chemiluminescent Substrate (Thermo Fisher Scientific, 34577) according to the manufacturer's instructions. Visualization was performed using the G:Box chemi XRQ machine (Syngene). Quantitative analysis of the immunoblots were performed using ImageJ. Densitometry analysis was performed by calculating background-subtracted integrated densities of pSMAD1/5/8 or HAPLN1 normalized to the loading control GAPDH (Abcam, ab9485, 1:10,000). For all western blots, at least three independent experiments were performed.

Overexpression of *Hapln1* in dermal fibroblasts

pLV[Exp]-EF1A-mHapln1-mCherry; VB230522-1759vcu, (Hapln1^{OE}) and pLV[Exp]-EF1A-Scramble-mCherry; VB010000-9390nka, (mCherry control) transfer plasmids were cloned and transformed in *Escherichia coli* (VB UltraStable™ Chemically Competent Cells #UC001-010) by VectorBuilder (VectorBuilder, Chicago, IL). 3rd generation lentivirus plasmids pMDLg/pRRE, pmD2.G, and pRSV-Rev (Addgene plasmids #12251, #12259, and #12253, respectively) were used to generate lentivirus containing the Hapln1^{OE} or mCherry Control transfer plasmid. HEK293T cells (ATCC, 293T-CRL-3216) of passage less than 20 were seeded at a density of $4x10^6$ cells per 10-cm dish in high-glucose DMEM containing HEPES (Thermo Fisher Scientific, 10564011) and 10% FBS and allowed to attach overnight. The next day, fresh medium was supplied to the cells, which were subsequently transfected with the transfer, envelope, and packaging plasmids at a ratio of 4:2:1:1 by size (transfer:pMD2.G:pMDLg/pRRE:pRSV-Rev). The transfectant reagent:DNA complexes were made by combining *Trans*IT-VirusGEN (Mirus Bio, MIR 6704) with plasmids in Opti-MEM I (Thermo Fisher Scientific, 31985062) and incubating for 30 mins to form complexes. The *Trans*IT-VirusGEN:DNA complexes were added drop-wise to different areas of the dish. Lentivirus was collected at 48 and 72 h post-transfection and passed through a 0.45 µm filter. The lentivirus was then incubated 2:1 with Lenti-X™ Concentrator (Takara, 631231) for 1h and concentrated by centrifugation at 1500 g for 45min at 4 °C. The supernatant was discarded, and the virus was resuspended in 100 µl PBS per 10-cm dish.

Lentivirus multiplicity of infection and titer calculation was performed using a dilution series. The day prior to transduction, back skin dermal fibroblasts were plated at a density of 15,000 cells/cm^2 in a 24-well plate. A serial dilution of lentivirus was added to the wells with the addition of 4 µg/ml of hexadimethrine bromide (Merck, H9268-10G). The plate was spun at 1,000 g for 2 h at 32 °C, after which the medium was replaced with fresh fibroblast expansion medium, whose components were described above. After 48 h, the percentage of reporter-positive cells was calculated. The volume of virus needed to achieve at least 95% infection efficiency was used to transduce fibroblasts for all experiments using the steps described above.

Flow cytometry analysis

To confirm lentivirus transduction efficiency, infected cells were harvested and passed through a 40 µm filter. Then, cells were incubated with ready-to-use DAPI (Miltenyi Biotec, 130-111-570) for 5 min in 2% FBS and 2.5 mM EDTA in PBS. Unstained fibroblasts, fibroblasts stained with DAPI, and mCherry-positive cells were used as single-stained controls for compensation and gating. Typically, 100,000 events were recorded for each sample. Data were acquired on a BD Fortessa flow cytometer, and data analysis was performed in the FlowJo v10.1 software. Three independent experiments were performed for quantification of transduction efficiency.

Transplantation of genetically modified dermal fibroblasts into mice

Genetically modified fibroblasts were expanded, and cells of passage no later than 6 were used for transplantation. Briefly, detached cells were washed in PBS and resuspended at a concentration of 150,000 cells/µl in 33% EncapGel (Merck, 922412-1EA). NOD SCID mice, having undergone non-regenerative amputations, were administered 1 µl of cells or vehicle in the non-regenerative stump using a 32G Neuros Syringe (Hamilton, 65458-01) at 6 and 12 DPA. Mice digits were harvested at 14 and 28 DPA for further processing. Each digit was considered a biological replicate, with 18 digits per condition.

Injection of lentivirus into the mouse digits

$Hapln1^{OE}$ and mCherry control lentiviruses were generated as described above. A volume of 1µl of the $Hapln1^{OE}$ or mCherry Control lentiviruses (5×10^6 IU/mL) was directly injected into the P2 digits of C57BL/6 mice using the Microliter Neuros Syringe (Hamilton, 65458-01). One week after the injection, the digits were amputated at the non-regenerative P2 level, and an additional injection into the stump was performed at 6 DPA. Mice digits were harvested at 14 and 28 DPA for further processing.

Statistical analysis

Statistical analysis was performed using GraphPad Prism 10. The Shapiro-Wilk test was used to assess normality, and outliers were identified by ROUT tests (for multiple outliers, Q = 1%). A two-tailed student's *t*-test was used for pairwise comparisons, and a two-way or three-way ANOVA followed by Tukey's test was used for multiple comparisons. Data were presented as mean $\pm$ SEM. $p<0.05$ was considered statistically significant. For AFM data, non-parametric Mann-Whitney test was used for statistical analysis and were shown as box-and-whisker plots. For all the figures, the number of mice and independent experiments are indicated in the figure legend. For mouse studies, sample size was determined by POWER calculations. Data were analyzed in a blinded manner, and wherever possible, investigators involved in treatment administration were not involved in data analyses or outcome assessment.

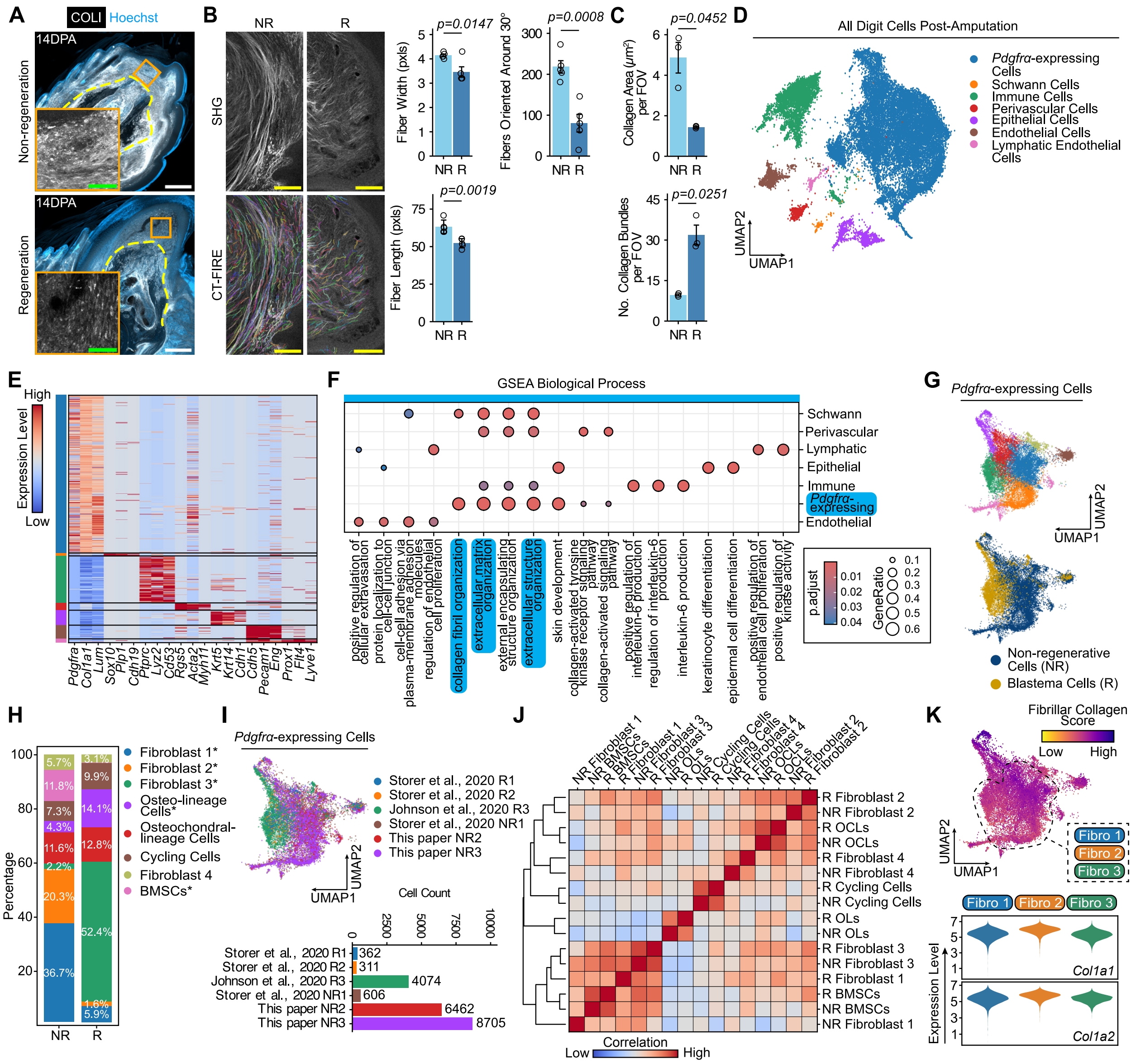

Fig. S1. Cellular and ECM dynamics during digit wound healing.

(**A**) Immunofluorescence showing COLI (white) in non-regenerative and regenerative digits 14 days post-amputation (DPA). Dashed line, border of the phalanx bone. Scale bars, 250 µm and 50 µm in magnified views. (**B**) Left, second harmonic generation (SHG) microscopy showing collagen fibers (white) and their segmentation using CT-FIRE in the non-regenerative wound (NR) and regenerative blastema (R) 14 DPA. Scale bars, 100 µm. Right, quantification of collagen fiber width, length, and orientation, related to Fig. 1A. n = 5 mice per condition. (**C**) Top, surface area occupied by collagen fibers per field of view (FOV). Bottom, number of collagen fibers bundles per FOV, related to Fig. 1B. n = 3 mice per condition. (**D** and **E**) UMAP of the transcriptional identities of seven major cell types in the wounded digit (integrated non-regenerative and blastema datasets) in D. Heat map of top differentially expressed genes in E. (**F**) Upregulated gene ontology terms for each population. (**G** and **H**) UMAP of seven distinct transcriptional signatures among *Pdgfrα*-expressing cells, and cells split by NR and R datasets in G. Cell proportions of *Pdgfrα*-expressing sub-types in H. The asterisk (*p < 0.05) denotes significant differences in proportion

between wound healing conditions. n = 3 non-regenerative datasets (at least 4 mice pooled per dataset), n = 3 blastema datasets (at least 2 mice pooled per dataset). **(I)** Top, *Pdgfrα*-expressing cells color-coded by their dataset of origin. Bottom, bar plot of cell counts for each dataset. (**J**) Correlation matrix comparing all *Pdgfrα*-expressing sub-types by NR and R conditions. (**K**) Top, UMAP showing cell scoring of fibrillar collagen gene expression. Bottom, violin plots of *Col1a1* and *Col1a2* expression among Fibroblast 1 (Fibro 1), Fibroblast 2 (Fibro 2), and Fibroblast 3 (Fibro 3) cells. Statistical significance was determined by two-tailed unpaired student's *t*-test (B and C) or moderated *t*-tests in an empirical Bayes framework (H). Additional details on statistics and reproducibility are in the Materials and Methods.

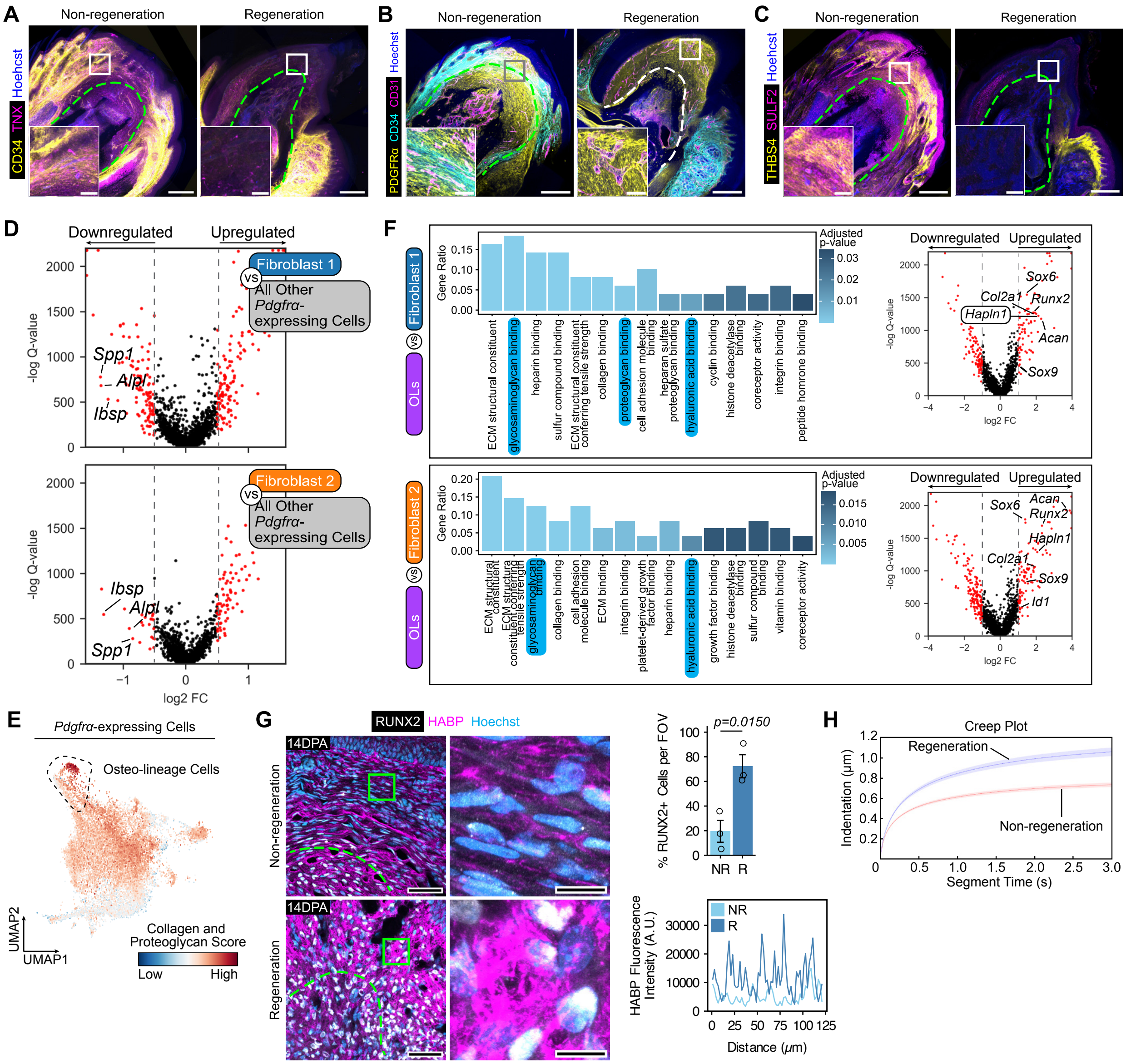

Fig. S2. The blastema is enriched with osteo-lineage cells and hyaluronic acid.
(**A-C**) Immunofluorescence showing CD34 (yellow) and TNX (magenta) in A; PDGFR*α* (yellow), CD34 (cyan), and CD31 (magenta) in B; and THBS4 (yellow) and SULF2 (magenta) in C in digits 14 DPA. Dashed line, border of the phalanx bone. Scale bars, 250 µm and 50 µm in magnified views. (**D**) Volcano plots of differentially expressed genes in Fibroblast 1 or Fibroblast 2 cells compared to *Pdgfra*-expressing cells. (**E**) UMAP of cells scored by their expression of collagen and proteoglycan genes. (**F**) Left, gene ontology terms upregulated in osteo-lineage cells (OLs) compared to Fibroblast 1 or Fibroblast 2 cells. Right, volcano plot of differentially upregulated genes in osteo-lineage cells (OLs). (**G**) Left, immunofluorescence of RUNX2 (white) and HABP (magenta) in non-regenerative and regenerative digits at 14DPA. Dashed line, border of the phalanx bone. Scale bars, 50 µm and 10 µm in magnified views. Right, quantification of the percentage of RUNX2^{+} cells per field of view (FOV) in the non-regenerative wound (NR) versus regenerative blastema (R). n = 3 mice per condition. Plot of HABP fluorescence intensity across distance. (**H**) Atomic force microscopy creep plot, related to Fig. 1L. Data are mean ± SEM and

are representative of at least three independent experiments. Statistical significance was determined by the two-part generalized linear model MAST with a joint test summing likelihood ratio or Wald test statistics (D and F) or two-tailed unpaired student's *t*-test (G). Additional details on statistics and reproducibility are in the Materials and Methods.

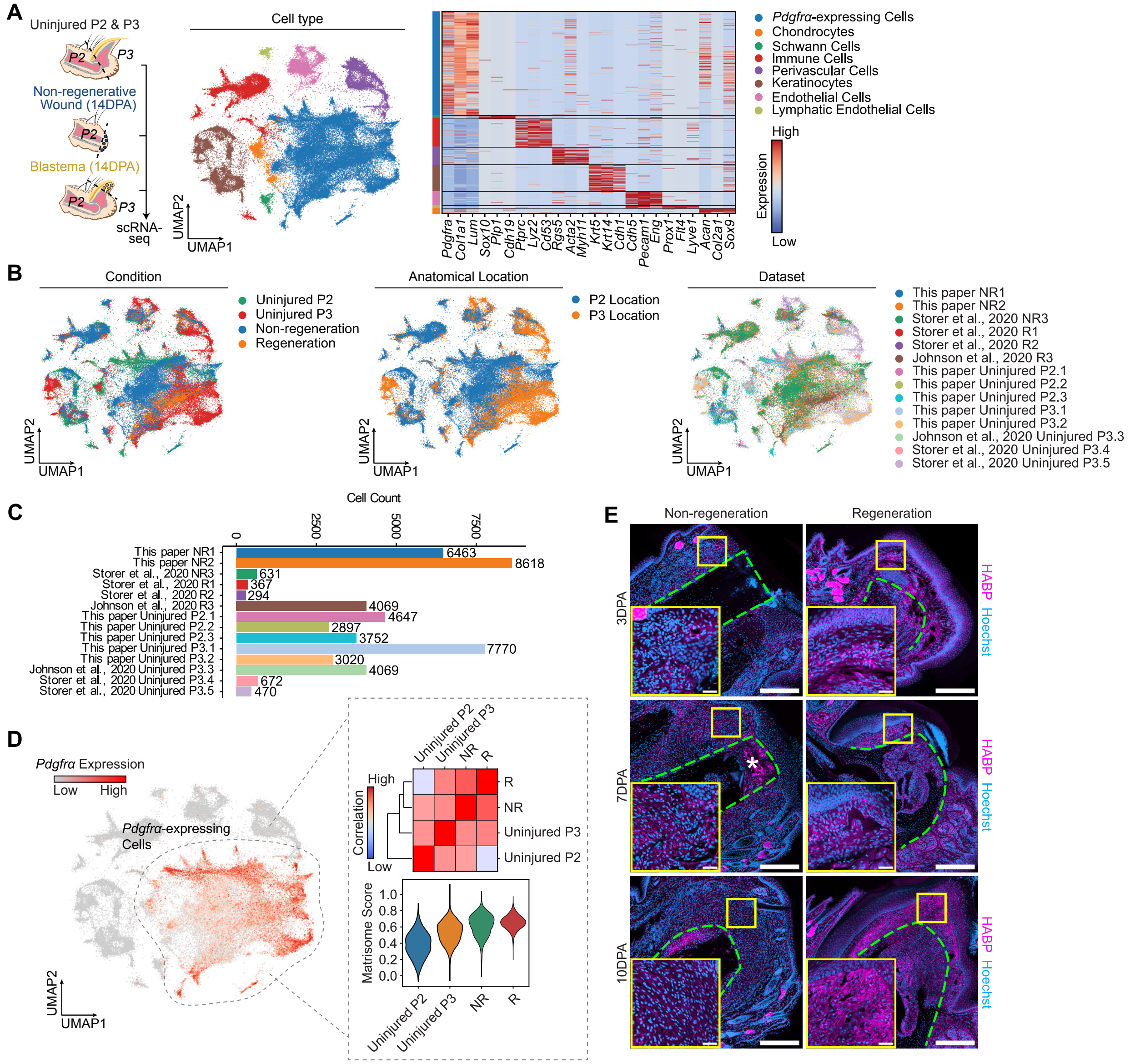

Fig. S3. Intrinsic cellular differences and amputation remodeling shape divergent ECM microenvironments.

(**A**) Left, single-cell RNA sequencing (scRNA-seq) strategy for discerning the role of pre-existing and injury-induced cellular activity on establishing the wound niche 14DPA. P2, second phalanx; P3, third phalanx. Middle, UMAP of the transcriptional identities of eight major cell types. Right, heat map of top differentially expressed genes. (**B**) Left, UMAP of all cells distinguished by both anatomical location and uninjured or injured state. Middle, UMAP of all cells distinguished by P2 or P3 location. Right, UMAP of all cells color-coded by their dataset of origin. (**C**) Bar plot of cell counts for each dataset. (**D**) Left, UMAP highlighting *Pdgfrα*-expressing cells. Right, correlation matrix comparing all *Pdgfrα*-expressing cells by anatomical location and uninjured or injured state. Violin plots of matrisome scores. (**E**) Immunofluorescence of HABP (magenta) in non-regenerative and regenerative digits at 3, 7, and 10 DPA. *Asterisk, bone autofluorescence. Scale bars, 200 μm and 25 μm in magnified views.

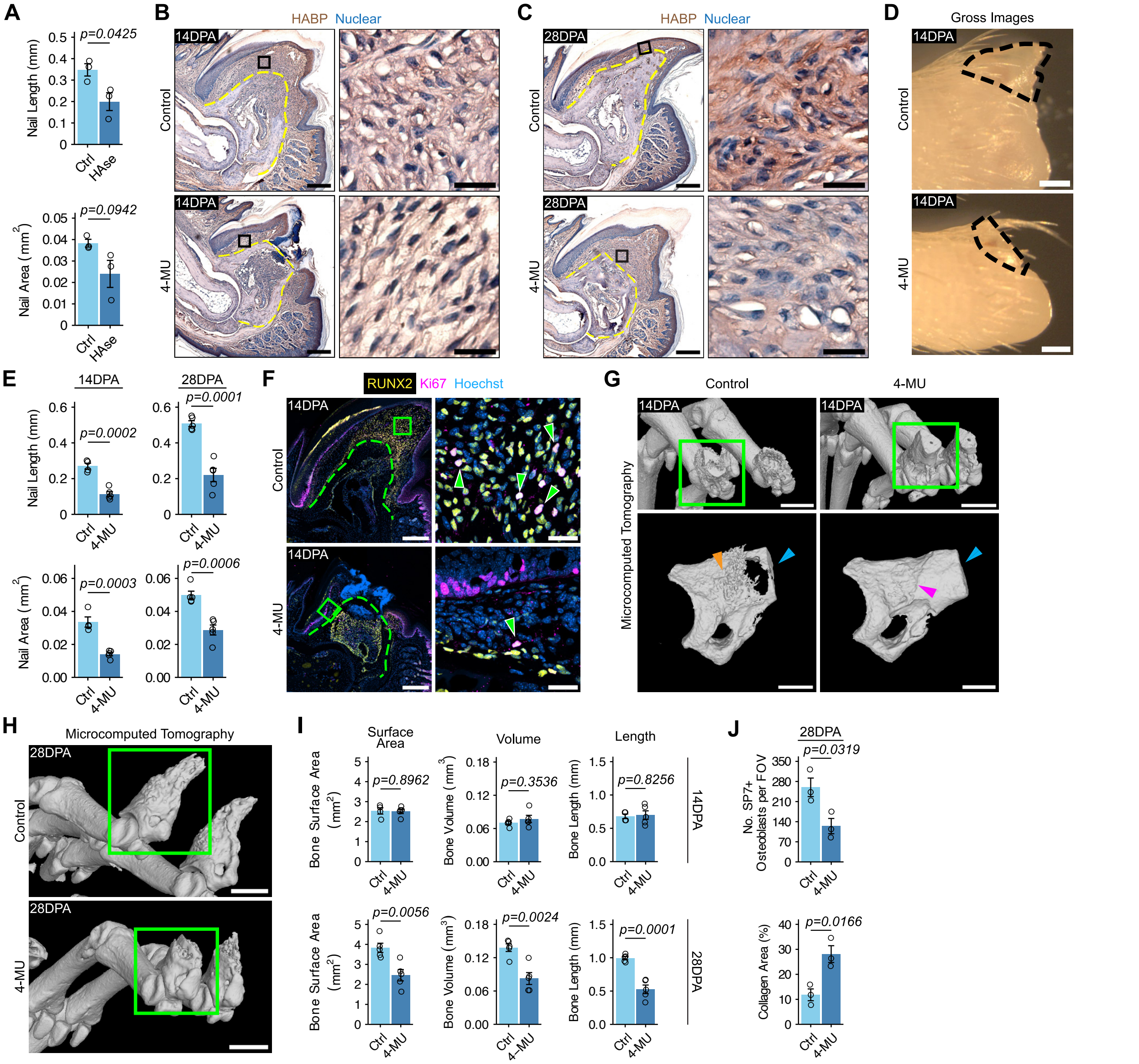

Fig. S4. Hyaluronic acid depletion impairs nail and skeletal repair after distal third phalanx amputations.

(**A**) Quantification of nail length and area at 14 DPA comparing control versus hyaluronidase (HAse)-treated digits, related to Fig. 2A. n = 3 mice per condition. (**B** and **C**) Immunostaining of HABP (brown) in control and 4-MU digits 14 DPA (B) and 28 DPA (C). Dashed line, border of the phalanx bone. Scale bars, 250 μm and 25 μm in magnified views. (**D**) Gross images of control versus 4-MU digits 14 DPA. Dashed line, border of the nail. Scale bars, 100 μm. (**E**) Quantification of nail length and area at 14 DPA and 28 DPA comparing control versus 4-MU digits, related to Fig. 2D. n = 5 mice per condition. (**F**) Immunofluorescence showing RUNX2 (yellow) and Ki67 (magenta) at 14 DPA, related to Fig. 2F. Dashed line, border of the phalanx bone. Arrowheads, double-positive cells. Scale bar, 250 μm and 30 μm in magnified views. (**G** and **H**) Microcomputed tomography analysis of skeletal morphologies of control and 4-MU digits at 14 DPA (G) and 28 DPA (H). Blue arrowhead, site of the original amputation; orange, regions of new bone formation; magenta, cortical bone degradation. Scale bars, 500 μm and 250 μm in magnified views. (**I**)

Quantification of bone surface area, volume, and length of third phalanx bones 14 DPA and 28 DPA in control versus 4-MU digits, related to Fig. 2G. n = 5 mice per condition. (**J**) Top, quantification of the number of $SP7^{+}$ osteoblasts per field of view. Bottom, collagen area in the wounded region of control and 4-MU digits at 28 DPA. n = 3 mice per condition, related to Fig. 2H. Data are mean ± SEM and are representative of at least three independent experiments. Statistical significance was determined by two-tailed unpaired student's *t*-test (A, E, I, and J). Additional details on statistics and reproducibility are in the Materials and Methods.

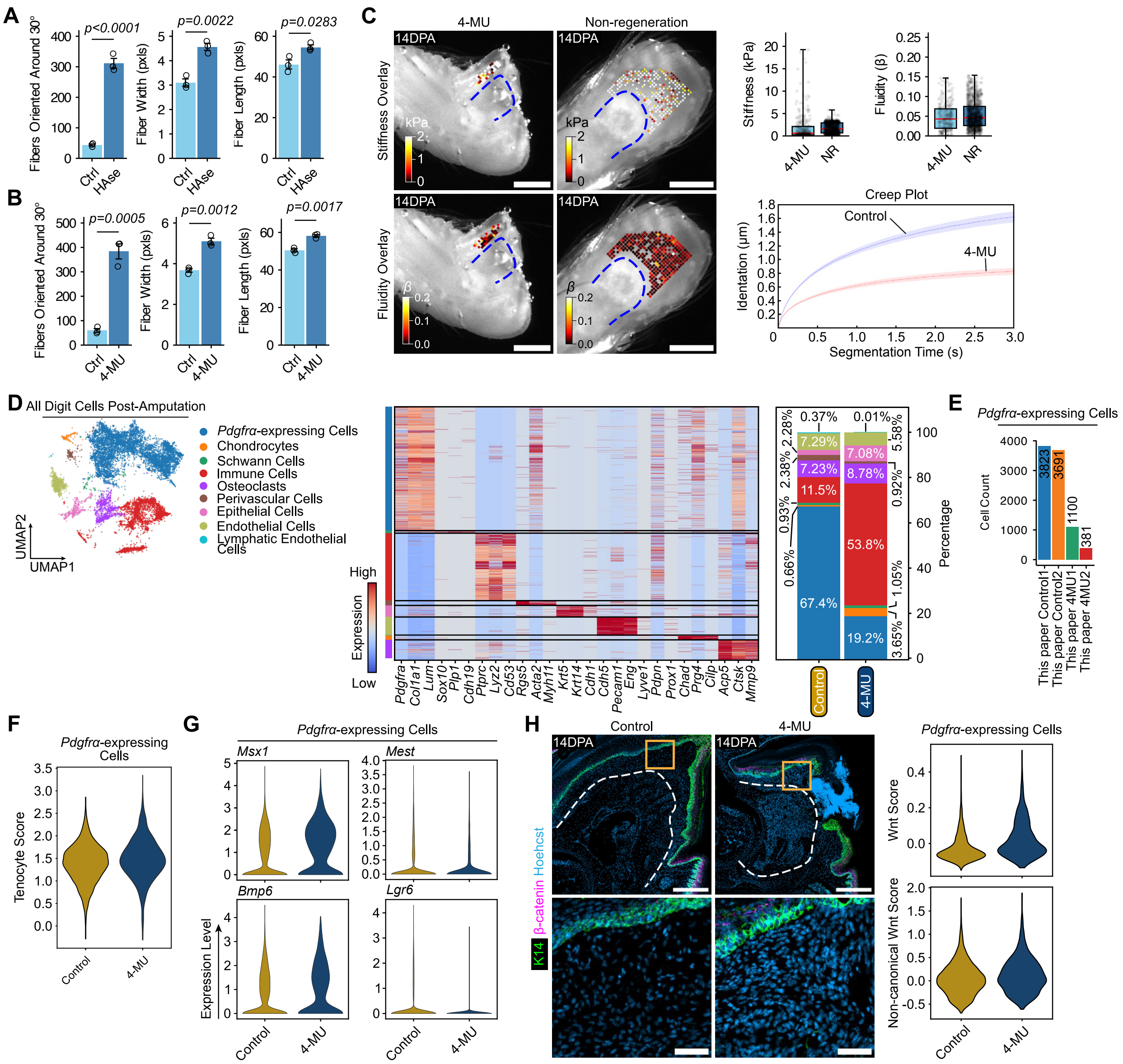

Fig. S5. Hyaluronic acid depletion promotes fibrotic collagen matrices and alters the cellular landscape.

(**A** and **B**) Quantification of the collagen fiber orientation, width, and length after amputations, comparing control versus hyaluronidase (HAse)-treated digits in A and 4-methylumbelliferone (4-MU) digits in B, related to Fig. 3A and B. n = 3 mice per condition. (**C**) Left, atomic force microscopy stiffness and fluidity maps of 4-MU and non-regenerative (NR) digits at 14 days post-amputation (DPA). Dashed line, border of the phalanx bone. Scale bars, 500 μm. Right, quantification of the average stiffness, fluidity, and creep of NR and 4-MU digits, related to Fig. 3C. n = 4 mice per condition. (**D**) Left, UMAP of the transcriptional signatures of nine major cell types in the control and 4-MU digits 14 DPA. Middle, heat map of top differentially expressed genes. Right, proportion analysis of all cell types. (**E**) Bar plot of cell counts per dataset. (**F**) Violin plot of tenocyte scores. (**G**) Violin plots of blastema marker genes. (**H**) Left, immunofluorescence showing K14 (green) and β-catenin (magenta) in 4-MU digits compared to controls at 14 DPA. Dashed line, border of the phalanx bone. Scale bars, 250 μm and 50 μm in magnified views. Right,

violin plots of Wnt and non-canonical Wnt scores. Data are mean ± SEM or median and quartiles (AFM data) and are representative of at least three independent experiments. Statistical significance was determined by two-tailed unpaired student's *t*-test (A and B). Additional details on statistics and reproducibility are shown in the Materials and Methods.

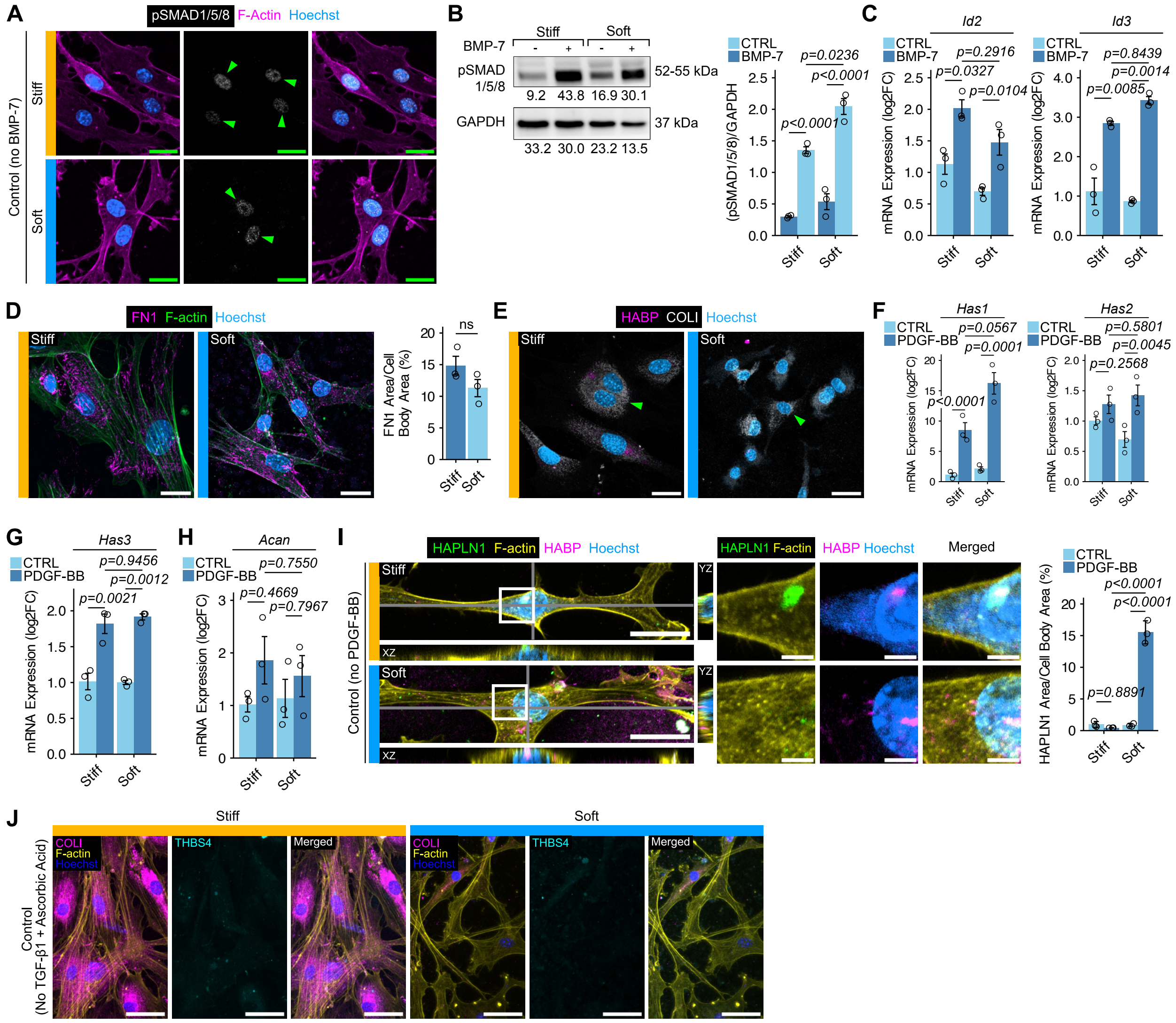

Fig. S6. The influence of substrate stiffness on BMP signaling and hyaluronic acid biosynthesis pathways.

(**A**) Immunofluorescence of pSMAD1/5/8 (white) in fibroblasts cultured on stiff (50 kPa) or soft (0.7 kPa) hydrogels without BMP-7 as controls, related to Fig. 4A. Green arrowheads, nuclear pSMAD1/5/8 signal. Scale bars, 20 µm. (**B**) Left, immunoblotting of pSMAD1/5/8. Values below the blots represent integrated band intensities. Right, quantification of integrated band intensities. n = 3 independent experiments. (**C**) qPCR of *Id2* and *Id3* gene expression. n = 3 independent experiments. (**D**) Left, immunofluorescence showing FN1 (magenta) in cultured fibroblasts. Scale bars, 20 µm. Right, quantification of FN1 area. n = 3 independent experiments. (**E**) Immunofluorescence of HABP (magenta) and COLI (white). Arrowheads, non-fibrillar, intracellular collagen. Scale bars, 25 µm. (**F-H**) qPCR of *Has1* and *Has2* in F, *Has3* in G, and *Acan* in H. n = 3 independent experiments. (**I**) Left, immunofluorescence of HAPLN1 (green) and HABP (magenta) without PDGF-BB as controls. Right, quantification of HAPLN1 area, n = 3 independent experiments and related to Fig. 4D. YZ and XZ images are orthogonal views. Scale bars, 25 µm and 10 µm in magnified views. (**J**) Immunofluorescence of COLI (magenta) and THBS4 (cyan) without TGF-β1 and ascorbic acid as controls, related to Fig. 4F. Scale bars, 50

μm. Data are mean ± SEM and are representative of at least three independent experiments. For all gene expression data, plots are shown as log2FC, with statistical analyses performed on -ΔΔCT values. Statistical significance was determined by two-way ANOVA (B, C, F, G, H, and I) with Tukey's multiple comparisons test or two-tailed unpaired student's *t*-test (D). Additional details on statistics and reproducibility are shown in the Materials and Methods.

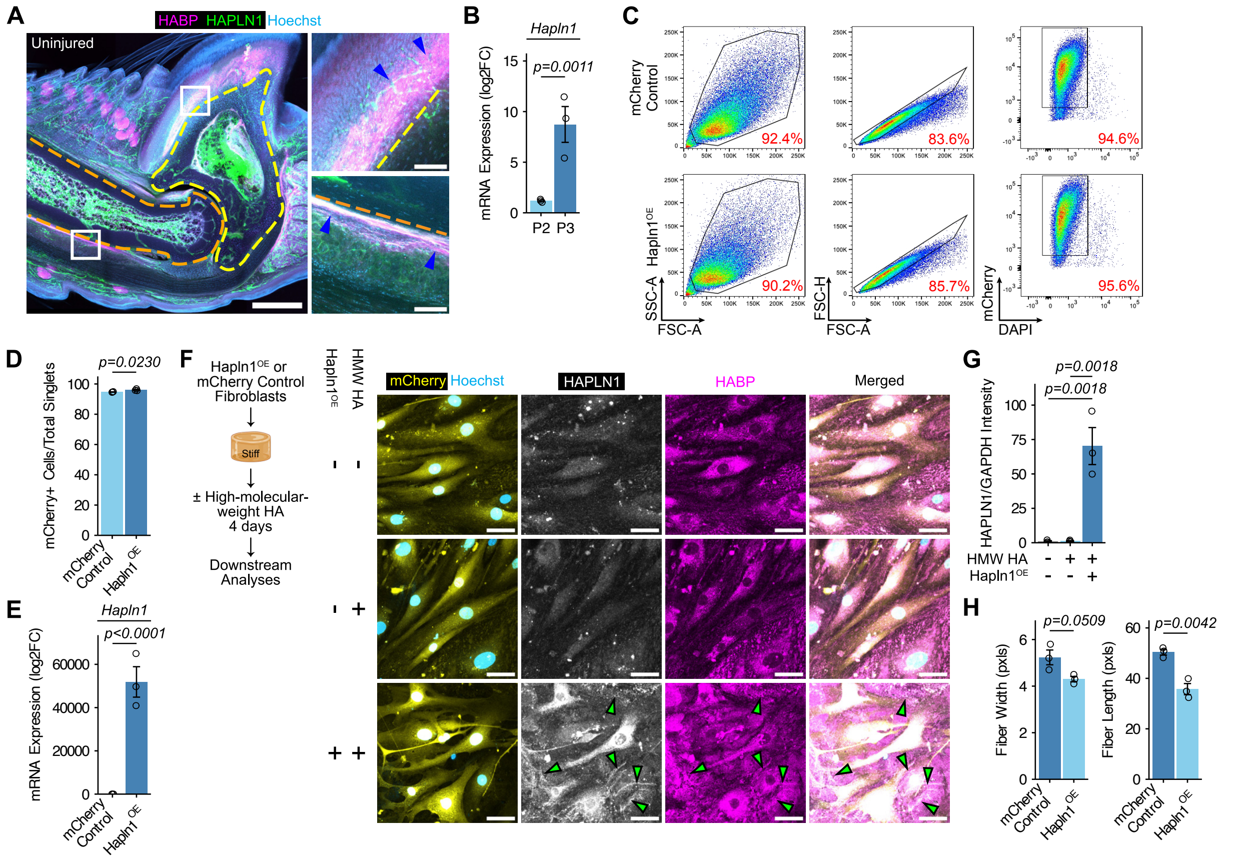

Fig. S7. *Hapln1* overexpression mediates ECM deposition and collagen fibrillogenesis.
(**A**) Immunofluorescence of HABP (magenta) and HAPLN1 (green) in an uninjured digit. Dashed lines, border of the phalanx bone. Arrowheads, periosteum. Scale bars, 300 µm and 50 µm in magnified views. (**B**) qPCR of *Hapln1* expression in uninjured primary P2 and P3 cells. n = 3 independent experiments. (**C**) Flow cytometric analysis of transduced mCherry Control and *Hapln1* overexpression (Hapln1^{OE}) fibroblasts. (**D**) Quantification of mCherry Control and Hapln1^{OE} infection efficiencies. n = 3 independent experiments. (**E**) qPCR of *Hapln1* gene expression in mCherry Control and Hapln1^{OE} fibroblasts. n = 3 independent experiments. (**F**) Left, strategy for testing the impact of *Hapln1* overexpression on pericellular hyaluronic acid, with or without high-molecular-weight (HMW) hyaluronic acid (HA) in a stiff (50 kPa) mechanical environment. Right, immunofluorescence of HAPLN1 (white) and HABP (magenta) in mCherry Control and Hapln1^{OE} fibroblasts, related to Fig. 5A. Arrowheads, regions of extracellular HAPLN1, co-localizing with aggregates of HA. Scale bars, 50 µm. (**G**) Quantification of HAPLN1 integrated band intensity, related to Fig. 5A. n = 3 independent experiments. (**H**) Quantification of collagen fiber width (left) and length (right) in mCherry control and Hapln1^{OE} fibroblasts, related to Fig. 5B. n = 3 independent experiments. Data are mean ± SEM and are representative of at least three independent experiments. Statistical significance was determined by two-tailed unpaired student's *t*-test (B, D, E, and H) or one-way ANOVA with Tukey's multiple comparisons test (G). Additional details on statistics and reproducibility are in the Materials and Methods. Schematic in F was created using BioRender (https://biorender.com).

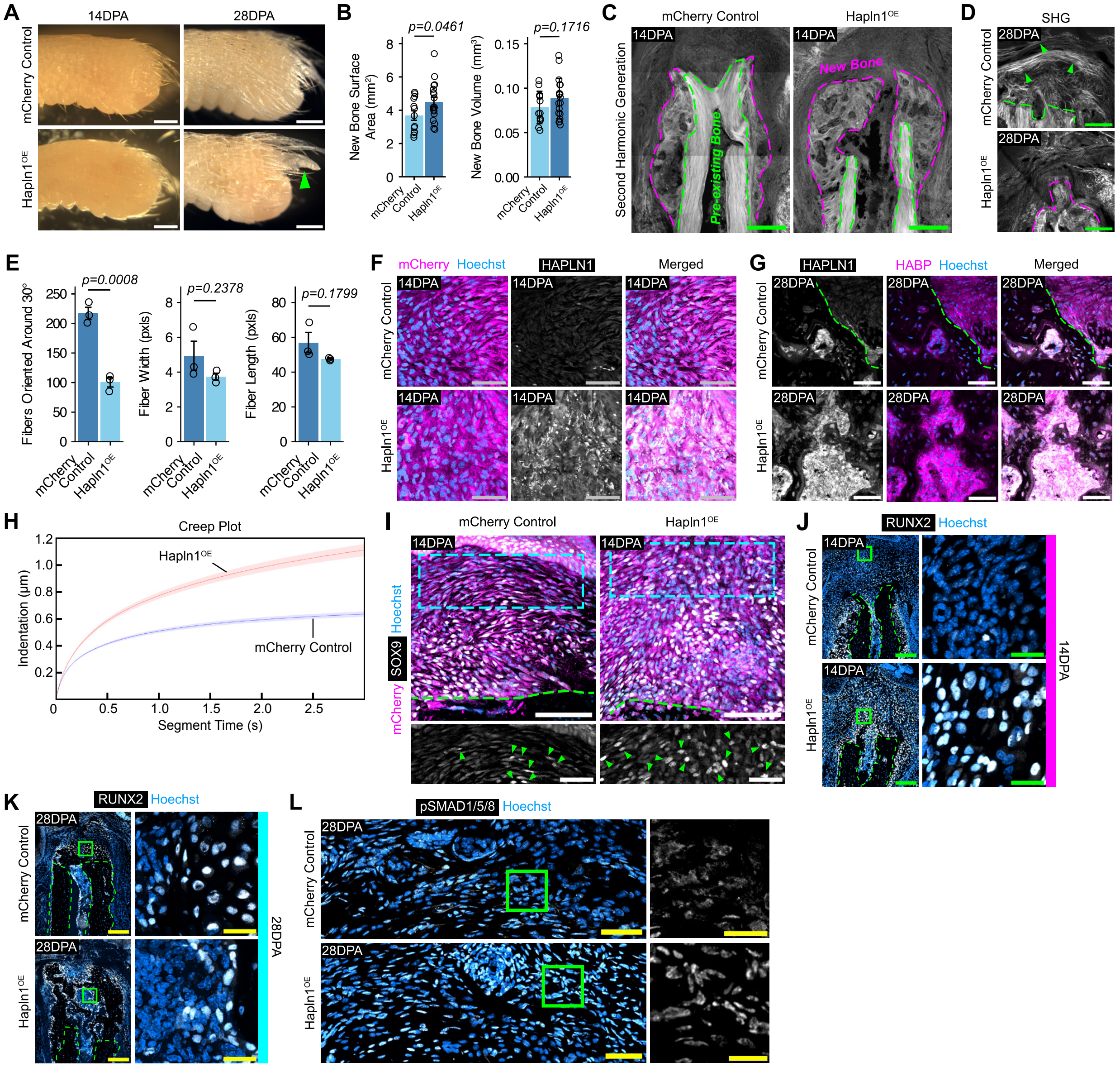

Fig. S8. *Hapln1* overexpression promotes digit repair while restraining fibrotic collagen. (**A**) Gross images of digits after mCherry Control or Hapln1^{OE} fibroblast transplantation 14 and 28 days post-amputation (DPA) through the second phalanx, related to Fig. 5D. Scale bars, 150 µm. (**B**) Quantification of new bone surface area and volume after mCherry Control or Hapln1^{OE} fibroblast transplantation 28 DPA, related to Fig. 5D. n = 18 digits per condition. (**C**) Second harmonic generation microscopy showing collagen fibers (white) in digits 14 DPA after mCherry Control or Hapln1^{OE} fibroblast transplantation. Green dashed line, border of the pre-existing bone; magenta, border of new bone. (**D** and **E**) Second harmonic generation (SHG) microscopy showing collagen fibers (white) in digits at 28 DPA after mCherry Control or Hapln1^{OE} fibroblast transplantation in D. Arrowheads, regions with prominent collagen fibers. Scale bars, 125 µm. In E, quantification of collagen fiber orientation, width, and length between conditions. n = 3 mice per condition. (**F** and **G**) Immunofluorescence of HAPLN1 (white) in F and G and HABP (magenta) in G. Scale bars, 50 µm (**H**) Atomic force microscopy creep plot, related to Fig. 5F. (**I**-**L**) Immunofluorescence of SOX9 (white) in I; RUNX2 (white) in J and K; and pSMAD1/5/8

(white) in L in digits 14 DPA after mCherry Control or Hapln1^{OE} fibroblast transplantation. Green dashed lines, border of the second phalanx bone. Scale bars, 100 µm and 50 µm in magnified views (I), 150 µm and 25 µm in magnified views (J and K), and 50 µm and 20 µm in magnified views (L). Data are mean ± SEM and are representative of at least three independent experiments. Statistical significance was determined by two-tailed unpaired student's *t*-test (B and E). Additional details on statistics and reproducibility are shown in the Materials and Methods.

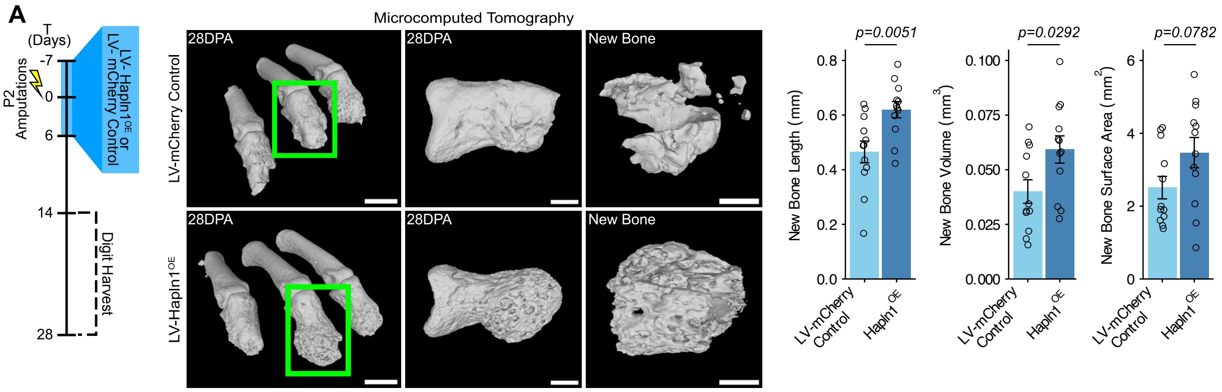

Fig. S9. Overexpression of *Hapln1* alone, without cells, promotes skeletal regeneration.
(A) Left, strategy to induce restorative repair of non-regenerating digits by injecting lentivirus overexpressing *Hapln1* (LV-Hapln1^{OE}) or a scrambled vector (LV-mCherry Control). Middle, microcomputed tomography analysis of skeletal morphologies of LV-mCherry control and LV-Hapln1^{OE} digits 28 DPA. Scale bars, 250 µm. Right, quantification of new bone length, volume, and surface area between conditions 28DPA, n = 12 digits per condition. Data are mean ± SEM and are representative of at least three independent experiments. Statistical significance was determined by two-tailed unpaired student's *t*-test in A. Additional details on statistics and reproducibility are in the Materials and Methods.

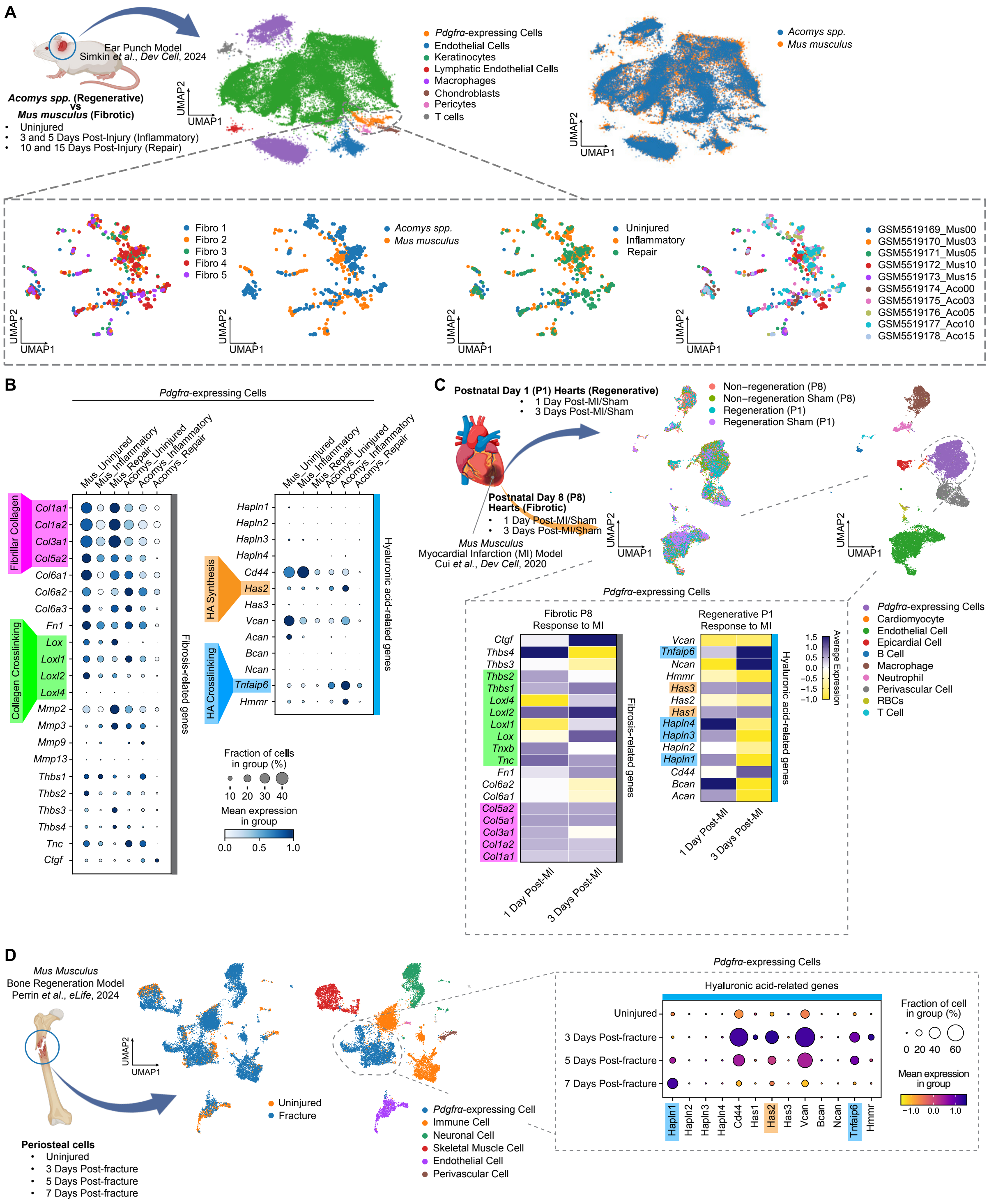

Fig. S10. HA-collagen dichotomy across mammalian models of regeneration and fibrosis. (**A**) Top left, schematic showing the strategy to compare the *Acomys'* regenerative response versus the *Mus musculus* fibrotic response to ear punch injuries at different timepoints. Top right, UMAP of the transcriptional identities of major cell types and species (*Acomys spp.* or *Mus musculus*) after ear pinna punch injuries. Bottom, UMAPs characterizing the *Pdgfrα*-expressing cell subset. (**B**) Dot plot of fibrosis-related and hyaluronic acid-related genes between mouse species. (**C**) Top

left, schematic showing the strategy to compare the regenerative response of postnatal day 1 (P1) versus the fibrotic response of postnatal day 8 (P8) mice hearts following myocardial infarction (MI) injury. Top right, UMAP of the myocardial infarction injury or sham condition and transcriptional identities of cardiac cells. Bottom, heat map of the fibrotic P8 response and HA signature in the regenerative P1 response among *Pdgfrα*-expressing cardiac fibroblasts. (**D**) Left, schematic showing the strategy to analyze the skeletal regeneration response in periosteal cells following bone fracture. Middle, UMAP of fracture condition and transcriptional identities of periosteal cells. Right, dot plot of hyaluronic-acid related genes during skeletal regeneration among *Pdgfrα*-expressing cells. The datasets in A-D were publicly available. Details on statistics and reproducibility, as well as Gene Expression Omnibus accession numbers, are in the Materials and Methods. Schematics in A, C, and D were created using BioRender (https://biorender.com).

Datasets	ncounts min filter	ncounts max filter	ngenes min filter	ngenes max filter	Mitochondrial percentage max filter	Scrublet filter score	Ambient RNA removal procedure
Regen 14DPA MS 1	0	50000	500	7000	10%	0.25	N/A
Regen 14DPA MS 1	0	50000	600	7000	10%	0.25	N/A
Regen 14DPA JL	0	70000	200	7000	10%	0.25	N/A
NonRegen14DPA 1	0	60000	500	4500	10%	0.25	N/A
NonRegen14DPA 2	0	75000	2200	8500	10%	0.25	SoupX
NonRegen14DPA 3	0	40000	2000	6000	10%	0.2	SoupX
4MU Control 1	1000	20000	400	5000	10%	0.2	Cellbender
4MU Control 2	4000	20000	600	6000	10%	0.2	Cellbender
4MU Treat 1	1000	25000	400	6000	10%	0.2	Cellbender
4MU Treat 2	1000	50000	1500	6000	10%	0.2	Cellbender
Uninjured P2.1	0	50000	1000	8000	10%	0.25	SoupX
Uninjured P2.2	0	50000	1700	7500	10%	0.25	SoupX
Uninjured P2.3	0	60000	300	8000	10%	0.25	N/A
Uninjured P3.1	0	20000	1200	4700	10%	0.2	SoupX
Uninjured P3.2	0	25000	2100	5000	10%	0.2	SoupX
Uninjured P3.3 (Johnson et al., 2020)	0	50000	200	5500	10%	0.25	N/A
Uninjured P3.4 (Storer et al. 2020)	0	20000	200	5000	10%	0.25	N/A
Uninjured P3.5 (Storer et al. 2020)	0	14000	200	3600	10%	0.25	N/A
Acomys vs Mus datasets	0	50000	200	5000	10%	0.25	N/A
Myocardial Infarction Datasets	0	50000	200	5000	10%	N/A	N/A
Bone Regeneration Datasets	0	50000	200	5000	10%	N/A	N/A

Table S1. Filtering parameters applied to single cell RNA sequencing datasets. Column 1 indicates the dataset; columns 2 and 3, the minimum (min) and maximum (max) number of RNA transcripts; columns 4 and 5, the minimum and maximum number of genes; column 6 the mitochondrial percentage (%) filter; column 7, Scrublet filter score for doublet detection; and column 8, the methodology used to remove ambient RNA. Regen, Regenerative; NonRegen, Non-regenerative; DPA, Days Post Amputation; 4MU, 4-methylumbelliferone; and N/A, Not Applicable.

Gene score name	Gene List
Matrisome	*5430419D17Rik, Abi3bp, Adipoq, Aebp1, Agrn, Ambn, Amelx, AW551984, Bglap2, Bglap3, Bmper, Bsph1, Bsph2, Cdcp2, Cilp, Cilp2, Coch, Colq, Comp, Creld1, Creld2, Crim1, Crispld1, Crispld2, Ctgf, Cthrc1, Cyr61, Ddx26b, Dmbt1, Dmp1, Dpt, Dspp, Ecm1, Ecm2, Edil3, Efemp1, Efemp2, Egfem1, Egflam, Eln, Emid1, Emilin1, Emilin2, Emilin3, Fbln1, Fbln2, Fbln5, Fbln7, Fbn1, Fbn2, Fga, Fgb, Fgg, Fgl1, Fgl2, Fn1, Fndc1, Fndc7, Fndc8, Fras1, Gas6, Gldn, Hmcn1, Hmcn2, Ibsp, Igfals, Igfbp1, Igfbp2, Igfbp3, Igfbp4, Igfbp5, Igfbp6, Igfbp7, Igfbpl1, Igsf10, Kcp, Lama1, Lama2, Lama3, Lama4, Lama5, Lamb1, Lamb2, Lamb3, Lamc1, Lamc2, Lamc3, Lgi1, Lgi2, Lgi3, Lgi4, Lrg1, Ltbp1, Ltbp2, Ltbp3, Ltbp4, Matn1, Matn2, Matn3, Matn4, Mepe, Mfap1a, Mfap1b, Mfap2, Mfap3, Mfap4, Mfap5, Mfge8, Mgp, Mmrn1, Mmrn2, Ndnf, Nell1, Nell2, Nid1, Nid2, Nov, Npnt, Ntn1, Ntn3, Ntn4, Ntn5, Ntng1, Ntng2, Oit3, Otog, Otogl, Otol1, Papln, Pcolce, Pcolce2, Postn, Pxdn, Reln, Rspo1, Rspo2, Rspo3, Rspo4, Sbspon, Slamf6, Slit1, Slit2, Slit3, Smoc1, Smoc2, Sned1, Sparc, Sparcl1, Spon1, Spon2, Spp1, Srpx, Srpx2, Sspo, Svep1, Tecta, Tectb, Tgfbi, Thbs1, Thbs2, Thbs3, Thbs4, Thsd4, Tinag, Tinagl1, Tnc, Tnfaip6, Tnn, Tnr, Tnxb, Tsku, Tspear, Vit, Vtn, Vwa1, Vwa2, Vwa3a, Vwa3b, Vwa5a, Vwa5b1, Vwa5b2, Vwa7, Vwa9, Vwce, Vwde, Vwf, Wisp1, Wisp2, Wisp3, Zp1, Zp2, Zp3, Zp3r, Zpld1, Col10a1, Col11a1, Col11a2, Col12a1, Col13a1, Col14a1, Col15a1, Col16a1, Col17a1, Col18a1, Col19a1, Col1a1, Col1a2, Col20a1, Col22a1, Col23a1, Col24a1, Col25a1, Col26a1, Col27a1, Col28a1, Col2a1, Col3a1, Col4a1, Col4a2, Col4a3, Col4a4, Col4a5, Col4a6, Col5a1, Col5a2, Col5a3, Col6a1, Col6a2, Col6a3, Col6a4, Col6a5, Col6a6, Col7a1, Col8a1, Col8a2, Col9a1, Col9a2, Col9a3, Acan, Aspn, Bcan, Bgn, Chad, Chadl, Dcn, Epyc, Esm1, Fmod, Hapln1, Hapln2, Hapln3, Hapln4, Hspg2, Impg1, Impg2, Kera, Lum, Ncan, Nepn, Nyx, Ogn, Omd, Optc, Podn, Podnl1, Prelp, Prg2, Prg3, Prg4, Spock1, Spock2, Spock3, Srgn, Vcan, Anxa1, Anxa10, Anxa11, Anxa13, Anxa2, Anxa3, Anxa4, Anxa5, Anxa6, Anxa7, Anxa8, Anxa9, C1qa, C1qb, C1qc, C1ql1, C1ql2, C1ql3, C1ql4, C1qtnf1, C1qtnf2, C1qtnf3, C1qtnf4, C1qtnf5, C1qtnf6, C1qtnf7, C1qtnf9, Cd209a, Cd209b, Cd209d, Clc, Clec10a, Clec11a, Clec12a, Clec12b, Clec14a, Clec18a, Clec1a, Clec1b, Clec2d, Clec2e, Clec2g, Clec2h, Clec2i, Clec2j, Clec2l, Clec3a, Clec3b, Clec4a1, Clec4a2, Clec4a3, Clec4a4, Clec4b1, Clec4b2, Clec4d, Clec4e, Clec4f, Clec4g, Clec4n, Clec5a, Clec7a, Clec9a, Colec10, Colec11, Colec12, Cspg4, Cspg5, Elfn1, Elfn2, Emcn, Fcna, Fcnb, Frem1, Frem2, Frem3, Gm7534, Gm9573, Gpc1, Gpc2, Gpc3, Gpc4, Gpc5, Gpc6, Grem1, Grifin, Hpx, Itln1, Itlnb, Lgals1, Lgals12, Lgals2, Lgals3, Lgals4, Lgals6, Lgals7, Lgals8, Lgals9, Lgalsl, Lman1, Lman1l, Mbl1, Mbl2, Muc1, Muc13, Muc15, Muc16, Muc19, Muc2, Muc20, Muc3, Muc4, Muc5ac, Muc5b, Muc6, Mucl1, Ovgp1, Parm1, Plxdc1, Plxdc2, Plxna1, Plxna2, Plxna3, Plxna4, Plxnb1, Plxnb2, Plxnb3, Plxnc1, Plxnd1, Prol1, Reg1, Reg2, Reg3a, Reg3b, Reg3d, Reg3g, Reg4, Sdc1, Sdc2, Sdc3, Sdc4, Sema3a, Sema3b, Sema3c, Sema3d, Sema3e, Sema3f, Sema3g, Sema4a, Sema4b, Sema4c, Sema4d, Sema4f, Sema4g, Sema5a, Sema5b, Sema6a, Sema6b, Sema6c, Sema6d, Sema7a, Sfta2, Sftpa1, Sftpb, Sftpc, Sftpd, 1810010H24Rik, 2010005H15Rik, 8030411F24Rik, 9230104L09Rik, A2m, Adam10, Adam11, Adam12, Adam15, Adam17, Adam18, Adam19, Adam1a, Adam1b, Adam2, Adam20, Adam21, Adam22, Adam23, Adam24, Adam25, Adam26a, Adam26b, Adam28, Adam29, Adam3, Adam30, Adam32, Adam33, Adam34, Adam39, Adam4, Adam5, Adam6a, Adam6b, Adam7, Adam8, Adam9, Adamdec1, Adamts1, Adamts10, Adamts12, Adamts13, Adamts14, Adamts15, Adamts16, Adamts17, Adamts18, Adamts19, Adamts2, Adamts20, Adamts3, Adamts4, Adamts5, Adamts6, Adamts7, Adamts8, Adamts9, Adamtsl1, Adamtsl2, Adamtsl3, Adamtsl4, Adamtsl5, Agt, AI182371, Ambp, Astl, BC048546, BC051665, BC100530, BC117090, Bmp1, Cd109, Cela1, Cela2a, Cela3b, Cpn2, Cst10, Cst11, Cst12, Cst13, Cst3, Cst6, Cst7, Cst8, Cst9, Csta1, Cstb, Cstl1, Cts3, Cts6, Cts7, Cts8, Ctsa, Ctsb, Ctsc, Ctsd, Ctse, Ctsf, Ctsg, Ctsh, Ctsj, Ctsk, Ctsl, Ctsll3, Ctsm, Ctso, Ctsq, Ctsr, Ctss, Ctsw, Ctsz, Egln1, Egln2, Egln3, Elane, F10, F12, F13a1, F13b, F2, F7, F9, Fam20a, Fam20b, Fam20c, Gm10334, Gm13011, Gm4758, Gm4787, Gm5347, Gm5409, Gm5416, Gm5483, Gm5771, Habp2, Hpse, Hpse2, Hrg, Htra1, Htra3, Htra4, Hyal1, Hyal2, Hyal3, Hyal4, Hyal5, Hyal6, Itih1, Itih2, Itih3, Itih4, Itih5, Kazald1, Kng1, Kng2, Ky, Lepre1, Leprel1, Leprel2, Lox, Loxl1, Loxl2, Loxl3, Loxl4, Masp1, Masp2, Mep1a, Mep1b, Mmp10, Mmp11, Mmp12, Mmp13,*

	Mmp14, Mmp15, Mmp16, Mmp17, Mmp19, Mmp1a, Mmp1b, Mmp2, Mmp20, Mmp21, Mmp23, Mmp24, Mmp25, Mmp27, Mmp28, Mmp3, Mmp7, Mmp8, Mmp9, Mug2, Ngly1, Ogfod1, Ogfod2, P4ha1, P4ha2, P4ha3, P4htm, Pamr1, Pappa, Pappa2, Pcsk5, Pcsk6, Plat, Plau, Plg, Plod1, Plod2, Plod3, Prss1, Prss12, Prss2, Prss3, Pzp, Serpina10, Serpina11, Serpina12, Serpina1a, Serpina1b, Serpina1c, Serpina1d, Serpina1e, Serpina1f, Serpina3a, Serpina3b, Serpina3c, Serpina3f, Serpina3g, Serpina3k, Serpina3m, Serpina3n, Serpina5, Serpina6, Serpina7, Serpina9, Serpinb10, Serpinb11, Serpinb12, Serpinb13, Serpinb1a, Serpinb1b, Serpinb1c, Serpinb2, Serpinb3a, Serpinb3b, Serpinb3c, Serpinb3d, Serpinb5, Serpinb6a, Serpinb6b, Serpinb6c, Serpinb6d, Serpinb7, Serpinb8, Serpinb9, Serpinb9b, Serpinb9c, Serpinb9d, Serpinb9e, Serpinb9f, Serpinb9g, Serpinc1, Serpind1, Serpine1, Serpine2, Serpine3, Serpinf1, Serpinf2, Serping1, Serpinh1, Serpini1, Serpini2, Slpi, Spam1, Spinkl, St14, Stfa1, Stfa2, Stfa2l1, Stfa3, Sulf1, Sulf2, Tgm1, Tgm2, Tgm3, Tgm4, Tgm5, Tgm6, Tgm7, Timp1, Timp2, Timp3, Timp4, Tll1, Tll2, Tmprss15, Tpbpa, Tpbpb, Try10, Try4, Try5, U06147, Amh, Angpt1, Angpt2, Angpt4, Angptl1, Angptl2, Angptl3, Angptl4, Angptl6, Angptl7, Areg, Artn, Bdnf, Bmp10, Bmp15, Bmp2, Bmp3, Bmp4, Bmp5, Bmp6, Bmp7, Bmp8a, Bmp8b, Brinp2, Brinp3, Btc, Cbln1, Cbln2, Cbln3, Cbln4, Ccbe1, Ccl1, Ccl11, Ccl12, Ccl17, Ccl19, Ccl2, Ccl20, Ccl21a, Ccl21b, Ccl21c, Ccl22, Ccl24, Ccl25, Ccl26, Ccl27a, Ccl28, Ccl3, Ccl4, Ccl5, Ccl6, Ccl7, Ccl8, Ccl9, Cfc1, Chrd, Chrdl1, Chrdl2, Clcf1, Cntf, Crhbp, Crlf1, Crlf3, Crnn, Csf1, Csf2, Csf3, Ctf1, Ctf2, Cx3cl1, Cxcl1, Cxcl10, Cxcl11, Cxcl12, Cxcl13, Cxcl14, Cxcl15, Cxcl2, Cxcl3, Cxcl5, Cxcl9, Dhh, Ebi3, Eda, Egf, Egfl6, Egfl7, Egfl8, Epgn, Epo, Ereg, Fam132a, Fam132b, Fasl, Fgf1, Fgf10, Fgf11, Fgf12, Fgf13, Fgf14, Fgf15, Fgf16, Fgf17, Fgf18, Fgf2, Fgf20, Fgf21, Fgf22, Fgf23, Fgf3, Fgf4, Fgf5, Fgf6, Fgf7, Fgf8, Fgf9, Fgfbp1, Fgfbp3, Figf, Flg, Flg2, Flt3l, Frzb, Fst, Fstl1, Fstl3, Gdf1, Gdf10, Gdf11, Gdf15, Gdf2, Gdf3, Gdf5, Gdf6, Gdf7, Gdf9, Gdnf, Gh, Gm13271, Gm13272, Gm13275, Gm13276, Gm13277, Gm13278, Gm13279, Gm13283, Gm13285, Gm13287, Gm13288, Gm13289, Gm13290, Gm13306, Gm5849, Hbegf, Hcfc1, Hcfc2, Hgf, Hgfac, Hhip, Hrnr, Ifna1, Ifna11, Ifna12, Ifna13, Ifna14, Ifna15, Ifna16, Ifna2, Ifna4, Ifna5, Ifna6, Ifna7, Ifna9, Ifnab, Ifnb1, Ifne, Ifng, Ifnk, Ifnz, Igf1, Igf2, Ihh, Il10, Il11, Il12a, Il12b, Il13, Il15, Il16, Il17a, Il17b, Il17c, Il17d, Il17f, Il18, Il19, Il1a, Il1b, Il1f10, Il1f5, Il1f6, Il1f8, Il1f9, Il1rn, Il2, Il20, Il22, Il23a, Il24, Il25, Il3, Il34, Il4, Il5, Il6, Il7, Il9, Inha, Inhba, Inhbb, Inhbc, Inhbe, Ins1, Ins2, Insl3, Insl5, Insl6, Ism1, Ism2, Kitl, Lefty1, Lefty2, Lep, Lif, Lta, Ltb, Mdk, Megf10, Megf11, Megf6, Megf8, Megf9, Mst1, Mstn, Ngf, Nodal, Nrg1, Nrg2, Nrg3, Nrg4, Nrtn, Ntf3, Ntf5, Osm, Pdgfa, Pdgfb, Pdgfc, Pdgfd, Pf4, Pgf, Pik3ip1, Ppbp, Prl, Prl2a1, Prl2b1, Prl2c1, Prl2c2, Prl2c3, Prl2c4, Prl2c5, Prl3a1, Prl3b1, Prl3c1, Prl3d1, Prl3d2, Prl3d3, Prl4a1, Prl5a1, Prl6a1, Prl7a1, Prl7a2, Prl7b1, Prl7c1, Prl7d1, Prl8a1, Prl8a2, Prl8a6, Prl8a8, Prl8a9, Pspn, Ptn, Rptn, S100a1, S100a10, S100a11, S100a13, S100a14, S100a16, S100a2, S100a3, S100a4, S100a5, S100a6, S100a7a, S100a8, S100a9, S100b, S100g, S100z, Scube1, Scube2, Scube3, Sfrp1, Sfrp2, Sfrp4, Sfrp5, Shh, Tchh, Tchhl1, Tdgf1, Tgfa, Tgfb1, Tgfb2, Tgfb3, Thpo, Tnf, Tnfsf10, Tnfsf11, Tnfsf12, Tnfsf12tnfsf13, Tnfsf13, Tnfsf13b, Tnfsf14, Tnfsf15, Tnfsf18, Tnfsf4, Tnfsf8, Tnfsf9, Tpo, Vegfa, Vegfb, Vegfc, Vwc2, Vwc2l, Wfikkn1, Wfikkn2, Wif1, Wnt1, Wnt10a, Wnt10b, Wnt11, Wnt16, Wnt2, Wnt2b, Wnt3, Wnt3a, Wnt4, Wnt5a, Wnt5b, Wnt6, Wnt7a, Wnt7b, Wnt8a, Wnt8b, Wnt9a, Wnt9b, Xcl1.
Fibrillar Collagen	*Col1a1, Col1a2, Col2a1, Col3a1, Col5a1, Col5a2, Col5a3, Col11a1, Col11a2, Col24a1, Col27a1*
Tenocyte Score	*Scx, Tnmd, Tnc, Col1a1, Col3a1, Thbs4, Dcn, Fmod, Egr1, Mkx*
Collagen and Proteoglycan	*Col10a1, Col11a1, Col11a2, Col12a1, Col13a1, Col14a1, Col15a1, Col16a1, Col17a1, Col18a1, Col19a1, Col1a1, Col1a2, Col20a1, Col22a1, Col23a1, Col24a1, Col25a1, Col26a1, Col27a1, Col28a1, Col2a1, Col3a1, Col4a1, Col4a2, Col4a3, Col4a4, Col4a5, Col4a6, Col5a1, Col5a2, Col5a3, Col6a1, Col6a2, Col6a3, Col6a4, Col6a5, Col6a6, Col7a1, Col8a1, Col8a2, Col9a1, Col9a2, Col9a3, Acan, Aspn, Bcan, Bgn, Chad, Chadl, Dcn, Epyc, Esm1, Fmod, Hapln1, Hapln2, Hapln3, Hapln4, Hspg2, Impg1, Impg2, Kera,*

	Lum, Ncan, Nepn, Nyx, Ogn, Omd, Optc, Podn, Podnl1, Prelp, Prg2, Prg3, Prg4, Spock1, Spock2, Spock3, Srgn, Vcan.
Wnt	*Axin2, Lgr5, Myc, Ccnd1, Tbx1, Tbx3, Cdx1, Mmp7, Wisp1, Wnt1, Wnt2, Wnt3, Wnt3a, Wnt8a.*
Non-canonical Wnt	*Wnt5a, Wnt5b, Wnt11, Wnt16.*

Table S2. Gene lists used for scoring. Column 1 shows the name of the scoring and column 2 the list of genes.

Antibody	SOURCE	IDENTIFIER
Goat polyclonal anti-CD31	Biotechne	Cat# AF3628; RRID:AB_2161028
Rat monoclonal anti-CD34	Thermo Fisher Scientific	Cat# 14-0341-82; RRID:AB_467210
Rabbit polyclonal anti-TNX	Abcam	Cat# ab111270; RRID:AB_10864625
Sheep polyclonal anti-THBS4	Biotechne	Cat# AF7860; RRID:AB_2924970
Rabbit polyclonal anti-COL1A1	Abcam	Cat# ab21286; RRID:AB_446161
Rat monoclonal anti-CD44	BD Biosciences	Cat# 550538; RRID:AB_393732
Rabbit polyclonal anti-ACAN	Merck	Cat# AB1031; RRID:AB_90460
Goat polyclonal anti-HAPLN1	Biotechne	Cat# AF2608; RRID:AB_2116135
Rabbit monoclonal anti-RUNX2	Abcam	Cat# ab192256; RRID:AB_2713945
Rabbit polyclonal anti-ARSI	Biotechne	Cat# NBP1-83678; RRID:AB_11010741
Rabbit polyclonal anti-SP7	Abcam	Cat# ab22552; RRID:AB_2194492
Rabbit polyclonal anti-Ki67	Abcam	Cat# ab15580; RRID:AB_443209
Rabbit polyclonal anti-pSMAD1/5/8	Merck	Cat# AB3848; RRID:AB_177439
Chicken polyclonal anti-mCherry	Abcam	Cat# ab205402; RRID:AB_2722769
Rabbit monoclonal anti-SOX9	Abcam	Cat# ab185966; RRID:AB_2728660
Rabbit polyclonal anti-GAPDH	Abcam	Cat# ab9485; RRID:AB_307275
Goat polyclonal anti-PDGFRα	R & D Systems	Cat# AF1062; RRID:AB_2236897
Rabbit polyclonal anti-SULF2	Proteintech	Cat# 12260-1-AP RRID:AB_11183762
Chicken monoclonal anti-K14	BioLegend	Cat# 906004 RRID:AB2616962
Rat monoclonal anti-Ki67	Invitrogen	Cat# 14-5698-82 RRID: AB_10854564
Rabbit monoclonal anti-β Catenin	Cell Signaling Technology	Cat# 8480 RRID: AB_11127855

Table S3. List of antibodies. Column 1 indicates the antibody target. Column 2 indicates the source, and Column 3 shows the catalog number and RRID.

E (kPa)	40% Acrylamide (µl)	2% Bis-acrylamide (µl)	2 M AHA (µl)	TEMED (µl)	10% APS (µl)	MiliQ H_2O (µl)	Total Volume (µl)
0.7	35	27.2	20	2.5	5	410.3	492.5
50	94	70.5	20	2.5	5	308	492.5

Table S4. Recipes for fabricating StemBond hydrogels of different stiffnesses. Column 1 indicates the stiffness, *E*, shown in kilopascal (kPa). Other reagents are shown in the following columns. AHA, 6-acrylamidohexanoic acid; TEMED, tetramethyl ethylenediamine; APS, ammonium persulfate; H_2O, water; µl, microliter.

Gene	Primer sequences
Primers for *Has1* cDNA amplification:	5'-3' CATGGGCTATGCTACCAAGT (Forward)<br>5'-3' TCAACCAACGAAGGAAGGA (Reverse)
Primers for *Has2* cDNA amplification:	5'-3' TCAGCGAAGTTATGGGCAGG (Forward)<br>5'-3' TCTGTCTCACCAGGTCCCTT (Reverse)
Primers for *Has3* cDNA amplification:	5'-3' CAATCGCCAGGAAGATACCTAC (Forward)<br>5'-3' GGAAATTGCTACGCCACACAA (Reverse)
Primers for *Cd44* cDNA amplification:	5'-3' ATCAGCAGATCGATTTGAATGTAA (Forward)<br>5'-3' CATTTCCTTCTATGAACCCATACC (Reverse)
Primers for *Hapln1* cDNA amplification:	5'-3' CCCCCGTCTACTTGTGGAAG (Forward)<br>5'-3' TCCTGAGCCAAATGCTGTAGG (Reverse)
Primers for *Acan* cDNA amplification:	5'-3' CCCAAGTGGAGTAGCTGAGG (Forward)<br>5'-3' AGACACAGTGGGGAAACCTG (Reverse)
Primers for *Id1* cDNA amplification:	5'-3' CCTAGCTGTTCGCTGAAGGC (Forward)<br>5'-3' GTAGAGCAGGACGTTCACCT (Reverse)
Primers for *Id2* cDNA amplification:	5'-3' TCCGGTGAGGTCCGTTAGG (Forward)<br>5'-3' CAGACTCATCGGGTCGTCC (Reverse)
Primers for *Id3* cDNA amplification:	5'-3' CTGTCGGAACGTAGCCTGG (Forward)<br>5'-3' GTGGTTCATGTCGTCCAAGAG (Reverse)
Primers for *Gapdh* cDNA amplification:	5'-3' AGGTCGGTGTGAACGGATTTG (Forward)<br>5'-3' TGTAGACCATGTAGTTGAGGTCA (Reverse)

Table S5. Primers used for qPCR experiments. Column 1 contains the name of the gene of interest. Column 2 shows the forward and reverse oligonucleotide sequences for each gene.

Software and algorithms		
Cell Ranger Single-Cell Software Suite (Version 8.0.0)	10X Genomics	https://www.10xgenomics.com/support/software/cell-ranger/latest/tutorials/cr-tutorial-in; RRID:SCR_017344
R (Version 4.4.1)	The R Foundation	https://www.r-project.org/; RRID:SCR_001905
Python (Version 3.10)	Python Software Foundation	https://www.python.org/; RRID:SCR_008394
SoupX (Version 1.6.2)	Young et al. (71)	https://github.com/constantAmateur/SoupX?tab=readme-ov-file; RRID:SCR_019193
CellBender (Version 0.3.2)	Fleming et al. (72)	https://github.com/broadinstitute/CellBender; RRID:SCR_025990
Scrublet (Version 0.2.3)	Wolock et al. (73)	https://github.com/swolock/scrublet; RRID:SCR_018098
Scanpy (Version 1.9.6)	Wolf et al. (67)	https://scanpy.readthedocs.io/en/stable/; RRID:SCR_018139
scVI (Version 1.0.4)	Lopez et al. (74)	https://docs.scvi-tools.org/en/latest/index.html
Speckle (Version 1.2.0)	Phipson et al. (75)	https://github.com/Oshlack/speckle

Table S6. Software and algorithms. Column 1 contains the software or algorithm used. Column 2 and 3 contains the source of the package.

Plasmid	Sequence	Supplier
pLV[Exp]-EF1A-mHapln1-mCherry (Hapln1^{OE})	AATGTAGTCTTATGCAATACTCTTGTAGTCTTGCAACATGGTAACGATG<br>AGTTAGCAACATGCCTTACAAGGAGAGAAAAAGCACCGTGCATGCCGA<br>TTGGTGGAAGTAAGGTGGTACGATCGTGCCTTATTAGGAAGGCAACAG<br>ACGGGTCTGACATGGATTGGACGAACCACTGAATTGCCGCATTGCAGA<br>GATATTGTATTTAAGTGCCTAGCTCGATACATAAACGGGTCTCTCTGGT<br>TAGACCAGATCTGAGCCTGGGAGCTCTCTGGCTAACTAGGGAACCCACT<br>GCTTAAGCCTCAATAAAGCTTGCCTTGAGTGCTTCAAGTAGTGTGTGCC<br>CGTCTGTTGTGTGACTCTGGTAACTAGAGATCCCTCAGACCCTTTTAGTC<br>AGTGTGGAAAATCTCTAGCAGTGGCGCCCGAACAGGGACTTGAAAGCG<br>AAAGGGAAACCAGAGGAGCTCTCTCGACGCAGGACTCGGCTTGCTGAA<br>GCGCGCACGGCAAGAGGCGAGGGGCGGCGACTGGTGAGTACGCCAAA<br>AATTTTGACTAGCGGAGGCTAGAAGGAGAGAGATGGGTGCGAGAGCGT<br>CAGTATTAAGCGGGGGAGAATTAGATCGCGATGGGAAAAAATTCGGTT<br>AAGGCCAGGGGGAAAGAAAAAATATAAATTAAAACATATAGTATGGGC<br>AAGCAGGGAGCTAGAACGATTCGCAGTTAATCCTGGCCTGTTAGAAAC<br>ATCAGAAGGCTGTAGACAAATACTGGGACAGCTACAACCATCCCTTCA<br>GACAGGATCAGAAGAACTTAGATCATTATATAATACAGTAGCAACCCT<br>CTATTGTGTGCATCAAAGGATAGAGATAAAAGACACCAAGGAAGCTTT<br>AGACAAGATAGAGGAAGAGCAAAACAAAAGTAAGACCACCGCACAGC<br>AAGCGGCCGCTGATCTTCAGACCTGGAGGAGGAGATATGAGGGACAAT<br>TGGAGAAGTGAATTATATAAATATAAAGTAGTAAAAATTGAACCATTA<br>GGAGTAGCACCCACCAAGGCAAAGAGAAGAGTGGTGCAGAGAGAAAA<br>AAGAGCAGTGGGAATAGGAGCTTTGTTCCTTGGGTTCTTGGGAGCAGCA<br>GGAAGCACTATGGGCGCAGCGTCAATGACGCTGACGGTACAGGCCAGA<br>CAATTATTGTCTGGTATAGTGCAGCAGCAGAACAATTTGCTGAGGGCTA<br>TTGAGGCGCAACAGCATCTGTTGCAACTCACAGTCTGGGGCATCAAGCA<br>GCTCCAGGCAAGAATCCTGGCTGTGGAAAGATACCTAAAGGATCAACA<br>GCTCCTGGGGATTTGGGGTTGCTCTGGAAAACTCATTTGCACCACTGCT<br>GTGCCTTGGAATGCTAGTTGGAGTAATAAATCTCTGGAACAGATTTGGA<br>ATCACACGACCTGGATGGAGTGGGACAGAGAAATTAACAATTACACAA<br>GCTTAATACACTCCTTAATTGAAGAATCGCAAAACCAGCAAGAAAAGA<br>ATGAACAAGAATTATTGGAATTAGATAAATGGGCAAGTTTGTGGAATT<br>GGTTTAACATAACAAATTGGCTGTGGTATATAAAATTATTCATAATGAT<br>AGTAGGAGGCTTGGTAGGTTTAAGAATAGTTTTTGCTGTACTTTCTATA<br>GTGAATAGAGTTAGGCAGGGATATTCACCATTATCGTTTCAGACCCACC<br>TCCCAACCCCGAGGGGACCCGACAGGCCCGAAGGAATAGAAGAAGAA<br>GGTGGAGAGAGAGACAGAGACAGATCCATTCGATTAGTGAACGGATCT<br>CGACGGTATCGCTAGCTTTTAAAAGAAAAGGGGGGATTGGGGGGTACA<br>GTGCAGGGGAAAGAATAGTAGACATAATAGCAACAGACATACAAACTA<br>AAGAATTACAAAAACAAATTACAAAAATTCAAAATTTTACTAGTGATTA<br>TCGGATCAACTTTGTATAGAAAAGTTGGGCTCCGGTGCCCGTCAGTGGG<br>CAGAGCGCACATCGCCCACAGTCCCCGAGAAGTTGGGGGGAGGGGTCG<br>GCAATTGAACCGGTGCCTAGAGAAGGTGGCGCGGGGTAAACTGGGAAA<br>GTGATGTCGTGTACTGGCTCCGCCTTTTTCCCGAGGGTGGGGGAGAACC<br>GTATATAAGTGCAGTAGTCGCCGTGAACGTTCTTTTTCGCAACGGGTTT<br>GCCGCCAGAACACAGGTAAGTGCCGTGTGTGGTTCCCGCGGGCCTGGC<br>CTCTTTACGGGTTATGGCCCTTGCGTGCCTTGAATTACTTCCACCTGGCT<br>GCAGTACGTGATTCTTGATCCCGAGCTTCGGGTTGGAAGTGGGTGGGAG<br>AGTTCGAGGCCTTGCGCTTAAGGAGCCCCTTCGCCTCGTGCTTGAGTTG<br>AGGCCTGGCCTGGGCGCTGGGGCCGCCGCGTGCGAATCTGGTGGCACC<br>TTCGCGCCTGTCTCGCTGCTTTCGATAAGTCTCTAGCCATTTAAAATTTT	VectorBuilder

	TGATGACCTGCTGCGACGCTTTTTTTCTGGCAAGATAGTCTTGTAAATGC<br>GGGCCAAGATCTGCACACTGGTATTTCGGTTTTTGGGGCCGCGGGCGGC<br>GACGGGGCCCGTGCGTCCCAGCGCACATGTTCGGCGAGGCGGGGCCTG<br>CGAGCGCGGCCACCGAGAATCGGACGGGGGTAGTCTCAAGCTGGCCGG<br>CCTGCTCTGGTGCCTGGTCTCGCGCCGCCGTGTATCGCCCCGCCCTGGG<br>CGGCAAGGCTGGCCCGGTCGGCACCAGTTGCGTGAGCGGAAAGATGGC<br>CGCTTCCCGGCCCTGCTGCAGGGAGCTCAAAATGGAGGACGCGGCGCT<br>CGGGAGAGCGGGCGGGTGAGTCACCCACACAAAGGAAAAGGGCCTTTC<br>CGTCCTCAGCCGTCGCTTCATGTGACTCCACGGAGTACCGGGCGCCGTC<br>CAGGCACCTCGATTAGTTCTCGAGCTTTTGGAGTACGTCGTCTTTAGGTT<br>GGGGGGAGGGGTTTTATGCGATGGAGTTTCCCCACACTGAGTGGGTGG<br>AGACTGAAGTTAGGCCAGCTTGGCACTTGATGTAATTCTCCTTGGAATT<br>TGCCCTTTTTGAGTTTGGATCTTGGTTCATTCTCAAGCCTCAGACAGTGG<br>TTCAAAGTTTTTTTCTTCCATTTCAGGTGTCGTGACAAGTTTGTACAAAA<br>AAGCAGGCTGCCACCATGAGAAGTCTGCTTCTCCTGGTGCTGATTTCAG<br>TCTGTTGGGCTGACCACCACCTTTCAGACAGCTACACTCCTCCAGATCA<br>AGACAGAGTTATTCACATCCAAGCAGAAAATGGCCCCCGTCTACTTGTG<br>GAAGCAGAACAAGCCAAGGTCTTCTCTCACCGAGGTGGCAACGTGACA<br>CTGCCATGCAAATTTTATCGAGACCCTACAGCATTTGGCTCAGGAATCC<br>ACAAAATCCGCATCAAGTGGACCAAGCTAACTTCAGATTACCTCAGGG<br>AAGTAGATGTCTTCGTTTCCATGGGCTATCACAAGAAGACCTATGGAGG<br>CTATCAAGGTCGAGTGTTTCTGAAGGGAGGCAGTGATAATGATGCCTCC<br>CTGGTCATCACGGATCTTACCCTGGAGGATTATGGGAGATATAAATGTG<br>AGGTGATTGAAGGGCTAGAAGATGATACTGCTGTGGTGGCATTGGAGT<br>TACAAGGTGTGGTGTTCCCTTACTTTCCACGACTGGGACGCTACAATCT<br>TAACTTTCACGAGGCACGCCAGGCTTGTCTGGACCAGGACGCAGTGATT<br>GCTTCCTTTGACCAGCTGTACGATGCCTGGCGGGGTGGGCTGGACTGGT<br>GCAATGCTGGCTGGCTCAGTGATGGATCTGTGCAGTACCCAATCACCAA<br>ACCACGAGAGCCCTGCGGGGGCCAAAACACGGTGCCTGGAGTCAGGAA<br>CTACGGGTTTTGGGACAAGGATAAAAGCAGATATGACGTTTTCTGTTTT<br>ACATCCAACTTCAACGGCCGATTTTACTACCTGATCCACCCCACCAAAC<br>TCACCTACGATGAGGCGGTGCAAGCTTGTCTCAATGACGGTGCTCAGAT<br>CGCGAAAGTGGGCCAGATATTTGCTGCCTGGAAGCTTCTGGGCTATGAC<br>CGCTGCGATGCCGGCTGGCTAGCGGATGGCAGCGTCCGCTATCCTATTT<br>CTCGACCAAGAAGGCGCTGCAGTCCGACTGAGGCTGCAGTGCGCTTTGT<br>AGGTTTCCCAGATAAAAAGCATAAGCTATACGGGGTCTATTGCTTCAGA<br>GCATACAACTGAACCCAGCTTTCTTGTACAAAGTGGTGATAATCGAATT<br>CCGATAATCAACCTCTGGATTACAAAATTTGTGAAAGATTGACTGGTAT<br>TCTTAACTATGTTGCTCCTTTTACGCTATGTGGATACGCTGCTTTAATGC<br>CTTTGTATCATGCTATTGCTTCCCGTATGGCTTTCATTTTCTCCTCCTTGT<br>ATAAATCCTGGTTGCTGTCTCTTTATGAGGAGTTGTGGCCCGTTGTCAG<br>GCAACGTGGCGTGGTGTGCACTGTGTTTGCTGACGCAACCCCCACTGGT<br>TGGGGCATTGCCACCACCTGTCAGCTCCTTTCCGGGACTTTCGCTTTCCC<br>CCTCCCTATTGCCACGGCGGAACTCATCGCCGCCTGCCTTGCCCGCTGC<br>TGGACAGGGGCTCGGCTGTTGGGCACTGACAATTCCGTGGTGTTGTCGG<br>GGAAGCTGACGTCCTTTCCATGGCTGCTCGCCTGTGTTGCCACCTGGAT<br>TCTGCGCGGGACGTCCTTCTGCTACGTCCCTTCGGCCCTCAATCCAGCG<br>GACCTTCCTTCCCGCGGCCTGCTGCCGGCTCTGCGGCCTCTTCCGCGTCT<br>TCGCCTTCGCCCTCAGACGAGTCGGATCTCCCTTTGGGCCGCCTCCCCG<br>CATCGGGAATTCCCGCGGTTCGAACGCGTTGACATTGATTATTGACTAG<br>TTATTAATAGTAATCAATTACGGGGTCATTAGTTCATAGCCCATATATG<br>GAGTTCCGCGTTACATAACTTACGGTAAATGGCCCGCCTGGCTGACCGC<br>CCAACGACCCCCGCCCATTGACGTCAATAATGACGTATGTTCCCATAGT<br>AACGCCAATAGGGACTTTCCATTGACGTCAATGGGTGGAGTATTTACGG<br>TAAACTGCCCACTTGGCAGTACATCAAGTGTATCATATGCCAAGTACGC<br>CCCCTATTGACGTCAATGACGGTAAATGGCCCGCCTGGCATTATGCCCA	

	GTACATGACCTTATGGGACTTTCCTACTTGGCAGTACATCTACGTATTA<br>GTCATCGCTATTACCATGGTGATGCGGTTTTGGCAGTACATCAATGGGC<br>GTGGATAGCGGTTTGACTCACGGGGATTTCCAAGTCTCCACCCCATTGA<br>CGTCAATGGGAGTTTGTTTTGGCACCAAAATCAACGGGACTTTCCAAAA<br>TGTCGTAACAACTCCGCCCCATTGACGCAAATGGGCGGTAGGCGTGTAC<br>GGTGGGAGGTCTATATAAGCAGAGCTCTCTGGCTAACTAGAGAACCCA<br>CTGCGCCACCATGGTGAGCAAGGGCGAGGAGGATAACATGGCCATCAT<br>CAAGGAGTTCATGCGCTTCAAGGTGCACATGGAGGGCTCCGTGAACGG<br>CCACGAGTTCGAGATCGAGGGCGAGGGCGAGGGCCGCCCCTACGAGGG<br>CACCCAGACCGCCAAGCTGAAGGTGACCAAGGGTGGCCCCCTGCCCTT<br>CGCCTGGGACATCCTGTCCCCTCAGTTCATGTACGGCTCCAAGGCCTAC<br>GTGAAGCACCCCGCCGACATCCCCGACTACTTGAAGCTGTCCTTCCCCG<br>AGGGCTTCAAGTGGGAGCGCGTGATGAACTTCGAGGACGGCGGCGTGG<br>TGACCGTGACCCAGGACTCCTCCCTGCAGGACGGCGAGTTCATCTACAA<br>GGTGAAGCTGCGCGGCACCAACTTCCCCTCCGACGGCCCCGTAATGCAG<br>AAGAAGACCATGGGCTGGGAGGCCTCCTCCGAGCGGATGTACCCCGAG<br>GACGGCGCCCTGAAGGGCGAGATCAAGCAGAGGCTGAAGCTGAAGGA<br>CGGCGGCCACTACGACGCTGAGGTCAAGACCACCTACAAGGCCAAGAA<br>GCCCGTGCAGCTGCCCGGCGCCTACAACGTCAACATCAAGTTGGACATC<br>ACCTCCCACAACGAGGACTACACCATCGTGGAACAGTACGAACGCGCC<br>GAGGGCCGCCACTCCACCGGCGGCATGGACGAGCTGTACAAGTAAGGT<br>ACCTTTAAGACCAATGACTTACAAGGCAGCTGTAGATCTTAGCCACTTT<br>TTAAAAGAAAAGGGGGGACTGGAAGGGCTAATTCACTCCCAACGAAGA<br>CAAGATCTGCTTTTTGCTTGTACTGGGTCTCTCTGGTTAGACCAGATCTG<br>AGCCTGGGAGCTCTCTGGCTAACTAGGGAACCCACTGCTTAAGCCTCAA<br>TAAAGCTTGCCTTGAGTGCTTCAAGTAGTGTGTGCCCGTCTGTTGTGTG<br>ACTCTGGTAACTAGAGATCCCTCAGACCCTTTTAGTCAGTGTGGAAAAT<br>CTCTAGCAGTAGTAGTTCATGTCATCTTATTATTCAGTATTTATAACTTG<br>CAAAGAAATGAATATCAGAGAGTGAGAGGAACTTGTTTATTGCAGCTT<br>ATAATGGTTACAAATAAAGCAATAGCATCACAAATTTCACAAATAAAG<br>CATTTTTTTCACTGCATTCTAGTTGTGGTTTGTCCAAACTCATCAATGTA<br>TCTTATCATGTCTGGCTCTAGCTATCCCGCCCCTAACTCCGCCCATCCCG<br>CCCCTAACTCCGCCCAGTTCCGCCCATTCTCCGCCCCATGGCTGACTAAT<br>TTTTTTTATTTATGCAGAGGCCGAGGCCGCCTCGGCCTCTGAGCTATTCC<br>AGAAGTAGTGAGGAGGCTTTTTTGGAGGCCTAGGGACGTACCCAATTC<br>GCCCTATAGTGAGTCGTATTACGCGCGCTCACTGGCCGTCGTTTTACAA<br>CGTCGTGACTGGGAAAACCCTGGCGTTACCCAACTTAATCGCCTTGCAG<br>CACATCCCCCTTTCGCCAGCTGGCGTAATAGCGAAGAGGCCCGCACCGA<br>TCGCCCTTCCCAACAGTTGCGCAGCCTGAATGGCGAATGGGACGCGCCC<br>TGTAGCGGCGCATTAAGCGCGGCGGGTGTGGTGGTTACGCGCAGCGTG<br>ACCGCTACACTTGCCAGCGCCCTAGCGCCCGCTCCTTTCGCTTTCTTCCC<br>TTCCTTTCTCGCCACGTTCGCCGGCTTTCCCCGTCAAGCTCTAAATCGGG<br>GGCTCCCTTTAGGGTTCCGATTTAGTGCTTTACGGCACCTCGACCCCAA<br>AAAACTTGATTAGGGTGATGGTTCACGTAGTGGGCCATCGCCCTGATAG<br>ACGGTTTTTCGCCCTTTGACGTTGGAGTCCACGTTCTTTAATAGTGGACT<br>CTTGTTCCAAACTGGAACAACACTCAACCCTATCTCGGTCTATTCTTTTG<br>ATTTATAAGGGATTTTGCCGATTTCGGCCTATTGGTTAAAAAATGAGCT<br>GATTTAACAAAAATTTAACGCGAATTTTAACAAAATATTAACGCTTACA<br>ATTTAGGTGGCACTTTTCGGGGAAATGTGCGCGGAACCCCTATTTGTTT<br>ATTTTTCTAAATACATTCAAATATGTATCCGCTCATGAGACAATAACCC<br>TGATAAATGCTTCAATAATATTGAAAAAGGAAGAGTATGAGTATTCAA<br>CATTTCCGTGTCGCCCTTATTCCCTTTTTTGCGGCATTTTGCCTTCCTGTT<br>TTTGCTCACCCAGAAACGCTGGTGAAAGTAAAAGATGCTGAAGATCAG<br>TTGGGTGCACGAGTGGGTTACATCGAACTGGATCTCAACAGCGGTAAG<br>ATCCTTGAGAGTTTTCGCCCCGAAGAACGTTTTCCAATGATGAGCACTT<br>TTAAAGTTCTGCTATGTGGCGCGGTATTATCCCGTATTGACGCCGGGCA	

	AGAGCAACTCGGTCGCCGCATACACTATTCTCAGAATGACTTGGTTGAG<br>TACTCACCAGTCACAGAAAAGCATCTTACGGATGGCATGACAGTAAGA<br>GAATTATGCAGTGCTGCCATAACCATGAGTGATAACACTGCGGCCAACT<br>TACTTCTGACAACGATCGGAGGACCGAAGGAGCTAACCGCTTTTTTGCA<br>CAACATGGGGGATCATGTAACTCGCCTTGATCGTTGGGAACCGGAGCTG<br>AATGAAGCCATACCAAACGACGAGCGTGACACCACGATGCCTGTAGCA<br>ATGGCAACAACGTTGCGCAAACTATTAACTGGCGAACTACTTACTCTAG<br>CTTCCCGGCAACAATTAATAGACTGGATGGAGGCGGATAAAGTTGCAG<br>GACCACTTCTGCGCTCGGCCCTTCCGGCTGGCTGGTTTATTGCTGATAA<br>ATCTGGAGCCGGTGAGCGTGGGTCTCGCGGTATCATTGCAGCACTGGGG<br>CCAGATGGTAAGCCCTCCCGTATCGTAGTTATCTACACGACGGGGAGTC<br>AGGCAACTATGGATGAACGAAATAGACAGATCGCTGAGATAGGTGCCT<br>CACTGATTAAGCATTGGTAACTGTCAGACCAAGTTTACTCATATATACT<br>TTAGATTGATTTAAAACTTCATTTTTAATTTAAAAGGATCTAGGTGAAG<br>ATCCTTTTTGATAATCTCATGACCAAAATCCCTTAACGTGAGTTTTCGTT<br>CCACTGAGCGTCAGACCCCGTAGAAAAGATCAAAGGATCTTCTTGAGA<br>TCCTTTTTTTCTGCGCGTAATCTGCTGCTTGCAAACAAAAAAACCACCG<br>CTACCAGCGGTGGTTTGTTTGCCGGATCAAGAGCTACCAACTCTTTTTCC<br>GAAGGTAACTGGCTTCAGCAGAGCGCAGATACCAAATACTGTTCTTCTA<br>GTGTAGCCGTAGTTAGGCCACCACTTCAAGAACTCTGTAGCACCGCCTA<br>CATACCTCGCTCTGCTAATCCTGTTACCAGTGGCTGCTGCCAGTGGCGA<br>TAAGTCGTGTCTTACCGGGTTGGACTCAAGACGATAGTTACCGGATAAG<br>GCGCAGCGGTCGGGCTGAACGGGGGGTTCGTGCACACAGCCCAGCTTG<br>GAGCGAACGACCTACACCGAACTGAGATACCTACAGCGTGAGCTATGA<br>GAAAGCGCCACGCTTCCCGAAGAGAGAAAGGCGGACAGGTATCCGGTA<br>AGCGGCAGGGTCGGAACAGGAGAGCGCACGAGGGAGCTTCCAGGGGG<br>AAACGCCTGGTATCTTTATAGTCCTGTCGGGTTTCGCCACCTCTGACTTG<br>AGCGTCGATTTTTGTGATGCTCGTCAGGGGGGCGGAGCCTATGGAAAA<br>ACGCCAGCAACGCGGCCTTTTTACGGTTCCTGGCCTTTTGCTGGCCTTTT<br>GCTCACATGTTCTTTCCTGCGTTATCCCCTGATTCTGTGGATAACCGTAT<br>TACCGCCTTTGAGTGAGCTGATACCGCTCGCCGCAGCCGAACGACCGAG<br>CGCAGCGAGTCAGTGAGCGAGGAAGCGGAAGAGCGCCCAATACGCAA<br>ACCGCCTCTCCCCGCGCGTTGGCCGATTCATTAATGCAGCTGGCACGAC<br>AGGTTTCCCGACTGGAAAGCGGGCAGTGAGCGCAACGCAATTAATGTG<br>AGTTAGCTCACTCATTAGGCACCCCAGGCTTTACACTTTATGCTTCCGGC<br>TCGTATGTTGTGTGGAATTGTGAGCGGATAACAATTTCACACAGGAAAC<br>AGCTATGACCATGATTACGCCAAGCGCGCAATTAACCCTCACTAAAGG<br>GAACAAAAGCTGGAGCTGCAAGCTT	
pLV[Exp]-EF1A-Scramble-mCherry (mCherry control)	AATGTAGTCTTATGCAATACTCTTGTAGTCTTGCAACATGGTAACGATG<br>AGTTAGCAACATGCCTTACAAGGAGAGAAAAAGCACCGTGCATGCCGA<br>TTGGTGGAAGTAAGGTGGTACGATCGTGCCTTATTAGGAAGGCAACAG<br>ACGGGTCTGACATGGATTGGACGAACCACTGAATTGCCGCATTGCAGA<br>GATATTGTATTTAAGTGCCTAGCTCGATACATAAACGGGTCTCTCTGGT<br>TAGACCAGATCTGAGCCTGGGAGCTCTCTGGCTAACTAGGGAACCCACT<br>GCTTAAGCCTCAATAAAGCTTGCCTTGAGTGCTTCAAGTAGTGTGTGCC<br>CGTCTGTTGTGTGACTCTGGTAACTAGAGATCCCTCAGACCCTTTTAGTC<br>AGTGTGGAAAATCTCTAGCAGTGGCGCCCGAACAGGGACTTGAAAGCG<br>AAAGGGAAACCAGAGGAGCTCTCTCGACGCAGGACTCGGCTTGCTGAA<br>GCGCGCACGGCAAGAGGCGAGGGGCGGCGACTGGTGAGTACGCCAAA<br>AATTTTGACTAGCGGAGGCTAGAAGGAGAGAGATGGGTGCGAGAGCGT<br>CAGTATTAAGCGGGGGAGAATTAGATCGCGATGGGAAAAAATTCGGTT<br>AAGGCCAGGGGGAAAGAAAAAATATAAATTAAAACATATAGTATGGGC<br>AAGCAGGGAGCTAGAACGATTCGCAGTTAATCCTGGCCTGTTAGAAAC<br>ATCAGAAGGCTGTAGACAAATACTGGGACAGCTACAACCATCCCTTCA<br>GACAGGATCAGAAGAACTTAGATCATTATATAATACAGTAGCAACCCT<br>CTATTGTGTGCATCAAAGGATAGAGATAAAAGACACCAAGGAAGCTTT	VectorBuilder

	AGACAAGATAGAGGAAGAGCAAAACAAAAGTAAGACCACCGCACAGC<br>AAGCGGCCGCTGATCTTCAGACCTGGAGGAGGAGATATGAGGGACAAT<br>TGGAGAAGTGAATTATATAAATATAAAGTAGTAAAAATTGAACCATTA<br>GGAGTAGCACCCACCAAGGCAAAGAGAAGAGTGGTGCAGAGAGAAAA<br>AAGAGCAGTGGGAATAGGAGCTTTGTTCCTTGGGTTCTTGGGAGCAGCA<br>GGAAGCACTATGGGCGCAGCGTCAATGACGCTGACGGTACAGGCCAGA<br>CAATTATTGTCTGGTATAGTGCAGCAGCAGAACAATTTGCTGAGGGCTA<br>TTGAGGCGCAACAGCATCTGTTGCAACTCACAGTCTGGGGCATCAAGCA<br>GCTCCAGGCAAGAATCCTGGCTGTGGAAAGATACCTAAAGGATCAACA<br>GCTCCTGGGGATTTGGGGTTGCTCTGGAAAACTCATTTGCACCACTGCT<br>GTGCCTTGGAATGCTAGTTGGAGTAATAAATCTCTGGAACAGATTTGGA<br>ATCACACGACCTGGATGGAGTGGGACAGAGAAATTAACAATTACACAA<br>GCTTAATACACTCCTTAATTGAAGAATCGCAAAACCAGCAAGAAAAGA<br>ATGAACAAGAATTATTGGAATTAGATAAATGGGCAAGTTTGTGGAATT<br>GGTTTAACATAACAAATTGGCTGTGGTATATAAAATTATTCATAATGAT<br>AGTAGGAGGCTTGGTAGGTTTAAGAATAGTTTTTGCTGTACTTTCTATA<br>GTGAATAGAGTTAGGCAGGGATATTCACCATTATCGTTTCAGACCCACC<br>TCCCAACCCCGAGGGGACCCGACAGGCCCGAAGGAATAGAAGAAGAA<br>GGTGGAGAGAGAGACAGAGACAGATCCATTCGATTAGTGAACGGATCT<br>CGACGGTATCGCTAGCTTTTAAAAGAAAAGGGGGGATTGGGGGGTACA<br>GTGCAGGGGAAAGAATAGTAGACATAATAGCAACAGACATACAAACTA<br>AAGAATTACAAAAACAAATTACAAAAATTCAAAATTTTACTAGTGATTA<br>TCGGATCAACTTTGTATAGAAAAGTTGGGCTCCGGTGCCCGTCAGTGGG<br>CAGAGCGCACATCGCCCACAGTCCCCGAGAAGTTGGGGGGAGGGGTCG<br>GCAATTGAACCGGTGCCTAGAGAAGGTGGCGCGGGGTAAACTGGGAAA<br>GTGATGTCGTGTACTGGCTCCGCCTTTTTCCCGAGGGTGGGGGAGAACC<br>GTATATAAGTGCAGTAGTCGCCGTGAACGTTCTTTTTCGCAACGGGTTT<br>GCCGCCAGAACACAGGTAAGTGCCGTGTGTGGTTCCCGCGGGCCTGGC<br>CTCTTTACGGGTTATGGCCCTTGCGTGCCTTGAATTACTTCCACCTGGCT<br>GCAGTACGTGATTCTTGATCCCGAGCTTCGGGTTGGAAGTGGGTGGGAG<br>AGTTCGAGGCCTTGCGCTTAAGGAGCCCCTTCGCCTCGTGCTTGAGTTG<br>AGGCCTGGCCTGGGCGCTGGGGCCGCCGCGTGCGAATCTGGTGGCACC<br>TTCGCGCCTGTCTCGCTGCTTTCGATAAGTCTCTAGCCATTTAAAATTTT<br>TGATGACCTGCTGCGACGCTTTTTTTCTGGCAAGATAGTCTTGTAAATGC<br>GGGCCAAGATCTGCACACTGGTATTTCGGTTTTTGGGGCCGCGGGCGGC<br>GACGGGGCCCGTGCGTCCCAGCGCACATGTTCGGCGAGGCGGGGCCTG<br>CGAGCGCGGCCACCGAGAATCGGACGGGGGTAGTCTCAAGCTGGCCGG<br>CCTGCTCTGGTGCCTGGTCTCGCGCCGCCGTGTATCGCCCCGCCCTGGG<br>CGGCAAGGCTGGCCCGGTCGGCACCAGTTGCGTGAGCGGAAAGATGGC<br>CGCTTCCCGGCCCTGCTGCAGGGAGCTCAAAATGGAGGACGCGGCGCT<br>CGGGAGAGCGGGCGGGTGAGTCACCCACACAAAGGAAAAGGGCCTTTC<br>CGTCCTCAGCCGTCGCTTCATGTGACTCCACGGAGTACCGGGCGCCGTC<br>CAGGCACCTCGATTAGTTCTCGAGCTTTTGGAGTACGTCGTCTTTAGGTT<br>GGGGGGAGGGGTTTTATGCGATGGAGTTTCCCCACACTGAGTGGGTGG<br>AGACTGAAGTTAGGCCAGCTTGGCACTTGATGTAATTCTCCTTGGAATT<br>TGCCCTTTTTGAGTTTGGATCTTGGTTCATTCTCAAGCCTCAGACAGTGG<br>TTCAAAGTTTTTTTCTTCCATTTCAGGTGTCGTGACAAGTTTGTACAAAA<br>AAGCAGGCTGCCACCGTCGTTTTACAACGTCGTGACTGGGAAAACCCTG<br>GCGTTACCCAACTTAATCGCCTTGCAGCACATCCCCCTTTCGCCAGCTG<br>GCGTAATAGCGAAGAGGCCCGCACCGATCGCCCTTCCCAACAGTTGCG<br>CAGCCTGAACGGCGAGTGGCGCTTTGCCTGGTTTCCGGCACCAGAAGCG<br>GTGCCGGAAAGCTGGCTGGAGTGCGATCTTCCTGAGGCCGATACTGTCG<br>TCGTCCCCTCAAACTGGCAGACCCAGCTTTCTTGTACAAAGTGGTGATA<br>ATCGAATTCCGATAATCAACCTCTGGATTACAAAATTTGTGAAAGATTG<br>ACTGGTATTCTTAACTATGTTGCTCCTTTTACGCTATGTGGATACGCTGC<br>TTTAATGCCTTTGTATCATGCTATTGCTTCCCGTATGGCTTTCATTTTCTC	

	CTCCTTGTATAAATCCTGGTTGCTGTCTCTTTATGAGGAGTTGTGGCCCG<br>TTGTCAGGCAACGTGGCGTGGTGTGCACTGTGTTTGCTGACGCAACCCC<br>CACTGGTTGGGGCATTGCCACCACCTGTCAGCTCCTTTCCGGGACTTTC<br>GCTTTCCCCCTCCCTATTGCCACGGCGGAACTCATCGCCGCCTGCCTTGC<br>CCGCTGCTGGACAGGGGCTCGGCTGTTGGGCACTGACAATTCCGTGGTG<br>TTGTCGGGGAAGCTGACGTCCTTTCCATGGCTGCTCGCCTGTGTTGCCA<br>CCTGGATTCTGCGCGGGACGTCCTTCTGCTACGTCCCTTCGGCCCTCAAT<br>CCAGCGGACCTTCCTTCCCGCGGCCTGCTGCCGGCTCTGCGGCCTCTTCC<br>GCGTCTTCGCCTTCGCCCTCAGACGAGTCGGATCTCCCTTTGGGCCGCCT<br>CCCCGCATCGGGAATTCCCGCGGTTCGAACGCGTTGACATTGATTATTG<br>ACTAGTTATTAATAGTAATCAATTACGGGGTCATTAGTTCATAGCCCAT<br>ATATGGAGTTCCGCGTTACATAACTTACGGTAAATGGCCCGCCTGGCTG<br>ACCGCCCAACGACCCCCGCCCATTGACGTCAATAATGACGTATGTTCCC<br>ATAGTAACGCCAATAGGGACTTTCCATTGACGTCAATGGGTGGAGTATT<br>TACGGTAAACTGCCCACTTGGCAGTACATCAAGTGTATCATATGCCAAG<br>TACGCCCCCTATTGACGTCAATGACGGTAAATGGCCCGCCTGGCATTAT<br>GCCCAGTACATGACCTTATGGGACTTTCCTACTTGGCAGTACATCTACG<br>TATTAGTCATCGCTATTACCATGGTGATGCGGTTTTGGCAGTACATCAA<br>TGGGCGTGGATAGCGGTTTGACTCACGGGGATTTCCAAGTCTCCACCCC<br>ATTGACGTCAATGGGAGTTTGTTTTGGCACCAAAATCAACGGGACTTTC<br>CAAAATGTCGTAACAACTCCGCCCCATTGACGCAAATGGGCGGTAGGC<br>GTGTACGGTGGGAGGTCTATATAAGCAGAGCTCTCTGGCTAACTAGAG<br>AACCCACTGCGCCACCATGGTGAGCAAGGGCGAGGAGGATAACATGGC<br>CATCATCAAGGAGTTCATGCGCTTCAAGGTGCACATGGAGGGCTCCGTG<br>AACGGCCACGAGTTCGAGATCGAGGGCGAGGGCGAGGGCCGCCCCTAC<br>GAGGGCACCCAGACCGCCAAGCTGAAGGTGACCAAGGGTGGCCCCCTG<br>CCCTTCGCCTGGGACATCCTGTCCCCTCAGTTCATGTACGGCTCCAAGG<br>CCTACGTGAAGCACCCCGCCGACATCCCCGACTACTTGAAGCTGTCCTT<br>CCCCGAGGGCTTCAAGTGGGAGCGCGTGATGAACTTCGAGGACGGCGG<br>CGTGGTGACCGTGACCCAGGACTCCTCCCTGCAGGACGGCGAGTTCATC<br>TACAAGGTGAAGCTGCGCGGCACCAACTTCCCCTCCGACGGCCCCGTAA<br>TGCAGAAGAAGACCATGGGCTGGGAGGCCTCCTCCGAGCGGATGTACC<br>CCGAGGACGGCGCCCTGAAGGGCGAGATCAAGCAGAGGCTGAAGCTGA<br>AGGACGGCGGCCACTACGACGCTGAGGTCAAGACCACCTACAAGGCCA<br>AGAAGCCCGTGCAGCTGCCCGGCGCCTACAACGTCAACATCAAGTTGG<br>ACATCACCTCCCACAACGAGGACTACACCATCGTGGAACAGTACGAAC<br>GCGCCGAGGGCCGCCACTCCACCGGCGGCATGGACGAGCTGTACAAGG<br>GCTCCGGAGAGGGCAGGGGAAGTCTTCTAACATGCGGGGACGTGGAGG<br>AAAATCCCGGCCCCATGACCGAGTACAAGCCCACGGTGCGCCTCGCCA<br>CCCGCGACGACGTCCCCAGGGCCGTACGCACCCTCGCCGCCGCGTTCGC<br>CGACTACCCCGCCACGCGCCACACCGTCGATCCGGACCGCCACATCGA<br>GCGGGTCACCGAGCTGCAAGAACTCTTCCTCACGCGCGTCGGGCTCGAC<br>ATCGGCAAGGTGTGGGTCGCGGACGACGGCGCCGCGGTGGCGGTCTGG<br>ACCACGCCGGAGAGCGTCGAAGCGGGGGCGGTGTTCGCCGAGATCGGC<br>CCGCGCATGGCCGAGTTGAGCGGTTCCCGGCTGGCCGCGCAGCAACAG<br>ATGGAAGGCCTCCTGGCGCCGCACCGGCCCAAGGAGCCCGCGTGGTTC<br>CTGGCCACCGTCGGCGTCTCGCCCGACCACCAGGGCAAGGGTCTGGGC<br>AGCGCCGTCGTGCTCCCCGGAGTGGAGGCGGCCGAGCGCGCCGGGGTG<br>CCCGCCTTCCTGGAGACCTCCGCGCCCCGCAACCTCCCCTTCTACGAGC<br>GGCTCGGCTTCACCGTCACCGCCGACGTCGAGGTGCCCGAAGGACCGC<br>GCACCTGGTGCATGACCCGCAAGCCCGGTGCCTGAGGTACCTTTAAGAC<br>CAATGACTTACAAGGCAGCTGTAGATCTTAGCCACTTTTTAAAAGAAAA<br>GGGGGGACTGGAAGGGCTAATTCACTCCCAACGAAGACAAGATCTGCT<br>TTTTGCTTGTACTGGGTCTCTCTGGTTAGACCAGATCTGAGCCTGGGAG<br>CTCTCTGGCTAACTAGGGAACCCACTGCTTAAGCCTCAATAAAGCTTGC<br>CTTGAGTGCTTCAAGTAGTGTGTGCCCGTCTGTTGTGTGACTCTGGTAAC	

	TAGAGATCCCTCAGACCCTTTTAGTCAGTGTGGAAAATCTCTAGCAGTA<br>GTAGTTCATGTCATCTTATTATTCAGTATTTATAACTTGCAAAGAAATGA<br>ATATCAGAGAGTGAGAGGAACTTGTTTATTGCAGCTTATAATGGTTACA<br>AATAAAGCAATAGCATCACAAATTTCACAAATAAAGCATTTTTTTCACT<br>GCATTCTAGTTGTGGTTTGTCCAAACTCATCAATGTATCTTATCATGTCT<br>GGCTCTAGCTATCCCGCCCCTAACTCCGCCCATCCCGCCCCTAACTCCG<br>CCCAGTTCCGCCCATTCTCCGCCCCATGGCTGACTAATTTTTTTTATTTA<br>TGCAGAGGCCGAGGCCGCCTCGGCCTCTGAGCTATTCCAGAAGTAGTG<br>AGGAGGCTTTTTTGGAGGCCTAGGGACGTACCCAATTCGCCCTATAGTG<br>AGTCGTATTACGCGCGCTCACTGGCCGTCGTTTTACAACGTCGTGACTG<br>GGAAAACCCTGGCGTTACCCAACTTAATCGCCTTGCAGCACATCCCCCT<br>TTCGCCAGCTGGCGTAATAGCGAAGAGGCCCGCACCGATCGCCCTTCCC<br>AACAGTTGCGCAGCCTGAATGGCGAATGGGACGCGCCCTGTAGCGGCG<br>CATTAAGCGCGGCGGGTGTGGTGGTTACGCGCAGCGTGACCGCTACACT<br>TGCCAGCGCCCTAGCGCCCGCTCCTTTCGCTTTCTTCCCTTCCTTTCTCG<br>CCACGTTCGCCGGCTTTCCCCGTCAAGCTCTAAATCGGGGGCTCCCTTT<br>AGGGTTCCGATTTAGTGCTTTACGGCACCTCGACCCCAAAAAACTTGAT<br>TAGGGTGATGGTTCACGTAGTGGGCCATCGCCCTGATAGACGGTTTTTC<br>GCCCTTTGACGTTGGAGTCCACGTTCTTTAATAGTGGACTCTTGTTCCAA<br>ACTGGAACAACACTCAACCCTATCTCGGTCTATTCTTTTGATTTATAAGG<br>GATTTTGCCGATTTCGGCCTATTGGTTAAAAAATGAGCTGATTTAACAA<br>AAATTTAACGCGAATTTTAACAAAATATTAACGCTTACAATTTAGGTGG<br>CACTTTTCGGGGAAATGTGCGCGGAACCCCTATTTGTTTATTTTTCTAAA<br>TACATTCAAATATGTATCCGCTCATGAGACAATAACCCTGATAAATGCT<br>TCAATAATATTGAAAAAGGAAGAGTATGAGTATTCAACATTTCCGTGTC<br>GCCCTTATTCCCTTTTTTGCGGCATTTTGCCTTCCTGTTTTTGCTCACCCA<br>GAAACGCTGGTGAAAGTAAAAGATGCTGAAGATCAGTTGGGTGCACGA<br>GTGGGTTACATCGAACTGGATCTCAACAGCGGTAAGATCCTTGAGAGTT<br>TTCGCCCCGAAGAACGTTTTCCAATGATGAGCACTTTTAAAGTTCTGCT<br>ATGTGGCGCGGTATTATCCCGTATTGACGCCGGGCAAGAGCAACTCGGT<br>CGCCGCATACACTATTCTCAGAATGACTTGGTTGAGTACTCACCAGTCA<br>CAGAAAAGCATCTTACGGATGGCATGACAGTAAGAGAATTATGCAGTG<br>CTGCCATAACCATGAGTGATAACACTGCGGCCAACTTACTTCTGACAAC<br>GATCGGAGGACCGAAGGAGCTAACCGCTTTTTTGCACAACATGGGGGA<br>TCATGTAACTCGCCTTGATCGTTGGGAACCGGAGCTGAATGAAGCCATA<br>CCAAACGACGAGCGTGACACCACGATGCCTGTAGCAATGGCAACAACG<br>TTGCGCAAACTATTAACTGGCGAACTACTTACTCTAGCTTCCCGGCAAC<br>AATTAATAGACTGGATGGAGGCGGATAAAGTTGCAGGACCACTTCTGC<br>GCTCGGCCCTTCCGGCTGGCTGGTTTATTGCTGATAAATCTGGAGCCGG<br>TGAGCGTGGGTCTCGCGGTATCATTGCAGCACTGGGGCCAGATGGTAA<br>GCCCTCCCGTATCGTAGTTATCTACACGACGGGGAGTCAGGCAACTATG<br>GATGAACGAAATAGACAGATCGCTGAGATAGGTGCCTCACTGATTAAG<br>CATTGGTAACTGTCAGACCAAGTTTACTCATATATACTTTAGATTGATTT<br>AAAACTTCATTTTTAATTTAAAAGGATCTAGGTGAAGATCCTTTTTGAT<br>AATCTCATGACCAAAATCCCTTAACGTGAGTTTTCGTTCCACTGAGCGT<br>CAGACCCCGTAGAAAAGATCAAAGGATCTTCTTGAGATCCTTTTTTTCT<br>GCGCGTAATCTGCTGCTTGCAAACAAAAAAACCACCGCTACCAGCGGT<br>GGTTTGTTTGCCGGATCAAGAGCTACCAACTCTTTTTCCGAAGGTAACT<br>GGCTTCAGCAGAGCGCAGATACCAAATACTGTTCTTCTAGTGTAGCCGT<br>AGTTAGGCCACCACTTCAAGAACTCTGTAGCACCGCCTACATACCTCGC<br>TCTGCTAATCCTGTTACCAGTGGCTGCTGCCAGTGGCGATAAGTCGTGT<br>CTTACCGGGTTGGACTCAAGACGATAGTTACCGGATAAGGCGCAGCGG<br>TCGGGCTGAACGGGGGGTTCGTGCACACAGCCCAGCTTGGAGCGAACG<br>ACCTACACCGAACTGAGATACCTACAGCGTGAGCTATGAGAAAGCGCC<br>ACGCTTCCCGAAGAGAGAAAGGCGGACAGGTATCCGGTAAGCGGCAGG<br>GTCGGAACAGGAGAGCGCACGAGGGAGCTTCCAGGGGGAAACGCCTG	

	GTATCTTTATAGTCCTGTCGGGTTTCGCCACCTCTGACTTGAGCGTCGATTTTTGTGATGCTCGTCAGGGGGGCGGAGCCTATGGAAAAACGCCAGCAACGCGGCCTTTTTACGGTTCCTGGCCTTTTGCTGGCCTTTTGCTCACATGTTCTTTCCTGCGTTATCCCCTGATTCTGTGGATAACCGTATTACCGCCTTTGAGTGAGCTGATACCGCTCGCCGCAGCCGAACGACCGAGCGCAGCGAGTCAGTGAGCGAGGAAGCGGAAGAGCGCCCAATACGCAAACCGCCTCTCCCCGCGCGTTGGCCGATTCATTAATGCAGCTGGCACGACAGGTTTCCCGACTGGAAAGCGGGCAGTGAGCGCAACGCAATTAATGTGAGTTAGCTCACTCATTAGGCACCCCAGGCTTTACACTTTATGCTTCCGGCTCGTATGTTGTGTGGAATTGTGAGCGGATAACAATTTCACACAGGAAACAGCTATGACCATGATTACGCCAAGCGCGCAATTAACCCTCACTAAAGGGAACAAAAGCTGGAGCTGCAAGCTT	

Table S7. List of plasmids and their sequences. Column 1 contains the plasmid name. Column 2 and 3 contains the plasmid sequence and source, respectively.
